# A comparison of Bayesian information borrowing methods in basket trials and a novel proposal of modified exchangeability‐nonexchangeability method

**DOI:** 10.1002/sim.9867

**Published:** 2023-08-23

**Authors:** Libby Daniells, Pavel Mozgunov, Alun Bedding, Thomas Jaki

**Affiliations:** ^1^ STOR‐i Centre for Doctoral Training, Department of Mathematics and Statistics Lancaster University Lancaster UK; ^2^ MRC Biostatistics Unit University of Cambridge Cambridge UK; ^3^ Roche Products Ltd Welwyn Garden City UK; ^4^ Faculty of Informatics and Data Science University of Regensburg Regensburg Germany

**Keywords:** basket trial, Bayesian hierarchical model, error control, information borrowing, master protocol

## Abstract

Recent innovation in trial design to improve study efficiency has led to the development of basket trials in which a single therapeutic treatment is tested on several patient populations, each of which forms a basket. In a common setting, patients across all baskets share a genetic marker and as such, an assumption can be made that all patients may have a homogeneous response to treatments. Bayesian information borrowing procedures utilize this assumption to draw on information regarding the response in one basket when estimating the response rate in others. This can improve power and precision of estimates particularly in the presence of small sample sizes, however, can come at a cost of biased estimates and an inflation of error rates, bringing into question validity of trial conclusions. We review and compare the performance of several Bayesian borrowing methods, namely: the Bayesian hierarchical model (BHM), calibrated Bayesian hierarchical model (CBHM), exchangeability‐nonexchangeability (EXNEX) model and a Bayesian model averaging procedure. A generalization of the CBHM is made to account for unequal sample sizes across baskets. We also propose a modification of the EXNEX model that allows for better control of a type I error. The proposed method uses a data‐driven approach to account for the homogeneity of the response data, measured through Hellinger distances. Through an extensive simulation study motivated by a real basket trial, for both equal and unequal sample sizes across baskets, we show that in the presence of a basket with a heterogeneous response, unlike the other methods discussed, this model can control type I error rates to a nominal level whilst yielding improved power.

## 1 INTRODUCTION

Over the past decade there have been advancements in cancer genomics and refinement in diagnostic techniques, leading to the increased interest in the field of personalized medicine in which treatments are targeted to a specific genetic makeup.^1^ It would be infeasible to test these treatments on each of their targeted biomarkers in individual studies due to financial and time constraints. Master protocols have been proposed to tackle this problem. This term refers to trial designs that allow the testing of multiple treatments and/or multiple disease types in parallel under a single protocol.^2^

Basket trials are a form of master protocol that are usually implemented in phase II of the drug development process within which a small number of patients are recruited to the study to determine efficacy of a treatment. Such a trial tests a single therapeutic treatment on several patient population sub‐groups, each of which form a basket. Commonly, patients across all baskets share a genetic change/biomarker but each basket consists of patients with different diseases. One benefit of this trial design is its ability to test treatments which would traditionally not warrant their own investigation for their targeted patient population, due to their rarity and limited sample size.

As various groups in a basket trial share a common genetic aberration, a reasonable assumption can be made—known as the exchangeability assumption—that sub‐groups may have a homogeneous response to the treatment.^3^ Specifically, the exchangeability assumption means that patients may be switched between exchangeable baskets without changing the overall value of the estimated basket treatment effects.^4^ This exchangeability of patients across baskets implies that all baskets can be viewed as random samples from the same model.^5^, ^6^ There is some uncertainty surrounding the definition of nonexchangeability, in this article it is utilized to describe baskets between which no information is shared (usually due to heterogeneity in treatment effects). With this exchangeability assumption in mind, a concept known as “information borrowing” can be used to draw on information regarding the response in one basket when estimating the response rate in others. This has the potential to increase power and precision of estimates, especially in the presence of small basket sample sizes. A desirable feature of such information borrowing methods is the ability to solely borrow between baskets with similar treatment effects, but not from those which are heterogeneous, as it may bias estimates and inflate the error rate resulting in a higher chance of a misleading conclusion. One would therefore like a method that has the ability to improve the power and precision of estimates while having control over error rates through only borrowing between homogeneous baskets.

Recently, numerous methods for information borrowing within the analysis of basket trials have been proposed. These methods either borrow information across all baskets such as the Bayesian hierarchical model (BHM^7^) and the calibrated Bayesian hierarchical model (CBHM^8^), while others borrow between subsets of baskets, for example, the exchangeability nonexchangeability model (EXNEX^9^) and a Bayesian model averaging approach (BMA^10^). This article provides a summary, alongside an extensive comparison of each method through simulation studies motivated by the VE‐BASKET study, which consider both equal and unequal sample sizes across baskets. The consideration of unequal sample sizes is rare within the literature but an important aspect that needs to be considered when applying the models to clinical trial data.

We also propose an extension to the EXNEX model, which takes into account pairwise similarity between baskets' response rates through Hellinger distances in order to update the borrowing probability in the EXNEX model. The extension also involves excluding baskets with sufficiently heterogeneous responses to be treated as independent. In comparison to the EXNEX model, this method increases the sensitivity to the level of similarity between responses in order to borrow between homogeneous baskets with higher probabilities, whilst reducing the chance of borrowing from baskets with heterogeneous response rates in order to control the type I error rate to an appropriate level. We show that this proposed extension has the ability to increase power and precision of estimates compared to an independent/stratified analysis whilst controlling the type I error rate in some scenarios or performing similarly to the standard EXNEX model in others.

Although it may be clear that the performance of said information borrowing methods will depend on the homogeneity of the data, with methods that borrow information across all baskets outperforming those which borrow to a lesser extent in cases of homogeneity in response rates (and vice‐versa under cases of heterogeneity), it is less clear the impact this will have on certain operating characteristics such as error control. It is also a challenge to quantify the “strength” of borrowing. The focus on this article is to monitor how certain metrics (primarily the type I error rate) are affected based on method used and homogeneity/heterogeneity of response data. This is explored through thorough simulation studies.

This article will be outlined as follows. In Section 1.1, we will introduce the setting of a motivating trial, the VE‐BASKET study, that forms a basis for the comparison setting. In Section 2, we describe information borrowing models and propose the extension to the exchangeability‐nonexchangeability model. In Section 3, we conduct a simulation study and then re‐analyze the results of the VE‐BASKET study using borrowing methods in Section 4.

### 1.1 Motivating trial: VE‐BASKET study

**FIGURE 1:**
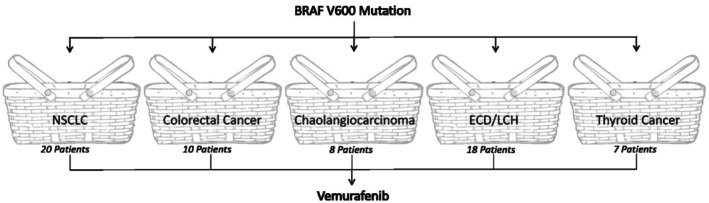
VE‐BASKET trial design.

This article is motivated by the VE‐BASKET trial^11^ which explored the effect of Vemurafenib on multiple cancer types with the BRAFV600 mutation. From 2012 to 2014, 63 patients with the BRAFV600 mutation were enrolled and divided into baskets based on cancer types. The baskets included were non‐small‐cell lung cancer (NSCLC), Erdheim‐Chester disease (ECD)/Langerhans'‐cell histiocytosis (LCH), cholangiocarcinoma, colorectal cancer, anaplastic thyroid cancer and an “all‐other” group consisting of patients of different disease types with the BRAF V600 mutation. For the purpose of this work, baskets were only considered if they received the same treatment (Vemurafenib), with the same tumor criterion (solid tumor types) and thus the “all‐other” basket was excluded. The arms of the trial are summarized in Figure 1.

The primary endpoint of this study was the overall response rate (ORR) with a null response rate of 15% indicating inactivity. The target response rate was 45% while a response of 35% was considered low but still indicative of a response. For a stratified analysis of baskets, the planned sample size, obtained through a Simon's two‐stage design,^12^ was 13 per basket based on 80% power and 10% type I error rate. However, different sample sizes were realized with the thyroid cancer basket, for example, consisting of just seven patients. This limited sample size causes issues when drawing inference from trial results as estimation of treatment effects will lack precision and thus any conclusions made regarding the effect of Vemurafenib on thyroid cancer may be questionable. However, due to baskets sharing a common genetic aberration one can utilize information borrowing techniques.

## 2 METHODS

### 2.1 Setting

Consider a basket trial consisting of K baskets. This article focuses on a single treatment arm setting and a primary binary endpoint, in which a patient either responds positively to a treatment or does not. Denote the responses in basket k (k=1,…,K) by $Y_{k}$, which follows a binomial distribution, $Y_{k}∼\mathrm{Bin}(n_{k},p_{k})$, with $n_{k}$ and $p_{k}$ indicating the sample size and response rate in basket k respectively. Interest lies in estimating the unknown response rate, $p_{k}$. Denote $q_{0}$ as the null response rate which indicates inactivity and $q_{1}$ as the target response rate. The objective is to test the family of hypotheses:

$$H_{0}\;:\;p_{k}\leq q_{0}vsH_{a}\;:\;p_{k}>q_{0},k=1,\ldots ,K.$$

To test these hypotheses a Bayesian framework is used. Having observed data D, at the conclusion of the trial the treatment is deemed effective in basket k if $\mathbb{P}(p_{k}>q_{0}|D)>\Delta_{\alpha }$.

The decision cut‐off, $\Delta_{\alpha }$, is typically calibrated under a null scenario in which the treatment effect is homogeneous and ineffective across baskets, to control error rates at a nominal level, α. This article utilizes calibration in order to control a basket specific type I error at the nominal level under a null scenario, however, as an alternative approach Psioda et al^10^ instead calibrated to control the family‐wise error rate across all baskets in the trial. Despite this calibration, methods that borrow information from heterogeneous baskets are expected to have error rates greater than α. Borrowing causes a shift in the the posterior density of $p_{k}$ towards a common mean and thus, when borrowing from a basket with a larger heterogeneous response, the point estimate obtained tends to increase, as does the probability $\mathbb{P}(p_{k}>q_{0}|D)$, so more baskets are erroneously deemed sensitive to treatment. When no borrowing occurs this shift is not present as the level of heterogeneity is irrelevant, so the same inflation is not expected.

### 2.2 Independent model

Independent analysis is an approach that does not borrow information between baskets and instead conducts stratified analysis for each. As such, for each basket, only data observed from its set of patients is considered when estimating its treatment effect. For each basket k in 1,…,K

(1) $$\begin{array}{ll} & Y_{k}\;∼\;\text{Binomial}(n_{k},p_{k}),\\ & \theta_{k}=\log(\frac{p_{k}}{1-p_{k}}),\\ & \theta_{k}\;∼\;N(\text{logit}(q_{0k}),\sigma_{k}^{2}), \end{array}$$

where $q_{0k}$ denotes the null response rate in basket k. The logit transformation of the response rates is taken to avoid boundary issues when $p_{k}$ is close to 0 or 1 and to align with the borrowing models to allow for a fair comparison. A slightly informative normal prior is placed on this transformed parameter, with mean based on the null response rate but with a large variance, $\sigma_{k}^{2}$. This method controls the type I error rate as the response rates do not depend on the level of heterogeneity across baskets, but estimates lack statistical power and suffer lower precision when a basket has a small sample size.^13^

### 2.3 Bayesian hierarchical model

The BHM, proposed by Berry et al,^7^ utilizes the full exchangeability assumption as all baskets share a common genetic change. With this assumption in mind, each basket's response to a treatment can be expected to be homogeneous and thus information can be shared between all baskets in the trial. The BHM is specified such that the log‐odds of the response rate for each basket follows a normal distribution, centered around a common mean μ with variance $\sigma^{2}$. Hyper‐priors are placed on the parameters μ and $\sigma^{2}$.

(2) $$\begin{array}{ll} & Y_{k}\;∼\;\text{Binomial}(n_{k},p_{k}),k=1,\ldots ,K\\ & \theta_{k}=\log(\frac{p_{k}}{1-p_{k}})\;∼\;N(\mu ,\sigma^{2}),\\ & \mu \;∼\;N(\text{logit}(q_{0}),\nu_{\mu }),\;\sigma \;∼\;g(\cdot ). \end{array}$$

The hyper‐prior on μ is suggested to be slightly informative^7^ based on the average null response rate across the baskets, with a large variance. The choice of hyper‐prior on σ, g(·), is widely debated with inverse‐gamma, half‐normal, or half‐Cauchy densities commonly used. An inverse‐gamma prior on $\sigma^{2}$ was utilized in the original paper,^7^ however, as stated by Gelman,^14^ this has poor behavior when $\sigma^{2}$ is close to 0 and thus a half‐Cauchy prior on σ with a moderately large scale was suggested instead.

Under the BHM, borrowing occurs between all baskets and as a result, the estimates of the response rates for each basket are shrunk towards the common mean with the degree of shrinkage controlled by the so called shrinkage/borrowing parameter, $\sigma^{2}$. When $\sigma^{2}$ tends to 0, borrowing moves towards the complete pooling approach in which the results of all baskets are combined and inference is made based on a single response rate. At the other extreme, when $\sigma^{2}$ tends to infinity, inference is akin to an independent analysis. This pull towards the common mean can result in a basket's treatment effect estimate being pulled away from the true value, particularly in the presence of a heterogeneous basket.

### 2.4 Calibrated Bayesian hierarchical model

The CBHM, proposed by Chu and Yuan,^8^ is an extension of the BHM and as such also makes the full exchangeability assumption. The CBHM has the same form as model (2), but rather than placing a prior on σ directly, it is defined as a function of a measure of homogeneity across baskets: $\sigma^{2}=\exp\{a+b\log(T)\}$, where T is the chi‐squared test statistic for homogeneity:

(3) $$T=\overset{K}{\underset{k=1}{\sum }}\frac{{\left(O_{0k}-E_{0k}\right)}^{2}}{E_{0k}}+\overset{K}{\underset{k=1}{\sum }}\frac{{\left(O_{1k}-E_{1k}\right)}^{2}}{E_{1k}},$$

where $O_{0k}$ and $O_{1k}$ are the observed failures and responses in basket k respectively, while $E_{0k}$ and $E_{1k}$ are the expected failures and responses in basket k.

The parameters a and b are tuned to calibrate the function to ensure strong borrowing through hierarchical modeling when all baskets have a homogeneous response and treat baskets as independent otherwise. The calibration procedure is outlined by Chu and Yuan^8^ as follows:

1. Generate R simulated data sets in which the treatment is effective in all baskets' with response rate $q_{1}$, for each computing T as in (3). Let $H_{B}$ be the median of these T values.
2. Simulate the case in which the treatment effect is heterogeneous across baskets. To do so, let $q(j)=(q_{1},\ldots ,q_{1},q_{0},\ldots ,q_{0})$ be the scenario in which the treatment is effective in the first j baskets but not effective in baskets j+1 to K. For each value of j∈{1,…,K−1} generate R simulations of data, calculating the test statistic T for each. Denote $H_{\bar{B}j}$ as the median value of T for each value of j. Finally, define $H_{\bar{B}}=\min_{j}(H_{\bar{B}j})$.
3. Let $\sigma_{B}^{2}=1$ under which strong borrowing occurs and $\sigma_{\bar{B}}^{2}=80$ under which little to no information borrowing takes place. Noting that $\sigma^{2}=g(T)=\exp\{a+b\log(T)\}$, solve a and b for $\sigma_{B}^{2}=g(H_{B})$ and $\sigma_{\bar{B}}^{2}=g(H_{\bar{B}})$. This results in:
$$a=\log(\sigma_{B}^{2})-\frac{\log(\sigma_{\bar{B}}^{2})-\log(\sigma_{B}^{2})}{\log(H_{\bar{B}})-\log(H_{B})}\log(H_{B}),\;\;b=\frac{\log(\sigma_{\bar{B}}^{2})-\log(\sigma_{B}^{2})}{\log(H_{\bar{B}})-\log(H_{B})}.$$

A benefit of such a tuning procedure is the increased certainty in estimates produced by the CBHM in comparison to the BHM in the case where all baskets are homogeneous. However, with a and b tuned in this way, the method takes on a ‘strong’ definition of heterogeneity such that if the response rate in one basket is heterogeneous, then all baskets are deemed heterogeneous, and as a result no borrowing occurs. The “strong” definition of heterogeneity can be relaxed through a less stringent tuning procedure but this comes at the cost of the error control.

The original calibration procedure for the CBHM, proposed by Chu and Yuan,^8^ was based on equal sample sizes for each basket. In practice it is unlikely that all baskets will recruit exactly the same number of patients, so the calibration outlined above may not be adequate. When the sample sizes differ, Step 2 in the calibration does not cover all possibilities of heterogeneity as the ordering of response rates matter. We propose altering this step for unequal sample sizes to consider all permutations of $q_{1}$ and $q_{0}$ in which at least one basket has response rate $q_{0}$ and at least one has response rate $q_{1}$.

### 2.5 Exchangeability‐nonexchangeability model

The full exchangeability assumption is often violated in the presence of heterogeneous baskets. The exchangeability‐nonexchangeability (EXNEX) model, proposed by Neuenschwander et al,^9^ incorporates a nonexchangeability component to the standard BHM, within which no borrowing occurs. The model then has two components:

1. EX (exchangeable component): with prior probability $\pi_{k}$, basket k is exchangeable and a BHM as in model (2) is applied. Information borrowing is therefore conducted between all baskets assigned to the exchangeable component.
2. NEX (nonexchangeable component): with prior probability $1-\pi_{k}$, $\theta_{k}$ is nonexchangeable with any other basket, and as a result, basket k is treated independently.

(4) $$\begin{array}{ll} Y_{k}∼\text{Binomial}(n_{k},p_{k}),\; & M_{1k}∼N(\mu ,\sigma^{2}),\text{(EX)}\\ \theta_{k}=\log(\frac{p_{k}}{1-p_{k}}),\; & \mu ∼N(\text{logit}(q_{0}),\nu_{\mu }),\\ \theta_{k}=\delta_{k}M_{1k}+(1-\delta_{k})M_{2k},\; & \sigma ∼g(\cdot ),\\ \delta_{k}∼\text{Bernoulli}(\pi_{k}),\; & M_{2k}∼N(m_{k},\nu_{k}).\text{(NEX)}. \end{array}$$

As information is borrowed only between baskets assigned to the EX component but not from those in the NEX component, this model provides more flexibility compared to the previous methods as information can be borrowed between just some of the baskets and not all of them.

Careful consideration is needed in this model when it comes to the selection of $\pi_{k}$ values. It is uncommon to have strong prior information on the probability of exchangeability, so it is suggested to fix these prior to the trial at $\pi_{k}=0.5$ for all baskets. This prior probability is updated to a some degree based on the homogeneity of the data but is not sensitive enough to the heterogeneity/homogeneity of responses and thus it is anticipated that the probability of borrowing from a heterogeneous basket will be too high, which in turn will inflate the type I error rate. Ideally the prior probability of assigning homogeneous baskets to the exchangeability component should increase, while those for heterogeneous baskets decreases as opposed to fixing these probabilities at 0.5 each.

Note that a Dirichlet prior could be placed on $\pi_{k}$, however, as stated by Neuenschwander et al,^9^ this does not have a substantial effect on inference in comparison to fixing the weights a priori. The EXNEX model can also be easily extended to have more than one exchangeability component, allowing us to borrow between different subsets of baskets.

### 2.6 Proposed modified EXNEX model

In the original EXNEX model, the prior probability values, $\pi_{k}$, do not dependent on the similarity of the data. We propose a modification to the EXNEX model, denoted ${\text{mEXNEX}}_{c}$, which sets these $\pi_{k}$ values to account for the homogeneity of the response in basket k compared to that in all other baskets. A similar concept of updating prior weights based on homogeneity of responses was proposed by Haiyan and Hampson^15^ but in the dose‐finding setting. The purpose of this is to increase the sensitivity to the heterogeneity of response data compared to the EXNEX model.

The Hellinger distance is an ideal metric that quantifies the similarity between two probability distributions parameterized by probability density functions. In the ${\text{mEXNEX}}_{c}$ model it is used to compare the distance in responses between baskets. The Hellinger distance gives values on the [0,1] range, equating to 0 when densities are identical and increasing values as the distance between the densities becomes greater and as such, they can be easily translated into probability values.

The ${\text{mEXNEX}}_{c}$ model is a two‐step procedure, the first step removes baskets with a clearly heterogeneous response rate. A pre‐specified cut‐off value, c, is chosen to indicate that a basket is sufficiently heterogeneous to exclude from borrowing and treat as independent. Denote ${\hat{p}}_{k}=Y_{k}/n_{k}$. If the minimum pairwise difference in response rate between basket k and all other baskets is greater than c,

$$\underset{k^{\prime }}{\min}\{|{\hat{p}}_{k}-{\hat{p}}_{k^{\prime }}|\}>c,\;\;\;\;k\neq k^{\prime },$$

then basket k is treated as independent and its mixture weight, $\pi_{k}$, in the EXNEX model is set to 0.

In the second step, denote S as the set of all baskets not excluded for heterogeneity. For all baskets in S, produce posterior densities for $p_{k}$ by fitting a beta‐binomial model with prior $p_{k}∼\text{Beta}(1,1)$, which has form $p_{k}|Y_{k}∼\text{Beta}(a_{k},b_{k})$ where $a_{k}=Y_{k}+1$ and $b_{k}=n_{k}-Y_{k}+1$. The Hellinger distance between posteriors of basket k and $k^{\prime }$ is computed as

(5) $$h_{k,k^{\prime }}=\sqrt{1-\frac{B(\frac{a_{k}+a_{k^{\prime }}}{2},\frac{b_{k}+b_{k^{\prime }}}{2})}{\sqrt{B(a_{k},b_{k})B(a_{k^{\prime }},b_{k^{\prime }})}}},\;\;\;(k,k^{\prime }\in S),$$

where B(·,·) is the Beta function. The probability, $\pi_{k}$, is then calculated as

$$\pi_{k}=\underset{k^{\prime }}{\sum }\frac{1-h_{k,k^{\prime }}}{|S|-1}\;\;\text{for}\;\;k,k^{\prime }\in S,\;\;k\neq k^{\prime }.$$

Once obtained, these $\pi_{k}$ values are used as the prior borrowing probabilities in model (4). For the ${\text{mEXNEX}}_{c}$ model, a slight alteration is made to model (4), in that, a prior is placed on $\sigma^{2}$ as opposed to σ in order to have less mass concentrated around 0.

This method is expected to reduce the probability of heterogeneous baskets being assigned to the EX component as a heterogeneous basket will have larger Hellinger distances and thus lower $\pi_{k}$ values. As such, the ${\text{mEXNEX}}_{c}$ model is expected to possess better error control than the standard EXNEX model that assigns fixed $\pi_{k}$ values irrespective of the homogeneity of responses.

The specification of the cut‐off c to define a basket as sufficiently heterogeneous to remove requires careful consideration. When defining c prior to the trial, the clinician must weigh up the trade‐off between achieving higher power of estimates while maintaining an adequate error rate. A larger c value will result in higher power at the cost of inflation of error rates, whilst lower, more conservative values control error rates but provide a smaller increase in power. A cut‐off is chosen such that this trade‐off is considered acceptable.

A proposed method for this specification is through a pre‐trial simulation study in which the null and target response rate and planned samples sizes are used to compute operating characteristics for different values of c, with $\Delta_{\alpha }$ re‐calibrated for each. The planned sample sizes are obtained as in the trial protocol, using a Simon two‐stage design based on stratified analysis on each basket for a targeted type I error rate and power. Generally, consider cut‐off values of $c=i/\max n_{k}$ for $i=0,1,2,\ldots ,n_{k}$ and k=1,…,K and scenarios that cover all combination of insensitive and sensitive baskets. To guide the selection of c, it is chosen such that:

(6) $$c=\arg\underset{c}{\max}\{x{\text{Power}}_{c}+(1-x)(1-{\text{Error}}_{c})\},\;x\in [0,1],$$

where Power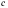 and Error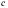 are the mean power and type I error rate for cut‐off c across all considered scenarios. The value of x is chosen to balance the trade‐off between power and error‐rate control.

### 2.7 Bayesian model averaging

Psioda et al^10^ proposed a BMA approach that allows for both exchangeability and nonexchangeability, but in place of applying a single model to the data, the average over all considered models is taken. To do so one averages over the posterior distribution under each model, weighted by their posterior model probability.^16^

Consider the case where only a single exchangeability component is allowed. Define ${ℳ}_{j}$ as model j representing a permutation of basket allocation to the EX group or NEX group. Rather than applying a hierarchical model to borrow between baskets in the EX group, results are pooled and baskets have one shared response rate $p_{S_{i,j}}$, where $S_{i,j}$ is a subset i of the baskets' given model ${ℳ}_{j}$. Therefore, $p_{k}=p_{S_{i,j}}$ when $k\in S_{i,j}$.

A weakly informative Beta prior is placed on the response rates, while a prior on each model, $f({ℳ}_{j})$, is also required. The posteriors $f(p_{k}|{ℳ}_{j})$ and $f({ℳ}_{j}|D)$ are computed after observing response data D and are used to implement a BMA procedure to obtain the efficacy decision for basket k at the conclusion of a trial by computing $\mathbb{P}(p_{k}>x|D)=\sum_{j}\mathbb{P}(p_{k}>x|{ℳ}_{j},D)f({ℳ}_{j}|D)$.

This method is potentially advantageous as it accounts for all possible borrowing subsets in place of applying a single model. This allows for uncertainty in the model selection, as the specification of an incorrect model may lead to misleading inference. Also, as a result of pooling within exchangeability groups, closed‐form solutions of posteriors can be found. This is computationally appealing as it can be implemented quickly even for a large number of baskets.

## 3 SIMULATION STUDY

In order to assess the performance of the described methods in terms of estimation, type I error and power, a simulation study was conducted. Motivated by the VE‐BASKET trial, the conducted simulation study consists of five baskets. Two settings are considered:

(i) Sample size in each basket being equal to the planned sample size of 13 patients,
(ii) Sample sizes in the baskets being the realized sample sizes in the trial (ie, 20, 10, 8, 18, and 7).

Set $q_{0}=0.15$ and $q_{1}=0.45$ as the null and target response rates respectively. A basket is deemed sensitive to a treatment at the conclusion of a trial, having observed data D, if $\mathbb{P}(p_{k}\geq 0.15|D)>\Delta_{\alpha }$, where $\Delta_{\alpha }$ is calibrated to obtain a type I error rate of α=10% under the null scenario. Note that $\Delta_{\alpha }$ is calibrated for each method separately and follows the same procedure for both the proposed and existing methods—for the ${\text{mEXNEX}}_{c}$ model, c is selected through calibration but is then taken as fixed when calibrating $\Delta_{\alpha }$. This is done based on the planned sample size $n_{k}=13$ for all baskets k and the null response rate $q_{0}=0.15$. The calibrated $\Delta_{\alpha }$ values for each method are given in Table S1 of the supplementary material.

Several scenarios with varying numbers of baskets sensitive to treatment are considered and displayed in Table 1. Scenario 1 is the null case in which all baskets are insensitive. Scenarios 2‐5 cover different combinations of insensitive and sensitive treatment baskets while Scenario 6 is the case where all baskets are homogeneous and sensitive. This will highlight the benefits, if any, the borrowing methods provide in terms of power improvement. Scenarios 7‐10 consist of cases where some baskets have a marginally effective response rate at 35%. For the realized sample size case, a further six data scenarios are considered to account for the fact that ordering of response rate now matters.

**TABLE 1:** True response rate data scenarios: For the planned sample size simulation Scenarios 1‐10 are considered, whereas, for the realized sample size simulation all Scenarios 1‐16 are considered.

	$p_{1}$	$p_{2}$	$p_{3}$	$p_{4}$	$p_{5}$
Scenario 1	0.15	0.15	0.15	0.15	0.15
Scenario 2	0.45	0.15	0.15	0.15	0.15
Scenario 3	0.45	0.45	0.15	0.15	0.15
Scenario 4	0.45	0.45	0.45	0.15	0.15
Scenario 5	0.45	0.45	0.45	0.45	0.15
Scenario 6	0.45	0.45	0.45	0.45	0.45
Scenario 7	0.35	0.15	0.15	0.15	0.15
Scenario 8	0.35	0.35	0.35	0.15	0.15
Scenario 9	0.45	0.35	0.35	0.15	0.15
Scenario 10	0.45	0.45	0.35	0.35	0.15
Scenario 11	0.15	0.15	0.15	0.15	0.45
Scenario 12	0.15	0.15	0.45	0.15	0.45
Scenario 13	0.15	0.45	0.45	0.15	0.45
Scenario 14	0.15	0.45	0.45	0.45	0.45
Scenario 15	0.45	0.15	0.15	0.15	0.45
Scenario 16	0.45	0.15	0.45	0.15	0.45

For each method and scenario the following operating characteristics are computed:

- % Reject: the percentage of simulated data sets in which the null hypothesis is rejected. If the null is true then this value is the type I error rate, else it is the power.
- % All correct: the percentage of simulated data sets in which the correct conclusions are made across all baskets.
- FWER (family‐wise error rate): the percentage of simulated data sets in which at least one null basket is deemed sensitive to treatment.
- Mean point estimate of the response rate in each basket and the standard deviation of said estimate across the simulations.

The results presented focus on the first three of these, with results for the mean point estimates provided in Section 3 of the supplementary material.

For the following analysis, prior and parameter choices for each model are summarized in Table 2 with full model specification provided in Appendix A. Priors on μ are centered around the null response rate of 0.15 with a large variance. Priors on $\sigma^{2}$ are chosen to be consistent with those used in the literature. The EXNEX model has prior borrowing probabilities fixed at 0.5. The prior parameters for the mEXNEXc model are kept the same as the standard EXNEX model to allow for fairer comparison. These parameters are selected by the recommendation of Neuenschwander et al,^9^ with the prior for the NEX component in both the EXNEX and the ${\text{mEXNEX}}_{c}$ model centered around a plausible guess of $p_{k}$ of 0.35. The priors for the BMA are consistent with those suggested by Psioda et al^10^ with priors placed on each model being the number of distinct response rates in that model squared.

**TABLE 2:** Model prior and parameter specification for the simulation study.

Model	Prior and parameter specification
Independent	$\theta_{k}∼N(\text{logit}(0.15),10^{2})$
BHM	$\mu ∼N(\text{logit}(0.15),10^{2})$, σ∼Half‐Cauchy(0,25)
CBHM	$\mu ∼N(\text{logit}(0.15),10^{2})$, $\sigma^{2}=\exp\{-7.25+5.86\log(T)\}$
EXNEX	$\mu ∼N(\text{logit}(0.15),10^{2})$, σ∼Half‐Normal(0,1), $M_{2k}∼N(-0.62,4.4^{2})$, $\delta_{k}∼\text{Bernoulli}(0.5)$
${\text{mEXNEX}}_{c}$	$\mu ∼N(\text{logit}(0.15),10^{2})$, $\sigma^{2}∼\text{Half‐Normal}(0,1)$, $M_{2k}∼N(-0.62,4.4^{2})$, $\delta_{k}∼\text{Bernoulli}(\pi_{k})$
BMA	$P_{S_{j}}|{ℳ}_{j}∼\text{Beta}(0.45,0.55)$, $f({ℳ}_{j})∼P_{j}^{2}$

**FIGURE 2:**
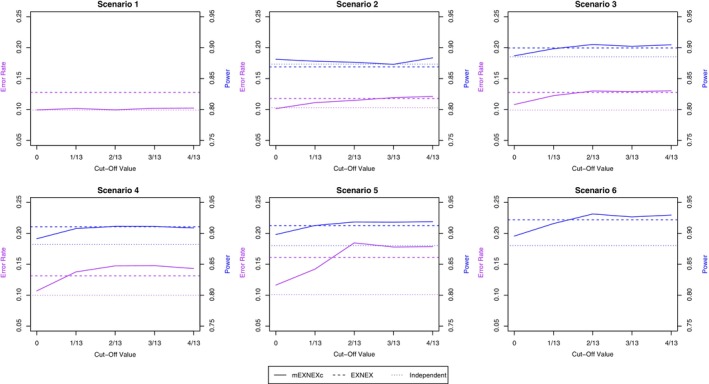
Pre‐trial simulation results for type I error rate and power across the data scenarios for different cut‐off values, c.

The specification of the cut‐off value, c, in the ${\text{mEXNEX}}_{c}$ model is chosen through a pre‐trial simulation as outlined in Section 2.6. Cut‐off values of c=i/13 were considered where i=0,1,2,3,4. For each value of c, 10 000 simulated data sets were used to compute the type I error rate and power across the six scenarios in Table 1, with the results shown in Figure 2. Within Equation (6) two cases were considered: when x=0.4 a higher emphasis is placed on error control over power improvement resulting in the choice c=0. This is a more conservative value as it only allows for borrowing when a basket has an identical response rate to at least one other basket. However, despite this conservative nature, from Figure 2, we observe that this specification shows control of the type I error rate close to the nominal 10% level under Scenarios 1‐4, whilst improving power under Scenarios 2, 4‐6 in comparison to an independent model. Denote this model as ${\text{mEXNEX}}_{0}$. The second choice is x=0.6 which puts greater weight on power improvement whilst relaxing the degree of error control, resulting in the choice c=1/13. Denote this model as ${\text{mEXNEX}}_{1/13}$. A total of 10 000 simulations were run using the “rjags” package v 4.12,^17^ within RStudio v 1.1.453^18^ for each of the six data scenarios in Table 1.

### 3.1 Simulation results: Planned sample sizes

**FIGURE 3:**
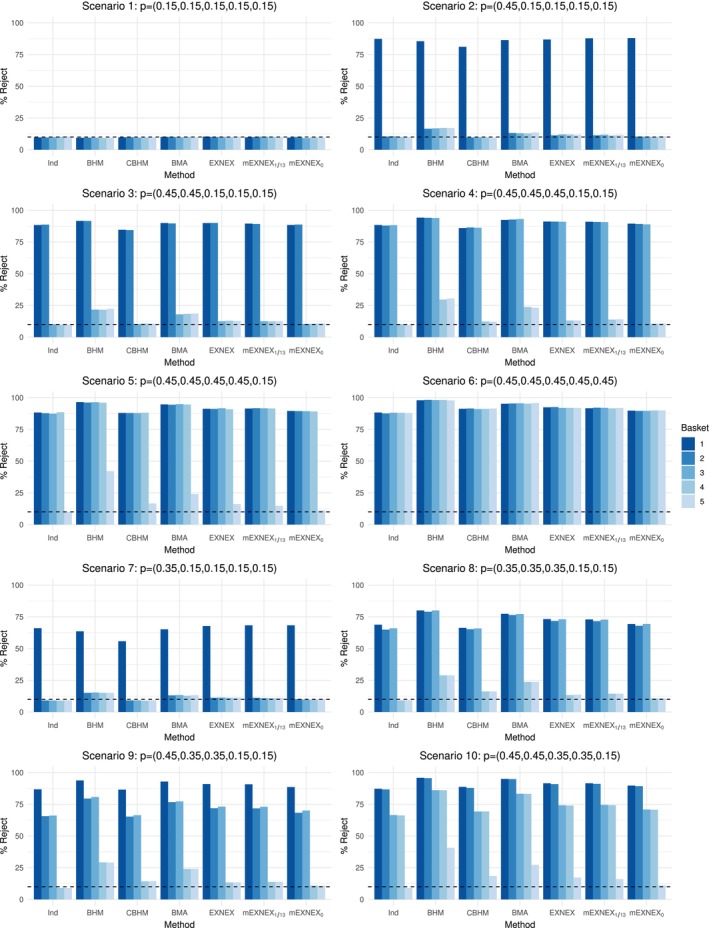
Percentage of rejections of the null hypothesis for each method and data scenario based on a planned sample size of 13 patients per basket.

The results for power and type I error rate under the planned sample size are presented in Figure 3 which shows the percentage of simulated data sets in which the null hypothesis was rejected for each method and scenario. Full results are also provided in Table B1 and B2 in Appendix B.

The rejection percentages are calibrated under Scenario 1 to achieve a 10% type I error rate for each method separately and hence all rejections are approximately 10%. However, in the presence of a single heterogeneous effective basket, that is, Scenario 2, the ${\text{mEXNEX}}_{0}$ model gives the best performance with error control at 10% whilst achieving the greatest power (88%). The CBHM also controls the type I error rate but only achieves 81.1% power due to the level of heterogeneity and the nature of the calibration procedure. The BHM, BMA, and EXNEX model all have raised error rates at approximately 16.9%, 13.2%, and 11.8% respectively. The ${\text{mEXNEX}}_{1/13}$ model gives power that is increased by 0.8 compared to the EXNEX model with a slightly lower type I error rate of 11.5%.

Across Scenarios 3‐5 there is a mix of sensitive and insensitive treatment baskets. These scenarios show the benefits in terms of power gain through information borrowing techniques compared to an independent analysis. Again in these cases the CBHM lacks power due to the heterogeneity of the data, giving consistently lower power than an independent analysis, whilst the BHM and BMA procedure give hugely inflated error rates. The BHM gives type I error rates ranging from 21.6% to 42.1% across these three scenarios.

The ${\text{mEXNEX}}_{1/13}$ model leads to similar results to the standard EXNEX model in these scenarios due to its inability to detect clusters of responses, which leads to increased probability values for EX assignment for all baskets. In Scenario 4 this results in a greater type I error rate for the ${\text{mEXNEX}}_{1/13}$ compared to the EXNEX model (15% vs 13.1%), however, under Scenario 5 the ${\text{mEXNEX}}_{1/13}$ model gives a 1.3 decrease in the type I error rate. Under a more conservative cut‐off, the ${\text{mEXNEX}}_{0}$ model keeps the type I error rate at an acceptable level with the worst case occurring under Scenario 5 in which the error rate is just 11.2%, which is much lower than the 16.1% error rate of the EXNEX model. This is all whilst also increasing power over an independent model by 1.4%.

In Scenario 6, when all baskets are sensitive to treatment, the BHM followed by the BMA procedure give the greatest power at the cost of inflated error rates across the other scenarios. The ${\text{mEXNEX}}_{1/13}$ model has similar power to the standard EXNEX model with mean power 91.9% compared to 92.2%, whereas the ${\text{mEXNEX}}_{0}$ model, has lower average power at 89.8% but still an improvement over the independent model at 88.0%.

Now consider the cases where some baskets are marginally effective with a true response rate of 35%. In particular one can draw comparisons between Scenarios 2 and 7 as in both cases just a single basket is heterogeneous and effective to some degree. Under both scenarios the same patterns of results are observed, but due to the lower true response rate under Scenario 7, the difference in power and error rates between methods has been amplified. As expected, the error rates tend to be lower under Scenario 7 compared to 2, as the pull upwards towards the heterogeneous basket will be less extreme as it has a true response rate closer to that under the null. In this case, both ${\text{mEXNEX}}_{c}$ models give the joint highest power at 68.3%, with the ${\text{mEXNEX}}_{0}$ model again controlling error rates at the 10% level, while only minimal inflation is observed under the ${\text{mEXNEX}}_{1/13}$ model at 11.1% (a value very similar to that of the standard EXNEX model). The BHM, CBHM, and BMA approach all give lower power than an independent analysis with clearly inflated error rates in the BHM and BMA cases. Similar connections can be made between Scenarios 4 and 8, with the same conclusions drawn from each.

Under both Scenarios 9 and 10, baskets have a combination of effective, marginally effective and ineffective response rates. Predictably, the BHM and BMA approach give the greatest power but with this have inflated error rates, just as in Scenarios 4 and 5. All of these Scenarios 7‐10 demonstrate the ability of the ${\text{mEXNEX}}_{c}$ model to control error rates when c=0 whilst improving power over an independent analysis anywhere from 1.8% to 3% for effective baskets and 3.3%‐4.5% for marginally effective baskets.

Looking now at the percentage of data sets in which the correct inference was made across all baskets, alongside the family‐wise error rates (where $\Delta_{\alpha }$ was now calibrated under Scenario 1 to achieve 25% FWER—full results provided in Section 1 of the supplementary material). All methods gave similar values for correct inference under the null scenario. However, under both Scenarios 2 and 7, the independent model produced the greatest values, closely followed by the ${\text{mEXNEX}}_{c}$ models. Across Scenarios 3‐6 both metrics simultaneously decrease for the independent model, and also demonstrates lowest percentage of correct inference compared to all other methods in Scenarios 8‐10. The ${\text{mEXNEX}}_{1/13}$ model has similar or lower percentage of correct inference in comparison to the EXNEX model but with consistently lower FWER values, while the ${\text{mEXNEX}}_{0}$ method has greater proportions of correct inference in Scenario 3 compared to the standard EXNEX model (54.0% compared to 51.8%) but a 14% decrease under Scenario 5. This reduction came with a 3.3% decrease in FWER. Under Scenario 6, the methods shown to have higher power in Figure 3, also gave greater proportion of correct inference made across all baskets. Considering Scenarios 8‐10, the standard EXNEX model gives the best percentage of all correct inference with lower FWER than the BHM, CBHM and BMA approach in all cases. This is most prominent in Scenario 9 where in 37.1% of simulation, the EXNEX model made correct conclusions in all five baskets, whereas, under the same scenario the ${\text{mEXNEX}}_{1/13}$ had a smaller value at 30.2% but with a 2% lower FWER.

In view of these results, when the sample size is fixed across baskets, the proposed ${\text{mEXNEX}}_{0}$ model controls error rates to a nominal level whilst also improving power over implementing an independent model. Improvements are also observed over the EXNEX model with consistently lower type I error rates but reduced power. Should interest lie more heavily on improving power over the control of error rates, the cut‐off value for exclusion of heterogeneous baskets could be increased. Both cut‐off values of 0 and 1/13 produce a model that either exceeds all other considered borrowing methods in performance or acts similarly to the standard EXNEX model.

#### 3.1.1 Varying the true response rate vector, p

There are an infinite number of data scenarios one could fall in when conducting clinical trial analysis, the scenarios listed in Table 1 are only a subset of these feasible cases. The data scenarios implemented above were selected to cover a wide range of cases, however, some important cases may not have been investigated.

To overcome this, a further simulation study was conducted within which, rather than fixing the true probability of success parameter prior to the study, forever simulation run a new random truth vector, p, was generated with uniform probability across the ranges [0,0.15] and [0.35,0.5] (these ranges were set to ensure equal changes of lying in the null and non‐null case respectively). Once p was generated, it was used to simulate data from a binomial distribution. The goal of such a simulation study is to determine the operating characteristics on average over many different truth vectors in hope to capture what would occur in cases not investigated within the previous simulation study.

A total of 20 000 simulations for each borrowing method were run under the planned sample size case of 13 patients in each basket. Results are provided in Table 3, with further descriptions and results for the realized sample size case provided in Section 4 of the supplementary material.

**TABLE 3:** Operating characteristics for the planned sample size simulation study in which the truth vector was randomly generated.

Method	Type I error rate	Power	% All correct	FWER
Independent	2.25	81.48	57.63	5.68
BHM	5.00	87.51	63.07	11.68
CBHM	2.27	79.21	54.21	5.48
BMA	4.51	86.87	62.57	10.62
EXNEX	3.15	86.01	63.64	7.86
${\text{mEXNEX}}_{0}$	2.68	83.89	61.19	6.77
${\text{mEXNEX}}_{1/13}$	3.16	85.97	63.78	7.80

Similar to the fixed scenario cases described above, the BHM and BMA have the highest error rates, but all methods have mean type I error rate less than the nominal 10% level. The reduced error rates come from, in some cases, the true response rate lying well below the null 15% level under which the $\Delta_{\alpha }$ value was calibrated. The CBHM continues to behave similarly to an independent approach but with lower power.

The standard EXNEX model and ${\text{mEXNEX}}_{1/13}$ model behave very similarly in this study, both with type I error rate of 3.2% and power of 86.0%. This is not unexpected, as like in the previous study, when clusters of responses are present, the less conservative ${\text{mEXNEX}}_{c}$ model begins to perform similarly to the standard EXNEX model due to it is inability to detect clusters of responses. When c=0, error rates are far closer to the independent model at 2.7% (2.3% under an independent analysis) with 83.9% power, which although lower than the standard EXNEX model, is an increase of 2.4% over an independent analysis.

In terms of percentage of simulation runs in which the correct conclusion was made in all five of the baskets, both the standard EXNEX and ${\text{mEXNEX}}_{1/13}$ models have the highest value of around 63.7%. The BHM and BMA approach have similar but slightly smaller values compared to both methods but have 2.8%‐3.9% increase in FWER. The ${\text{mEXNEX}}_{0}$ model gives both reduced percentage of all correct conclusions and FWER compared to all the aforementioned methods but does possess a 3.6% increase in all correct inference compared to an independent analysis.

To summarize, in the planned sample size case when the true response rate is varied, the BHM and BMA continue to display the most undesirable error rates whilst the independent analysis and CBHM lack power. The modified EXNEX model with c=1/13 performs almost identically to the standard EXNEX model. When a more conservative cut‐off value c=0 is implemented, error rates are reduced by 0.5% compared to the standard EXNEX model but with a 2.1% reduction in power (but still a 2.4% improvement over an independent analysis).

### 3.2 Simulation results: Realized sample sizes

Although the protocol planned for 13 patients per basket, 20, 10, 8, 18, and 7 patients were enrolled across the five baskets. The thresholds for efficacy, $\Delta_{\alpha }$, were calibrated based on the planned sample size of 13 per basket and was not re‐calibrated based on these observed sample sizes. Similarly, the cut‐off values c in the ${\text{mEXNEX}}_{c}$ model were not adjusted and were based on the planned equal sample size.

**FIGURE 4:**
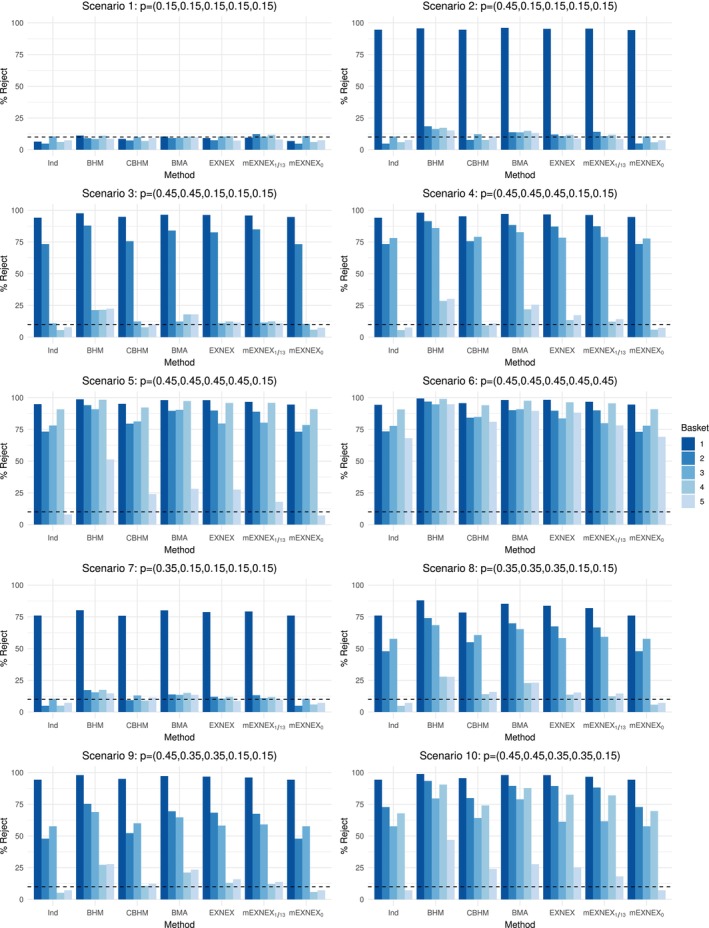
Percentage of rejections of the null hypothesis for each method under data Scenarios 1‐10 based on realized sample sizes of 20, 10, 8, 18, and 7 across the five baskets.

**FIGURE 5:**
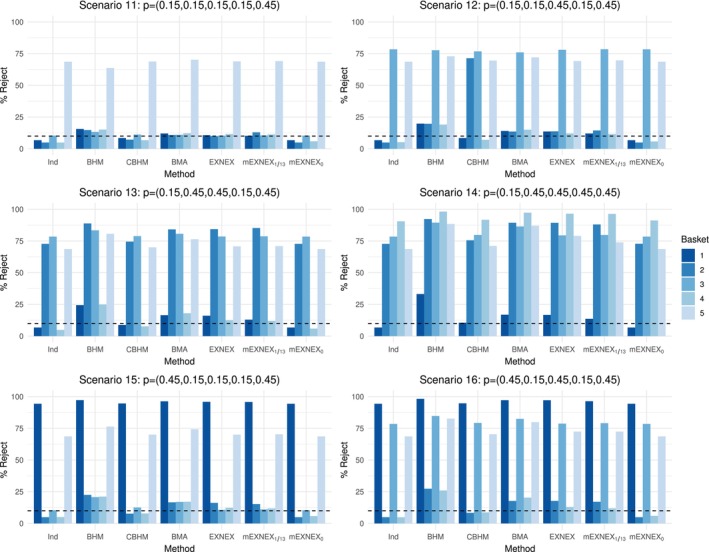
Percentage of rejections of the null hypothesis for each method and under data Scenarios 11‐16 based on realized sample sizes of 20, 10, 8, 18, and 7 across the five baskets.

Percentage of rejection plots are provided in Figures 4 and 5 with full results in Tables B3, B4, B5 of Appendix B.

The calibration procedure for the CBHM needs more careful consideration here, as the previous calibration was based on equal sample sizes across baskets. A slight modification to Step 2 of the process was made to cover all permutations of heterogeneity. Even with this adaption, when sample sizes are unequal, the calibrated values of a and b are much larger in magnitude than in the equal sample case. This leads to stronger borrowing where baskets are at least fairly homogeneous, producing much narrower posterior densities. These narrow posteriors, in some cases, have their mass lying entirely above $q_{0}$ and thus $\Delta_{\alpha }$ is close to 1. This can cause a lack of power as it makes it incredibly difficult to reject a hypothesis. To overcome this, we recommend calibrating a and b with the sample size fixed and equal for each basket at the averaged basket sample size. Analysis is then conducted using these tuned parameters with the observed unequal sample sizes. In this case, the average sample size across the baskets happens to be 13 patients per basket (with rounding) and thus, the a and b values used are the same as in the planned sample size case.

The type I error rate in Scenario 1 lies below the nominal 10% level for all methods with the independent and ${\text{mEXNEX}}_{0}$ models giving the lowest values, whilst the BHM, BMA, and EXNEX model are greater but are still approximately at or below 10%.

Under Scenario 2, in which the first basket is effective to treatment, the BHM, EXNEX, ${\text{mEXNEX}}_{1/13}$ and BMA methods produce higher power than the independent analysis, at the cost of inflated error rates at 18.4%, 14.8%, 12.0%, and 14.1% respectively. The CBHM and ${\text{mEXNEX}}_{0}$ model gives almost identical power values to the independent model (94.6% and 94.3% compared to 94.6%) but the ${\text{mEXNEX}}_{0}$ model gives error rates no greater than 10.5%. Similar results are also seen in Scenario 7 in which the first basket is marginally effective. Now consider Scenario 11 in which, like Scenario 2 only one basket has an effective response rate but this is now the fifth basket as opposed to the first. This basket has a smaller sample size than that of the first basket at just seven patients and thus, the power is uniformly lower for all methods, however, patterns of results remain the same in terms of method performance. Under this scenario, all methods (with the exception of the BHM) produce very similar power values ranging from 68.7% to 70.2% but all have varying error rates. All borrowing methods have a higher error rate than that of an independent model, with inflation above the nominal 10% level present under the BHM, BMA, EXNEX, and ${\text{mEXNEX}}_{1/13}$ model. The BHM has a much lower power in this case at 63.7%.

Across Scenarios 3, 4, and 8, the ${\text{mEXNEX}}_{1/13}$ model gives similar/lower error rates compared to the EXNEX model and generally higher power values in baskets with a small sample size, that is, baskets 2 and 3, with up to an increase of 2.4%. Similar power values are observed in basket 1 where the sample size is larger. The ${\text{mEXNEX}}_{0}$ model continues to control error rates at or below the 10% level but provides little to no improvement in terms of power over an independent approach. This is due to the conservative nature of this c value. When c=0, under unequal sample sizes it is likely that all baskets will be treated as independent, as in the binary response setting, achieving identical response rates in baskets of different sizes is often impossible.

Scenario 5 again displays the improvement in power through using the borrowing techniques, with the exception of the ${\text{mEXNEX}}_{0}$ model for the aforementioned conservative nature. Ignoring the independent and ${\text{mEXNEX}}_{0}$ models for lack of power, the ${\text{mEXNEX}}_{1/13}$ model displays the lowest type I error rate of 17.9% which, although inflated, is considerably lower than the other borrowing methods, including the EXNEX model which has an error rate of 27.6%.

Similar to the planned sample size simulation, the BHM and BMA approach give greatest power in Scenario 6 but at the cost of high error rates elsewhere. Across all baskets, the ${\text{mEXNEX}}_{1/13}$ model improves in power over the independent model by up to 16.51% but also at the cost of inflated error rates. However, this inflation occurs to a lesser extent than the EXNEX model across Scenarios 2‐5 with a maximum difference in error rates for the two methods at 9.7% which could be viewed as a highly significant margin.

Under Scenarios 9 and 10, those baskets that have a marginally effective treatment effect show markedly improved power when information borrowing methods are implemented, with the ${\text{mEXNEX}}_{1/13}$ model obtaining up to a 20% improvement in power compared to an independent analysis under Scenario 9—note this comes with roughly a 3% inflation in error rate, but such inflation is less than the other borrowing methods.

Now consider the cases when the ordering of response rates is altered, under Scenario 12 the two smallest baskets have the effective response rates whilst the larger baskets are insensitive to treatment, the ${\text{mEXNEX}}_{1/13}$ model gives the greatest power for basket 3 at 78.5% (whilst the EXNEX model has power 78.1%) as well as improved power over the standard EXNEX model for basket 5 also (69.7% compared to 69.2%). This is alongside having a lower average type I error rate of 12.7% under ${\text{mEXNEX}}_{1/13}$ compared to 13.1% under the EXNEX model. In comparison to Scenario 3, when the basket size is smaller, the performance of the BHM and a BMA approach worsens with higher errors and lower power values, whilst the performance of the ${\text{mEXNEX}}_{1/13}$ over other methods improves. The same conclusions can be drawn from Scenarios 13 to 16 also.

Considering family‐wise error rate and percentage of all correct conclusions across the five baskets, if the $\Delta_{\alpha }$ values were calibrated to control FWER at 25% under the planned sample size and then applied to the realized sample size case, all methods give slightly inflated FWER values of over 25% under Scenario 1 (see supplementary material for full results). There is a 1‐1 relation between low FWER and high percentage of cases where correct inference is made across all baskets with those showing the highest family‐wise error rate also presenting lower percentages of correct inference.

The BHM and BMA approach give the highest FWER and lowest percentage of correct inference in all baskets across Scenarios 2‐16. Under Scenarios 2 and 3 the ${\text{mEXNEX}}_{1/13}$ model has a FWER 5% smaller than the EXNEX model, producing similar values to the independent approach but with an improvement in power. Scenario 6 shows that models which typically inflate the error rate give the best proportions of correct inference across all baskets. The ${\text{mEXNEX}}_{1/13}$ model provides an increase of over 6% in comparison to an independent model. The percentage of all correct inference is smaller across scenarios where there are a few marginally effective baskets, that is, Scenarios 8‐10 and this lines up with larger inflation in error rates.

Similarly to the planned sample size case, these results confirm that the choice of c value makes a big impact in the performance of the ${\text{mEXNEX}}_{c}$ model. The c values were selected based on the planned sample size of 13 per basket and thus increments corresponding to 1 response were considered (ie, 0, 1/13, 2/13,…), however, when sample sizes are unequal it would be beneficial to look at other potential values such as 0, 0.05, 0.1 and so forth. A cut‐off of 0.05 can be shown to perform well in this unequal sample size scenario, whereas the choice of c=0 is far too conservative. In practice, when this occurs analysis can be conducted as specified in the trial protocol with the use of c based on planned sample sizes. Alternatively, one can re‐calibrate based on the realized sample sizes and compare to original analysis to determine if there are any significant differences. It would be recommended to include instructions within the trial protocol on how to adjust the cut‐off value for the ${\text{mEXNEX}}_{c}$ model once the realized sample sizes are known.

If the calibration of $\Delta_{\alpha }$ accounted for unequal sample sizes, similar patterns in performance of each method is observed but with the impact of small sample sizes particularly evident. Results from a further simulation under the realized sample size with re‐calibrated $\Delta_{\alpha }$ based on the unequal nature are provided in Section 2 of the supplementary materials.

## 4 ANALYSIS OF VE‐BASKET RESULTS USING INFORMATION BORROWING MODELS

This section revisits the analysis of the VE‐BASKET results using the described and proposed information borrowing methods. The data observed in the trial, and the posterior means for the response rate in each basket (and standard deviations) obtained by each method is given in Table 4. Also provided are the posterior probabilities of the response rate being greater than the null for each basket under each method, that is, the decision making probability used at the conclusion of the trial. For this analysis, prior and parameter choices are provided in Table 2. Within the modified EXNEX procedure, cut‐off values, c, are chosen from c=0,0.05,0.1,0.15,… Through a simulation akin to that in Section 3, cut‐off values of c=0.05 and c=0.1 were chosen, denoted ${\text{mEXNEX}}_{0.05}$ and ${\text{mEXNEX}}_{0.1}$ respectively.

**TABLE 4:** Data summary of the VE‐basket trial with posterior means of the response rates obtained using the various information borrowing models alongside their standard deviations in brackets, as well as the posterior probability that the response rate is greater than the null.

Trial data		NSCLC	Colorectal cancer	Cholangiocarcinoma	ECD/LCH	Thyroid cancer
Sample size		20	10	8	18	7
ORR		0.40	0.00	0.13	0.33	0.29
Basket		1	2	3	4	5
Independent	${\hat{p}}_{k}$	0.399 (0.11)	0.009 (0.03)	0.126 (0.11)	0.333 (0.11)	0.285 (0.16)
	$\mathbb{P}(p_{k}>0.15|D)$	0.996	0.008	0.325	0.968	0.777
BHM	${\hat{p}}_{k}$	0.362 (0.10)	0.097 (0.09)	0.170 (0.11)	0.309 (0.10)	0.267 (0.13)
	$\mathbb{P}(p_{k}>0.15|D)$	0.994	0.259	0.518	0.966	0.809
CBHM	${\hat{p}}_{k}$	0.398 (0.11)	0.012 (0.03)	0.125 (0.11)	0.331 (0.11)	0.281 (0.16)
	$\mathbb{P}(p_{k}>0.15|D)$	0.996	0.012	0.320	0.970	0.770
BMA	${\hat{p}}_{k}$	0.368 (0.09)	0.058 (0.08)	0.213 (0.09)	0.331 (0.09)	0.309 (0.12)
	$\mathbb{P}(p_{k}>0.15|D)$	0.997	0.120	0.648	0.981	0.899
EXNEX	${\hat{p}}_{k}$	0.384 (0.10)	0.059 (0.07)	0.171 (0.12)	0.326 (0.10)	0.288 (0.14)
	$\mathbb{P}(p_{k}>0.15|D)$	0.996	0.113	0.501	0.971	0.825
${\text{mEXNEX}}_{0.1}$	${\hat{p}}_{k}$	0.384 (0.10)	0.061 (0.06)	0.162 (0.11)	0.338 (0.10)	0.318 (0.13)
	$\mathbb{P}(p_{k}>0.15|D)$	0.997	0.089	0.454	0.983	0.904
${\text{mEXNEX}}_{0.05}$	${\hat{p}}_{k}$	0.398 (0.10)	0.061 (0.06)	0.162 (0.11)	0.328 (0.10)	0.301 (0.14)
	$\mathbb{P}(p_{k}>0.15|D)$	0.996	0.088	0.455	0.973	0.857

For the EXNEX and ${\text{mEXNEX}}_{c}$ models, specification of a prior probability vector, π, for assignment to the EX component is required. For each model, both the prior probability used and the posterior probabilities produced after model fit are listed below:

$$\begin{array}{llllll} & \text{Prior probability vectors:}\; & & \text{Posterior probability vectors:}\; & & \;\\ \text{EXNEX:}\;\; & \pi =(0.50,0.50,0.50,0.50,0.50),\; & & \pi =(0.36,0.50,0.42,0.39,0.41).\; & & \;\\ {\text{mEXNEX}}_{0.1}:\;\; & \pi =(0.74,0.00,0.00,0.79,0.74),\; & & \pi =(0.81,0.00,0.00,0.85,0.80).\; & & \;\\ {\text{mEXNEX}}_{0.05}:\;\; & \pi =(0.00,0.00,0.00,0.79,0.79),\; & & \pi =(0.00,0.00,0.00,0.74,0.75).\; & & \; \end{array}$$

The posterior probabilities for the EXNEX model decrease for all baskets compared to the prior values despite baskets 4 and 5 having homogeneous responses. In contrast, the ${\text{mEXNEX}}_{0.1}$ model increases between the prior and posterior probabilities which reflects the homogeneity of the response data. When c=0.05, we observe a decrease in posterior probabilities from the prior values, however, they are still greater than in the EXNEX model, which suggests greater sensitivity to the presence of both homogeneous and heterogeneous baskets.

The ${\text{mEXNEX}}_{0.05}$ model, only allows borrowing between baskets 4 and 5 with probability 0.79. This results in standard deviations lower in these baskets compared to the independent model. When c=0.1, the NSCLC basket is now included in the borrowing component with probability 0.74. This results in the estimated response rate in the first basket being pulled down as information is borrowed from baskets 4 and 5. The estimates and standard deviations for baskets 2 and 3 are identical for both c values as they are assigned to the NEX component. The ${\text{mEXNEX}}_{0.1}$ model has marginally smaller standard deviations compared to the EXNEX model with similar point estimates.

The results in Table 4 also demonstrate that using the independent model on baskets with small sample sizes leads to estimates with less precision due to the lack of borrowing. The CBHM results match that of the independent model due to the “strong” definition of heterogeneity in it is calibration procedure. There is clear heterogeneity between basket's 1 and 2 in which the ORR is 0.4 and 0 respectively and thus the CBHM treats all baskets as being independent with $\sigma^{2}\approx 383$.

The estimates using the BHM are pulled towards the common mean so the values are different to the ORR values, this is most evident in the second basket where the BHM estimates ${\hat{p}}_{2}=0.1$ while the ORR is 0. This is a direct result of the pull towards the common mean. A similar pattern is observed under the BMA method as the averaging procedure puts some weight on models that borrow between all baskets despite heterogeneity.

Focusing on the posterior probabilities of exceeding the null response rates, all methods give similar values for basket 1 which has a larger sample size and ORR value. This will likely lead to the treatment being deemed effective in basket 1 regardless of the method. However, the same cannot be said for basket 2 in which these probabilities vary across all methods, giving a value of approximately 0.01 under a stratified analysis, compared to 0.25 under the BHM. This could lead to potentially differing conclusions regarding the efficacy of a treatment based on the method used to analyse the results. Methods that borrow information between all baskets tend to have higher posterior probabilities when basket sample size is small compared to an independent analysis and methods such as the CBHM and ${\text{mEXNEX}}_{c}$ which borrow information to a lesser extent.

These results highlight that, as expected, the choice of borrowing method can impact inference made at the conclusion of a trial, especially in the case of heterogeneity across baskets. Heterogeneity causes a pull towards the common mean under most borrowing methods resulting in estimates different to the ORR values whilst having an even bigger impact on the decision probabilities used at the conclusion of the trail. However, the results also demonstrate benefits of borrowing in terms of increase in precision of point estimates, particularly when the sample size is small such as in the thyroid cancer basket which has just seven patients. From these differences in results, we would promote careful planning and pre‐trial evaluations to ensure that the borrowing method used is appropriate for the study.

## 5 DISCUSSION

Presented here were several Bayesian information borrowing techniques within a basket trial setting, alongside a proposed modification to the EXNEX model. Through simulation, the BHM, EXNEX model and a BMA approach were shown to have inflated error rates in the presence of baskets with heterogeneous response rates, while the CBHM lacks power in such a scenario.

Exploration of the methods applied to unequal sample sizes across baskets highlighted the inadequacy of the current calibration procedure in the CBHM which only previously considered equal sample sizes across baskets. A generalization of this calibration is made to handle the presence of unequal sample sizes, a situation that commonly arises in the clinical setting.

The proposed method has been shown to improve error control while increasing power over an independent analysis. This proposed method is robust to the presence of a heterogeneous basket as it is able to identify its difference in response and thus does not borrow information from it, while still retaining borrowing between homogeneous baskets with a probability determined by similarity in response through Hellinger distances.

The use of Hellinger distances has already been proposed for use in information borrowing in the basket trial setting by Zheng and Wason.^19^ However, they utilize the metric on data with continuous endpoints and a control arm, to stipulate a commensurate prior based on pairwise Hellinger distances. The ${\text{mEXNEX}}_{c}$ model uses averaged Hellinger distances to compute the prior probability of borrowing within the EXNEX model. Alternative distance metrics were considered but were shown to have less error control to that proposed in this article and are hence omitted.

The ${\text{mEXNEX}}_{c}$ model has been specified as a two‐step procedure, within which we first remove heterogeneous baskets to treat as independent and then utilize these Hellinger distances to specify the prior borrowing probabilities between the remaining baskets. In Section 5 of the supplementary material, explanation is provided as to why both of these steps are utilized in place of making just one of these modifications. Justifications are provided based on several thorough simulation studies, the first of which explored the performance of the one 1‐step vs 2‐step methods under the simulation setting outlined in Section 3 which highlighted the need for the first step—that is, removal of heterogeneous baskets—in order to control the type I error rate. We then continued exploration of the differences in approaches through a further simulation study that varied one design parameter at a time, that is, changed the number of baskets (of which further simulation studies under K=3 and K=10 baskets are presented in Section 6 of the supplementary material), changed the sample size or changed the target response rate. From this we concluded that the 2‐step ${\text{mEXNEX}}_{c}$ model as proposed in this article performs more favorably over a 1‐step modified EXNEX model when the sample size is very small or large, when we have a smaller number of baskets and when the target response rate is closer to the null response rate. This is a more realistic trial setting and hence why the 2‐step ${\text{mEXNEX}}_{c}$ model has been proposed, although an argument could be made in some cases to use just a 1‐step procedure in which heterogeneous baskets are removed and the remaining borrowing probabilities are fixed at 0.5.

The performance of the modified EXNEX model is reliant on the cut‐off specification for assigning a basket for independent analysis, which is selected to balance the trade‐off between power improvement and control of type I error rate. When chosen to favor power improvement, the proposed method reduces error rates in the presence of a single heterogeneous basket and improves power when all baskets are sensitive to treatment. However, when clusters of responses are observed, the proposed method increases the probability of borrowing between all baskets and hence error rates increase and the method performs similarly to the standard EXNEX model. Whereas, if the cut‐off is chosen to control error rates this inflation is not present across any of the simulation scenarios considered and power is improved in comparison to an independent analysis. As a result, implementing this newly proposed modified EXNEX model with a suitable cut‐off value, produces a model that either exceeds all other borrowing methods considered here in terms of performance of acts similarly to the standard EXNEX model.

A draw towards the standard EXNEX model is it is ability to borrow between multiple subsets of baskets by incorporating more than one exchangeability component in its mixture distribution in model (4). The ${\text{mEXNEX}}_{c}$ model could benefit from extension to allow for this feature. This would lead to better handling of borrowing within clusters of homogeneous responses.

Other alternative approaches for information borrowing in the basket trial setting are outlined in the literature, these include the MUCE design,^20^ Liu's two‐path approach^21^ and the RoBoT design^22^ to name a few. Comparisons between the proposed ${\text{mEXNEX}}_{c}$ model and the above methods have not yet been made.

Adaptive design features such as interim analyses with futility/efficacy stopping are desirable in most clinical trials and has been considered in the work by Jin et al,^3^ Berry et al,^7^ Chu and Yuan,^8^ and Psioda et al.^10^ However, no such adaptive design features were considered in this article which could be considered a limitation. The methodology described here could be extended to incorporate such features and future work into this aspect is being conducted.

## Supporting information


**Supplementary Material S1**: The following supporting information is available as part of the online article: full model specifications and simulation results for three studies: one for planned sample sizes, one for realized sample sizes without re‐calibration of the decision cut‐off, one *with* re‐calibration, as well as, results for family‐wise error rate and percentage of correct inference for all three cases. A further simulation study in which the true response rate is randomly generated is presented. Also provided in the online material is a discussion on the need for two steps in the proposed modified EXNEX model as opposed to just making a single alteration to the existing model, as well as, further simulation studies under a setting where the trial consists of either 3 or 10 baskets.

## Data Availability

All simulations were conducted through the computing software JAGS in R through the “rjags” package.^17^ No new data have been used in this publication. Simulations can be reproduced using the open accessible code available at https://github.com/LibbyDaniells/mEXNEX.

## APPENDIX A SIMULATION PRIOR AND PARAMETER SPECIFICATION

### A.1

For the simulation study in Section 3, priors are chosen to match those suggested in the models literature. The following priors are used for the simulation study:

- Independent model:
  $$\begin{array}{ll} & Y_{k}\;∼\;\text{Binomial}(n_{k},p_{k}),k=1,\ldots ,K\;\\ & \theta_{k}=\log(\frac{p_{k}}{1-p_{k}}),\;\\ & \theta_{k}\;∼\;N(\text{logit}(0.15),10^{2}),\; \end{array}$$
- Bayesian hierarchical model:
  $$\begin{array}{ll} & Y_{k}\;∼\;\text{Binomial}(n_{k},p_{k}),k=1,\ldots ,K\;\\ & \theta_{k}=\log(\frac{p_{k}}{1-p_{k}})\;∼\;N(\mu ,\sigma^{2}),\;\\ & \mu \;∼\;N(\text{logit}(0.15),10^{2}),\;\\ & \sigma \;∼\;\text{Half‐Cauchy}(0,25).\; \end{array}$$
- Calibrated Bayesian hierarchical model:
  $$\begin{array}{ll} & Y_{k}\;∼\;\text{Binomial}(n_{k},p_{k}),k=1,\ldots ,K\;\\ & \theta_{k}=\log(\frac{p_{k}}{1-p_{k}})\;∼\;N(\mu ,\sigma^{2}),\;\\ & \mu \;∼\;N(\text{logit}(0.15),10^{2}),\;\\ & \sigma^{2}=\exp\{a+b\log(T)\},\; \end{array}$$
  where, through tuning, a=−7.25 and b=5.86 based on a sample size of 13 per basket. The chi‐squared test statistic is used to compute T as in Equation (3).
- BMA: A weakly informative Beta prior is placed on the response rates so $p_{S_{j}}|{ℳ}_{j}\;∼\;\text{Beta}(a_{0},b_{0})$ where $a_{0}=q_{1}=0.45$ and $b_{0}=1-q_{1}=0.55$. The prior $f({ℳ}_{j})\;∼\;P_{j}^{2}$ is placed on the models, where $P_{j}$ is the number of distinct response rates in model j.
- EXNEX: For the standard EXNEX model equal prior mixture weights for the EX/NEX components are used and thus $\pi_{k}=0.5$ for all k baskets. A plausible guess of the true response rate is chosen to be $\rho_{k}=0.35$ (a value that is considered low but still indicative of a response) for all k baskets:
  $$\begin{array}{ll} Y_{k}∼\text{Binomial}(n_{k},p_{k}),\; & M_{1k}∼N(\mu ,\sigma^{2}),\text{(EX)}\;\\ \theta_{k}=\log(\frac{p_{k}}{1-p_{k}}),\; & \mu ∼N(\text{logit}(0.15),10^{2}),\;\\ \theta_{k}=\delta_{k}M_{1k}+(1-\delta_{k})M_{2k},\; & \sigma ∼\text{Half‐Normal}(0,1),\;\\ \delta_{k}∼\text{Bernoulli}(0.5),\; & M_{2k}∼N(-0.62,4.4^{2}),\text{(NEX)}\; \end{array}$$
  with the parameters of the NEX component computed through the following:^9^
  (A1)
  $$m_{k}=\log(\frac{\rho_{k}}{1-\rho_{k}}),\;\;\;\nu_{k}=\frac{1}{\rho_{k}}+\frac{1}{1-\rho_{k}}.$$
- Modified EXNEX: The same structure and prior choices as the standard EXNEX model with the exception of the prior on σ. Rather than applying the prior σ∼Half‐Normal(0,1) the prior is placed on $\sigma^{2}$, that is, $\sigma^{2}∼\text{Half‐Normal}(0,1)$. The mixture weights have a Bernoulli prior with prior parameter of success, $\pi_{k}$, which are calculated via the Hellinger distance with cut‐off c chosen to be 0 and 1/13.

## APPENDIX B SIMULATION RESULTS

### B.1

**TABLE B1:** Operating characteristics for a simulation based on the planned sample size of 13 per basket for Scenarios 1‐6.

	% Reject						
Sample size	13	13	13	13	13	% All correct	FWER
Scenario 1	0.15	0.15	0.15	0.15	0.15		
Independent	9.72	9.67	10.04	10.22	10.44	58.73	0.413
BHM	9.42	9.52	9.52	9.29	9.51	72.23	0.278
CBHM	9.77	9.93	9.60	9.65	9.90	76.63	0.234
BMA	10.07	10.04	9.78	10.21	9.73	68.05	0.320
EXNEX	10.35	9.95	10.17	9.97	10.35	62.69	0.373
${\text{mEXNEX}}_{1/13}$	9.78	10.05	10.40	10.00	10.09	61.18	0.388
${\text{mEXNEX}}_{0}$	9.57	10.06	9.18	9.84	9.61	60.04	0.400
Scenario 2	0.45	0.15	0.15	0.15	0.15		
Independent	87.34	10.41	10.61	10.08	10.08	56.79	0.351
BHM	85.51	16.53	16.82	17.16	17.12	45.72	0.419
CBHM	81.13	9.68	9.86	9.82	9.57	56.15	0.275
BMA	86.40	13.16	12.98	12.92	13.59	53.25	0.356
EXNEX	86.89	11.36	12.04	11.99	11.71	51.47	0.387
${\text{mEXNEX}}_{1/13}$	87.81	11.37	11.83	11.27	11.67	54.37	0.369
${\text{mEXNEX}}_{0}$	87.97	10.35	10.39	10.17	10.63	56.29	0.352
Scenario 3	0.45	0.45	0.15	0.15	0.15		
Independent	88.36	88.67	10.24	9.80	9.97	57.35	0.271
BHM	91.62	91.56	21.70	21.59	22.32	45.96	0.428
CBHM	84.63	84.38	10.44	10.79	10.66	52.99	0.246
BMA	89.93	89.67	17.96	18.33	18.66	49.36	0.358
EXNEX	89.92	90.00	12.55	12.97	12.79	55.04	0.321
${\text{mEXNEX}}_{1/13}$	89.53	89.20	12.56	12.41	12.59	54.24	0.316
${\text{mEXNEX}}_{0}$	88.39	88.69	10.44	10.59	10.92	55.60	0.282
Scenario 4	0.45	0.45	0.45	0.15	0.15		
Independent	88.37	87.95	88.29	10.37	9.62	56.03	0.189
BHM	94.19	94.03	93.90	29.67	30.44	41.43	0.458
CBHM	85.94	86.48	86.22	12.29	12.06	49.82	0.200
BMA	92.38	92.72	93.09	23.80	23.24	44.18	0.390
EXNEX	91.13	91.08	90.96	13.12	13.13	57.75	0.230
${\text{mEXNEX}}_{1/13}$	90.98	90.78	90.68	13.84	14.05	56.37	0.243
${\text{mEXNEX}}_{0}$	89.48	89.20	88.89	10.61	10.86	56.53	0.204
Scenario 5	0.45	0.45	0.45	0.45	0.15		
Independent	88.30	87.81	87.46	88.51	10.10	54.14	0.101
BHM	96.55	96.13	96.44	96.04	42.11	47.17	0.421
CBHM	87.99	87.94	87.89	88.19	16.65	47.18	0.167
BMA	94.69	94.44	94.92	94.62	24.01	60.37	0.240
EXNEX	91.28	91.14	91.70	90.91	16.12	56.87	0.161
${\text{mEXNEX}}_{1/13}$	91.43	91.72	91.63	91.52	14.86	58.60	0.149
${\text{mEXNEX}}_{0}$	89.54	89.44	89.40	89.19	11.20	56.54	0.112
Scenario 6	0.45	0.45	0.45	0.45	0.45		
Independent	88.28	87.66	88.16	88.09	87.95	52.77	
BHM	97.94	98.28	98.13	98.23	97.87	91.53	
CBHM	91.28	91.48	91.06	91.16	91.48	66.72	
BMA	95.24	95.49	95.62	95.30	95.84	79.88	
EXNEX	92.42	92.61	91.98	91.96	91.98	68.39
${\text{mEXNEX}}_{1/13}$	91.64	92.13	92.06	91.60	91.94	66.38	
${\text{mEXNEX}}_{0}$	89.78	89.57	89.65	89.92	89.92	58.38	

**TABLE B2:** Operating characteristics for a simulation based on the planned sample size of 13 per basket for Scenarios 7‐10.

	% Reject						
Sample size	13	13	13	13	13	% All correct	FWER
Scenario 7	0.35	0.15	0.15	0.15	0.15		
Independent	66.04	9.11	9.06	9.03	9.21	45.47	0.317
BHM	63.60	15.13	15.31	15.17	15.16	32.43	0.375
CBHM	55.78	9.13	9.22	8.96	9.26	38.29	0.234
BMA	65.20	13.14	13.37	12.89	13.26	37.72	0.353
EXNEX	67.77	11.27	11.46	11.25	11.31	40.06	0.371
${\text{mEXNEX}}_{1/13}$	68.34	11.23	11.02	10.90	11.17	42.22	0.353
${\text{mEXNEX}}_{0}$	68.34	10.12	9.84	9.85	10.16	44.90	0.353
Scenario 8	0.35	0.35	0.35	0.15	0.15		
Independent	66.80	64.94	65.95	9.15	9.32	23.70	0.177
BHM	80.04	79.02	80.02	28.93	28.91	24.22	0.426
CBHM	66.33	65.28	65.86	16.20	16.22	20.62	0.225
BMA	77.39	76.45	77.23	23.73	23.78	19.52	0.391
EXNEX	73.23	71.79	73.12	13.39	13.71	28.66	0.231
${\text{mEXNEX}}_{1/13}$	72.98	71.60	72.82	14.40	14.43	27.53	0.245
${\text{mEXNEX}}_{0}$	69.35	67.97	69.41	10.73	10.84	26.76	0.203
Scenario 9	0.45	0.35	0.35	0.15	0.15		
Independent	86.79	65.64	66.13	9.18	8.98	31.18	0.173
BHM	93.78	79.49	80.73	29.23	29.07	28.67	0.435
CBHM	86.56	65.29	66.40	14.29	14.42	27.79	0.213
BMA	92.90	76.68	77.27	24.01	24.15	25.25	0.395
EXNEX	90.94	71.88	73.18	13.35	13.39	36.03	0.230
${\text{mEXNEX}}_{1/13}$	90.71	71.74	73.02	13.83	13.85	35.40	0.238
${\text{mEXNEX}}_{0}$	88.57	68.31	70.05	10.96	10.86	34.07	0.205
Scenario 10	0.45	0.45	0.35	0.35	0.15		
Independent	87.23	86.71	66.48	66.23	9.11	30.24	0.091
BHM	95.87	95.57	86.07	86.09	40.76	35.86	0.408
CBHM	88.69	87.89	69.31	69.35	18.48	26.39	0.185
BMA	94.97	94.81	83.30	83.24	27.25	46.40	0.273
EXNEX	91.56	90.86	74.23	74.02	17.25	34.31	0.173
${\text{mEXNEX}}_{1/13}$	91.59	91.10	74.52	74.30	16.00	36.47	0.160
${\text{mEXNEX}}_{0}$	89.72	89.26	70.92	70.69	11.19	35.71	0.112

**TABLE B3:** Operating characteristics for a simulation based on the realized sample size of 20, 10, 8, 18, and 7 across the five baskets for Scenarios 1‐6.

	% Reject						
Sample size	20	10	8	18	7	% All correct	FWER
Scenario 1	0.15	0.15	0.15	0.15	0.15		
Independent	6.36	4.83	10.47	6.14	7.39	69.49	0.305
BHM	11.15	9.11	8.46	11.07	8.59	70.66	0.293
CBHM	8.49	7.28	9.9	6.86	9.22	72.28	0.277
BMA	10.41	9.21	9.37	10.34	9.75	65.99	0.340
EXNEX	9.22	7.42	10.33	10.56	7.19	65.56	0.344
${\text{mEXNEX}}_{1/13}$	9.48	12.30	10.31	11.70	8.04	58.98	0.410
${\text{mEXNEX}}_{0}$	6.85	4.79	10.83	6.04	7.42	68.61	0.314
Scenario 2	0.45	0.15	0.15	0.15	0.15		
Independent	94.61	4.83	10.40	5.91	7.67	70.02	0.261
BHM	95.61	18.43	16.32	17.29	15.18	53.00	0.434
CBHM	94.60	7.75	12.19	7.72	10.26	68.58	0.272
BMA	96.05	13.74	13.66	14.82	13.16	59.76	0.373
EXNEX	95.28	12.00	10.77	11.68	8.80	61.92	0.346
${\text{mEXNEX}}_{1/13}$	95.40	14.13	10.76	11.75	8.51	60.60	0.361
${\text{mEXNEX}}_{0}$	94.27	4.93	10.45	5.85	7.47	69.97	0.258
Scenario 3	0.45	0.45	0.15	0.15	0.15		
Independent	94.14	73.33	10.92	5.57	7.83	54.14	0.222
BHM	97.62	87.94	21.31	21.56	22.44	47.25	0.431
CBHM	94.83	75.62	12.32	7.75	10.15	53.96	0.232
BMA	96.46	84.12	17.93	17.97	19.00	47.87	0.372
EXNEX	96.27	82.59	11.05	12.31	11.54	54.81	0.287
${\text{mEXNEX}}_{1/13}$	95.90	84.97	11.49	12.36	11.09	57.33	0.290
${\text{mEXNEX}}_{0}$	94.70	73.27	10.49	5.86	7.45	54.22	0.219
Scenario 4	0.45	0.45	0.45	0.15	0.15		
Independent	94.12	73.39	78.11	5.53	7.61	47.24	0.127
BHM	98.10	91.41	86.00	28.53	30.15	39.06	0.447
CBHM	95.20	75.65	79.04	9.27	10.93	45.85	0.157
BMA	97.09	88.34	82.69	21.92	25.58	37.73	0.410
EXNEX	96.70	87.15	78.40	13.43	17.31	47.47	0.267
${\text{mEXNEX}}_{1/13}$	96.25	87.37	78.90	12.15	14.16	50.08	0.234
${\text{mEXNEX}}_{0}$	94.70	73.44	77.75	5.89	7.30	47.11	0.128
Scenario 5	0.45	0.45	0.45	0.45	0.15		
Independent	94.92	73.29	78.10	90.83	7.96	45.46	0.080
BHM	98.72	94.08	90.92	98.39	51.36	35.06	0.514
CBHM	95.16	79.55	81.27	92.30	24.01	36.52	0.240
BMA	98.06	89.68	90.46	97.36	28.21	56.25	0.282
EXNEX	98.03	89.88	79.65	95.84	27.58	46.83	0.276
${\text{mEXNEX}}_{1/13}$	96.27	88.98	80.34	95.95	17.93	53.30	0.179
${\text{mEXNEX}}_{0}$	94.57	73.22	78.47	90.93	7.23	45.79	0.723
Scenario 6	0.45	0.45	0.45	0.45	0.45		
Independent	94.37	73.47	77.77	90.76	68.12	33.05	
BHM	99.39	96.97	94.72	99.01	94.80	87.29	
CBHM	95.70	84.24	84.82	94.06	80.99	59.07	
BMA	98.16	90.15	90.92	97.59	89.61	70.66	
EXNEX	98.29	89.75	83.66	96.37	88.21	64.59	
${\text{mEXNEX}}_{1/13}$	96.77	89.98	79.90	95.60	78.18	52.27	
${\text{mEXNEX}}_{0}$	94.57	73.11	77.92	90.90	69.20	33.54	

**TABLE B4:** Operating characteristics for a simulation based on the realized sample size of 20, 10, 8, 18, and 7 across the five baskets for Scenarios 7‐12.

	% Reject						
Sample size	20	10	8	18	7	% All correct	FWER
Scenario 7	0.35	0.15	0.15	0.15	0.15		
Independent	76.02	4.94	10.40	4.99	7.20	57.36	0.249
BHM	80.18	17.27	15.52	17.46	14.69	43.94	0.399
CBHM	75.84	9.30	13.08	8.98	11.39	53.72	0.267
BMA	80.10	13.81	13.52	15.06	13.48	48.14	0.371
EXNEX	78.75	12.00	10.56	11.91	8.77	50.38	0.341
${\text{mEXNEX}}_{1/13}$	79.25	13.25	11.01	11.77	9.68	50.38	0.357
${\text{mEXNEX}}_{0}$	76.02	4.94	10.40	5.90	7.20	56.84	0.257
Scenario 8	0.35	0.35	0.35	0.15	0.15		
Independent	76.02	47.90	57.64	4.86	7.20	19.18	0.118
BHM	88.00	74.06	68.46	27.85	27.75	22.18	0.414
CBHM	78.39	54.96	60.62	14.03	15.81	18.10	0.202
BMA	85.28	69.90	65.30	22.84	23.12	19.26	0.389
EXNEX	83.73	67.45	58.24	13.66	15.33	24.78	0.246
${\text{mEXNEX}}_{1/13}$	81.89	66.64	59.23	12.33	14.58	23.85	0.238
${\text{mEXNEX}}_{0}$	76.02	47.93	57.64	5.76	7.20	18.99	0.126
Scenario 9	0.45	0.35	0.35	0.15	0.15		
Independent	94.36	47.90	57.64	5.22	7.20	23.37	0.121
BHM	97.99	75.36	68.87	27.28	27.86	24.42	0.420
CBHM	95.02	52.26	60.02	10.73	12.31	22.56	0.171
BMA	97.25	69.49	64.70	21.19	23.47	22.24	0.379
EXNEX	96.76	68.53	58.20	13.04	15.93	28.11	0.249
${\text{mEXNEX}}_{1/13}$	96.12	67.52	59.08	12.31	13.79	28.57	0.230
${\text{mEXNEX}}_{0}$	94.36	47.90	57.64	5.86	7.20	23.21	0.127
Scenario 10	0.45	0.45	0.35	0.35	0.15		
Independent	94.36	72.77	57.64	67.87	7.20	24.85	0.072
BHM	98.87	93.39	79.56	90.52	46.96	28.35	0.496
CBHM	95.57	79.91	64.17	74.09	24.04	20.08	0.240
BMA	98.03	89.48	78.91	87.79	27.76	45.58	0.278
EXNEX	97.94	89.39	61.24	82.50	25.36	30.02	0.254
${\text{mEXNEX}}_{1/13}$	96.66	88.21	61.64	82.01	18.18	33.81	0.182
${\text{mEXNEX}}_{0}$	94.36	72.83	57.64	69.63	7.20	25.39	0.072
Scenario 11	0.15	0.15	0.15	0.15	0.45		
Independent	6.75	4.94	10.40	4.91	68.65	51.58	0.244
BHM	15.60	14.74	13.30	15.13	63.74	31.89	0.384
CBHM	8.47	7.22	11.26	6.76	68.78	49.76	0.248
BMA	12.07	10.87	10.99	12.22	70.21	45.60	0.328
EXNEX	10.71	9.97	10.36	11.60	68.89	44.60	0.335
${\text{mEXNEX}}_{1/13}$	10.22	12.99	10.52	11.36	69.15	41.90	0.328
${\text{mEXNEX}}_{0}$	6.75	4.94	10.40	5.90	68.65	51.06	0.253
Scenario 12	0.15	0.15	0.45	0.15	0.45		
Independent	6.75	4.94	78.46	5.19	68.65	45.88	0.160
BHM	19.81	19.69	77.71	19.10	72.94	30.79	0.416
CBHM	8.45	71.40	76.84	7.07	69.58	45.28	0.176
BMA	14.06	13.59	76.02	15.03	72.10	32.99	0.328
EXNEX	13.61	13.76	78.05	12.07	69.18	35.06	0.326
${\text{mEXNEX}}_{1/13}$	12.05	14.42	78.53	11.57	69.70	35.86	0.319
${\text{mEXNEX}}_{0}$	6.75	4.94	78.46	5.77	68.65	45.49	0.166

**TABLE B5:** Operating characteristics for a simulation based on the realized sample size of 20, 10, 8, 18, and 7 across the five baskets for Scenarios 13‐16.

	% Reject						
Sample size	20	10	8	18	7	% All correct	FWER
Scenario 13	0.15	0.45	0.45	0.15	0.45		
Independent	6.75	72.77	78.46	4.87	68.65	35.11	0.113
BHM	24.43	88.84	83.42	24.96	80.68	33.21	0.393
CBHM	8.80	74.51	78.85	7.73	69.96	34.72	0.138
BMA	16.49	84.14	80.71	18.01	76.41	32.74	0.301
EXNEX	16.08	84.34	78.56	12.59	70.79	34.35	0.262
${\text{mEXNEX}}_{1/13}$	12.96	85.24	78.71	11.93	70.96	36.75	0.230
${\text{mEXNEX}}_{0}$	6.75	72.77	78.46	5.93	68.65	34.77	0.123
Scenario 14	0.15	0.45	0.45	0.45	0.45		
Independent	6.75	72.77	78.46	90.51	68.65	33.62	0.068
BHM	33.15	92.31	89.48	98.17	88.46	45.93	0.332
CBHM	10.60	75.58	79.79	91.72	71.10	33.01	0.106
BMA	16.88	89.36	86.45	97.33	87.04	56.35	0.169
EXNEX	16.72	89.33	79.41	96.53	79.03	45.97	0.167
${\text{mEXNEX}}_{1/13}$	13.64	88.06	79.75	96.36	73.96	44.16	0.136
${\text{mEXNEX}}_{0}$	6.75	72.82	78.46	91.20	68.65	33.72	0.068
Scenario 15	0.45	0.15	0.15	0.15	0.45		
Independent	94.36	4.94	10.40	5.05	68.65	52.35	0.191
BHM	97.24	22.52	20.75	21.14	76.40	38.11	0.433
CBHM	94.60	7.75	12.61	7.88	70.05	51.10	0.213
BMA	96.31	16.60	16.98	17.12	74.34	41.21	0.364
EXNEX	95.90	16.22	10.77	12.33	70.05	42.54	0.336
${\text{mEXNEX}}_{1/13}$	95.77	15.27	11.14	11.85	70.29	43.45	0.325
${\text{mEXNEX}}_{0}$	94.36	4.95	10.40	5.83	68.65	51.79	0.198
Scenario 16	0.45	0.15	0.45	0.15	0.45		
Independent	94.36	4.94	78.46	4.91	68.65	46.49	0.097
BHM	98.25	27.38	84.74	25.99	82.68	37.57	0.402
CBHM	94.71	8.36	79.27	8.70	70.39	44.99	0.134
BMA	97.20	17.75	82.49	20.40	79.90	38.51	0.338
EXNEX	97.15	17.80	78.74	12.98	72.42	38.90	0.278
${\text{mEXNEX}}_{1/13}$	96.39	17.06	79.08	11.88	72.42	39.00	0.268
${\text{mEXNEX}}_{0}$	94.36	4.94	78.46	6.00	68.65	45.88	0.107
