# Supplementary material for: A comparison of Bayesian information borrowing methods in basket trials and a novel proposal of modified exchangeability‐nonexchangeability method
Source: Stat Med. 2023 Aug 23;42(24):4392–417. doi: 10.1002/sim.9867 (PMC10962580; doi:10.1002/sim.9867)
Supplement: Supplementary file 1 — Supplementary Material S1: The following supporting information is available as part of the online article: full model specifications and simulation results for three studies: one for planned sample sizes, one for realized sample sizes without re‐calibration of the decision cut‐off, one with re‐calibration, as well as, results for family‐wise error rate and percentage of correct inference for all three cases. A further simulation study in which the true response rate is randomly generated is presented. Also provided in the online material is a discussion on the need for two steps in the proposed modified EXNEX model as opposed to just making a single alteration to the existing model, as well as, further simulation studies under a setting where the trial consists of either 3 or 10 baskets. [file SIM-42-4392-s001.pdf]

Supplementary Material: A comparison of Bayesian information borrowing methods in basket trials and a novel proposal of modified exchangeability-nonexchangeability method

Libby Daniells, Pavel Mozgunov, Alun Bedding, Thomas Jaki

1 Simulation Results for Section 3 of the Main Text

Table S1: Calibrated Δ_α values for the simulation study based on a planned sample size of 13 per basket. These cut-offs are also applied to the realized sample size scenario without re-calibration.

	Δ_α
Independent	0.905
BHM	0.831
CBHM	0.907
BMA	0.871
EXNEX	0.868
mEXNEX$_{1/13}$	0.880
mEXNEX$_0$	0.920

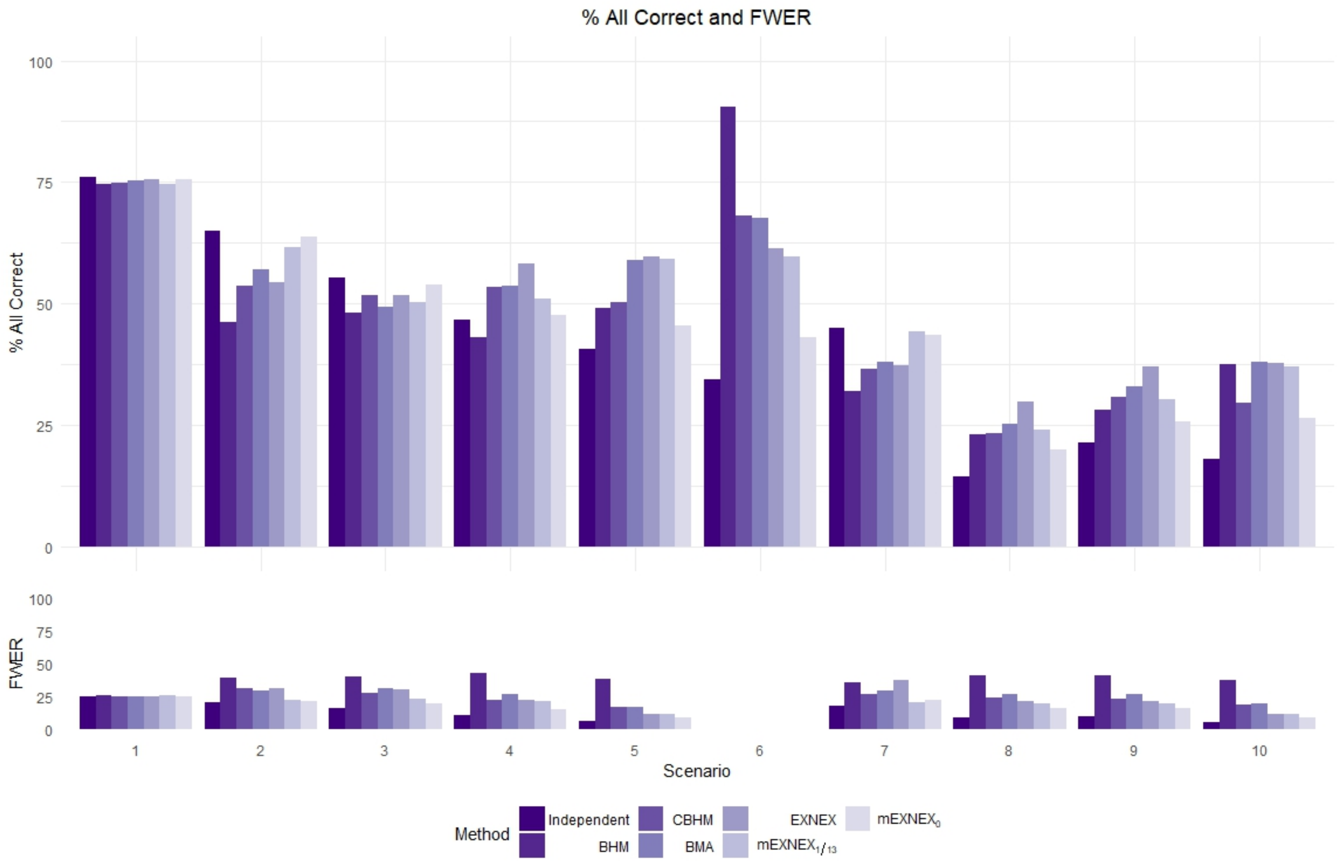

Figure S1: The family-wise error rate (FWER) and percentage of simulated data sets within which correct inference is made across all baskets (% All Correct) for each method under each data scenario based on a planned sample size of 13 patients per basket.

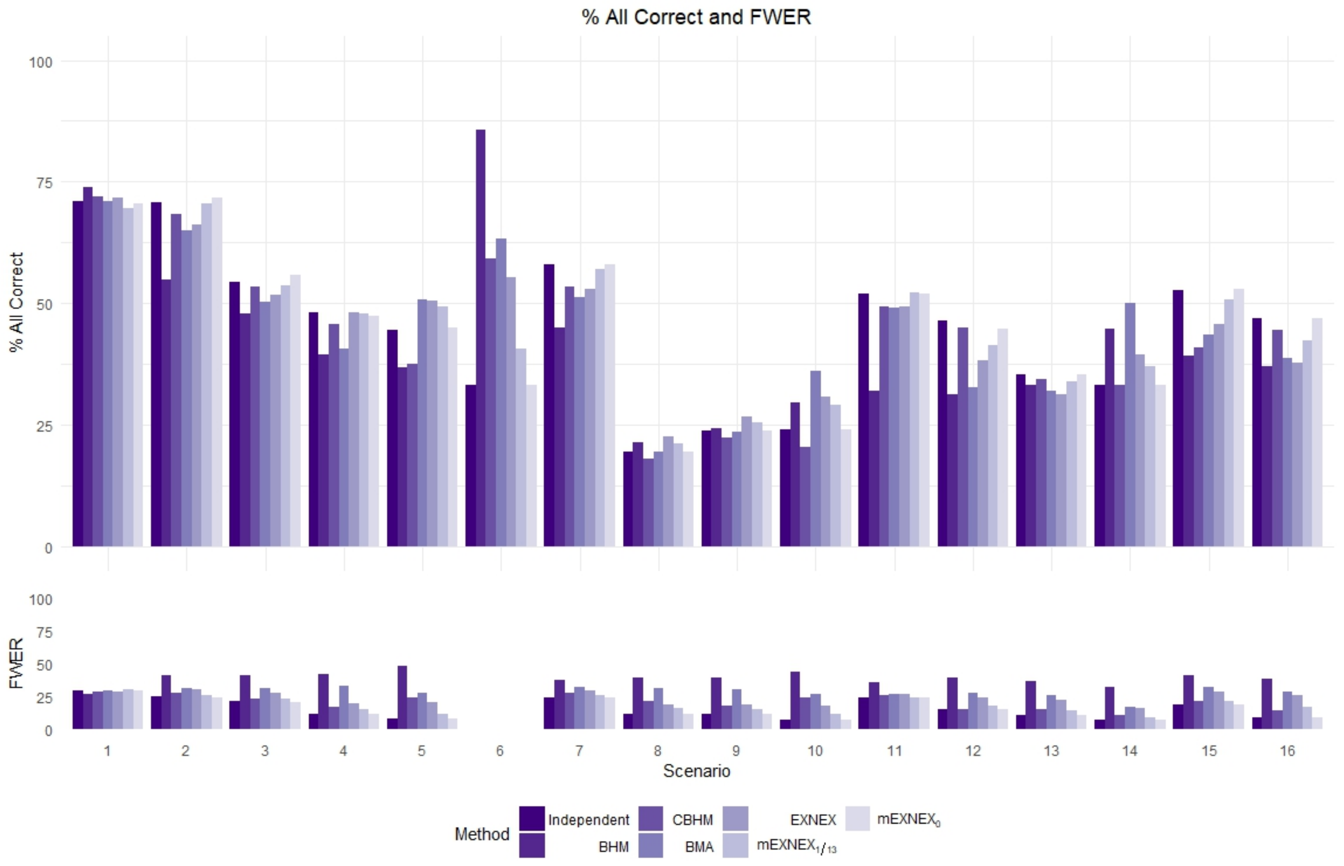

Figure S2: The family-wise error rate (FWER) and percentage of simulated datasets in which the correct inference is made across all baskets (% All Correct) for each method under each data scenario based on realized sample sizes of 20, 10, 8, 18 and 7 across the 5 baskets.

2 Simulation Study for Realized Sample Size With Re-Calibrated Δ_α Values

In addition to those in the main text, a further simulation study was conducted on the realized sample size case of 20, 10, 8, 18 and 7 patients across the five baskets. In the previous study, the decision cut-off, Δ_α, was calibrated under a null scenario based on $n_k = 13$ patients in each basket and applied to the realized sample sizes. In this simulation, the Δ_α values are re-calibrated based on the unequal sample sizes to again achieve a basket specific type I error rate of 10% under the null scenario.

Table S2: Re-calibrated Δ_α values for the realized sample sizes of 20, 10, 8, 18 and 7 across the five baskets.

	$\Delta_{\alpha 1}$	$\Delta_{\alpha 2}$	$\Delta_{\alpha 3}$	$\Delta_{\alpha 4}$	$\Delta_{\alpha 5}$
Independent	0.859	0.862	0.922	0.899	0.786
BHM	0.852	0.811	0.806	0.844	0.806
CBHM	0.962	0.912	0.855	0.964	0.820
BMA	0.876	0.856	0.865	0.876	0.863
EXNEX	0.861	0.849	0.868	0.875	0.822
$\text{mEXNEX}_{1/13}$	0.877	0.887	0.907	0.903	0.836
mEXNEX_0	0.880	0.888	0.937	0.916	0.831

Table S3: Operating characteristics for a simulation based on the realized sample size of 20, 10, 8, 18 and 7 across the baskets under data scenarios 1-6, with re-calibration of Δ_α to take into account the unequal sample sizes.

	% Reject					% All Correct	FWER
Sample Size	20	10	8	18	7		
**Scenario 1**	**0.15**	**0.15**	**0.15**	**0.15**	**0.15**		
Independent	10.45	10.75	9.81	9.38	9.39	59.19	0.408
BHM	9.34	10.17	10.18	9.62	10.48	69.84	0.316
CBHM	10.21	10.11	10.21	10.62	10.05	68.36	0.316
BMA	10.15	9.47	10.17	9.62	9.92	65.69	0.343
EXNEX	10.16	9.99	9.76	9.55	9.68	64.33	0.357
$\text{mEXNEX}_{1/13}$	10.14	9.12	9.98	9.52	9.54	62.45	0.376
mEXNEX_0	9.72	9.88	9.20	9.93	10.21	59.77	0.402
**Scenario 2**	**0.45**	**0.15**	**0.15**	**0.15**	**0.15**		
Independent	95.51	10.35	10.18	10.42	9.57	62.38	0.348
BHM	94.78	20.90	18.29	17.02	19.00	49.18	0.467
CBHM	94.92	13.37	12.04	13.12	9.97	60.71	35.23
BMA	95.62	13.87	13.23	13.57	13.62	60.06	0.366
EXNEX	95.52	15.93	10.54	11.64	14.32	57.48	0.392
$\text{mEXNEX}_{1/13}$	95.67	10.76	10.49	11.29	13.01	62.12	0.348
mEXNEX_0	95.22	10.53	8.94	9.51	9.86	63.11	0.398
**Scenario 3**	**0.45**	**0.45**	**0.15**	**0.15**	**0.15**		
Independent	95.75	80.2	10.07	9.91	9.35	56.38	0.266
BHM	96.58	89.87	24.72	19.78	25.81	44.34	0.468
CBHM	94.88	82.90	12.75	13.39	10.27	55.66	0.281
BMA	96.45	84.45	19.17	16.82	20.09	47.48	0.379
EXNEX	96.27	87.40	10.89	12.53	22.49	50.69	0.373
$\text{mEXNEX}_{1/13}$	96.36	81.36	10.74	11.47	15.13	51.41	0.315
mEXNEX_0	95.48	79.57	9.10	9.89	9.34	56.30	0.258
**Scenario 4**	**0.45**	**0.45**	**0.45**	**0.15**	**0.15**		
Independent	95.99	80.41	75.40	9.93	9.26	47.03	0.184
BHM	97.93	92.13	87.46	25.06	32.78	41.31	0.434
CBHM	95.66	84.73	78.77	14.29	11.27	49.50	0.205
BMA	97.23	89.25	82.89	19.95	25.72	39.84	0.395
EXNEX	97.41	89.41	78.13	12.79	26.96	43.29	0.356
$\text{mEXNEX}_{1/13}$	96.62	84.43	77.87	11.92	17.84	45.91	0.265
mEXNEX_0	95.36	79.89	74.86	9.58	10.11	46.68	0.189
**Scenario 5**	**0.45**	**0.45**	**0.45**	**0.45**	**0.15**		
Independent	95.65	79.53	75.31	94.53	9.14	48.92	0.091
BHM	98.77	94.74	93.09	98.14	56.04	32.36	0.560
CBHM	95.80	86.36	80.95	95.93	24.02	43.59	0.240
BMA	98.11	89.33	91.39	97.80	27.96	56.46	0.280
EXNEX	98.11	90.17	80.77	95.75	28.48	47.97	0.285
$\text{mEXNEX}_{1/13}$	97.47	86.96	77.82	96.32	19.48	51.70	0.195
mEXNEX_0	95.56	79.96	75.49	93.87	9.98	49.22	0.098
**Scenario 6**	**0.45**	**0.45**	**0.45**	**0.45**	**0.45**		
Independent	95.75	79.81	75.37	94.22	71.10	38.88	
BHM	99.11	97.17	96.10	98.86	95.87	89.11	
CBHM	96.18	91.23	85.48	96.36	82.04	64.41	
BMA	97.79	90.15	92.17	97.53	89.91	71.44	
EXNEX	98.10	90.30	83.96	96.19	89.55	65.24	
$\text{mEXNEX}_{1/13}$	97.50	87.58	78.17	95.73	80.25	50.10	
mEXNEX_0	95.82	79.31	74.10	93.79	70.47	37.04	

Table S4: Operating characteristics for a simulation based on the realized sample size of 20, 10, 8, 18 and 7 across the baskets under data scenarios 7-12, with re-calibration of Δ_α to take into account the unequal sample sizes.

	% Reject					% All Correct	FWER
Sample Size	20	10	8	18	7		
**Scenario 7**	**0.35**	**0.15**	**0.15**	**0.15**	**0.15**		
Independent	78.83	8.21	9.13	8.93	7.84	55.53	0.301
BHM	78.00	19.50	17.96	16.31	17.63	40.12	0.428
CBHM	60.63	10.06	16.72	6.66	15.74	42.67	0.297
BMA	79.81	14.83	14.57	14.46	14.35	47.13	0.382
EXNEX	79.02	13.78	8.98	10.96	7.50	49.89	0.337
$\text{mEXNEX}_{1/13}$	80.24	11.12	10.11	10.42	13.19	51.91	0.342
mEXNEX_0	79.68	10.70	9.35	9.65	9.67	52.75	0.340
**Scenario 8**	**0.35**	**0.35**	**0.35**	**0.15**	**0.15**		
Independent	78.90	54.35	53.30	9.02	8.06	19.57	0.165
BHM	86.15	76.36	71.75	25.51	31.72	23.43	0.421
CBHM	62.79	55.41	62.90	10.38	20.11	14.05	0.222
BMA	84.68	71.07	66.84	21.77	24.01	20.14	0.389
EXNEX	84.09	69.48	55.88	11.64	10.13	28.66	0.196
$\text{mEXNEX}_{1/13}$	82.72	63.68	57.25	11.49	19.66	21.76	0.280
mEXNEX_0	79.86	59.62	54.05	9.56	9.77	21.98	0.184
**Scenario 9**	**0.45**	**0.35**	**0.35**	**0.15**	**0.15**		
Independent	95.38	54.25	53.27	9.16	7.85	23.28	0.163
BHM	97.38	77.04	72.24	25.19	31.62	26.40	0.426
CBHM	88.33	52.07	61.30	8.06	15.46	20.90	0.179
BMA	97.04	70.87	66.15	20.11	24.20	23.53	0.378
EXNEX	96.96	70.47	56.85	11.73	10.24	32.29	0.197
$\text{mEXNEX}_{1/13}$	96.43	63.35	57.48	11.54	17.70	26.03	0.261
mEXNEX_0	95.28	57.62	54.19	9.42	9.70	24.48	0.182
**Scenario 10**	**0.45**	**0.45**	**0.35**	**0.35**	**0.15**		
Independent	95.24	77.09	53.15	76.13	7.90	27.07	0.079
BHM	98.66	93.97	82.81	89.51	51.73	25.62	0.517
CBHM	89.02	78.64	64.80	67.77	27.64	15.17	0.276
BMA	98.01	89.56	79.79	86.92	27.83	45.37	0.278
EXNEX	98.01	89.60	57.61	81.70	20.38	30.05	0.204
$\text{mEXNEX}_{1/13}$	97.07	86.30	58.17	81.43	21.49	31.68	0.215
mEXNEX_0	95.42	79.57	53.87	77.13	9.61	28.49	0.096
**Scenario 11**	**0.15**	**0.15**	**0.15**	**0.15**	**0.45**		
Independent	9.17	8.21	8.99	8.97	69.47	47.74	0.309
BHM	13.40	16.85	15.25	13.73	67.61	34.84	0.388
CBHM	4.43	8.21	14.34	5.60	72.15	53.70	0.232
BMA	11.72	12.01	11.74	11.83	70.62	44.75	0.336
EXNEX	10.96	11.43	7.49	9.76	68.84	46.29	0.301
$\text{mEXNEX}_{1/13}$	10.99	10.20	10.10	9.99	70.76	44.71	0.336
mEXNEX_0	9.74	9.60	9.23	9.40	71.35	47.65	0.326
**Scenario 12**	**0.15**	**0.15**	**0.45**	**0.15**	**0.45**		
Independent	9.18	8.21	74.48	9.15	69.43	39.71	0.243
BHM	17.76	21.71	79.62	17.42	76.08	33.29	0.404
CBHM	3.92	7.67	79.50	5.28	71.84	49.32	0.131
BMA	13.61	14.94	77.06	14.58	72.83	33.63	0.337
EXNEX	14.02	15.26	69.33	11.24	68.75	29.17	0.338
$\text{mEXNEX}_{1/13}$	12.97	12.04	77.20	10.84	72.08	35.89	0.299
mEXNEX_0	10.20	10.19	74.94	9.72	71.25	39.35	0.268

Table S5: Operating characteristics for a simulation based on the realized sample size of 20, 10, 8, 18 and 7 across the baskets under data scenarios 13-16, with re-calibration of Δ_α to take into account the unequal sample sizes.

	% Reject					% All Correct	FWER
Sample Size	20	10	8	18	7		
**Scenario 13**	**0.15**	**0.45**	**0.45**	**0.15**	**0.45**		
Independent	9.14	76.96	74.06	9.09	69.41	33.02	0.175
BHM	21.90	89.88	85.67	23.05	84.44	39.16	0.362
CBHM	4.29	74.62	79.71	5.77	71.35	37.84	0.080
BMA	16.20	85.69	81.50	16.87	77.37	35.37	0.289
EXNEX	16.27	86.11	76.40	11.70	69.08	35.04	0.261
$\text{mEXNEX}_{1/13}$	14.06	81.32	77.90	11.55	74.14	34.28	0.239
mEXNEX_0	10.31	79.30	75.32	9.46	71.20	34.64	0.189
**Scenario 14**	**0.15**	**0.45**	**0.45**	**0.45**	**0.45**		
Independent	9.26	77.06	74.50	93.93	69.29	34.41	0.093
BHM	31.41	93.18	91.31	97.98	89.91	48.62	0.314
CBHM	6.85	75.15	80.19	89.48	72.20	34.61	0.069
BMA	16.75	89.55	87.46	97.15	87.68	57.31	0.168
EXNEX	16.73	89.50	78.43	96.35	71.87	40.29	0.167
$\text{mEXNEX}_{1/13}$	14.73	85.30	78.49	96.17	77.37	42.06	0.147
mEXNEX_0	9.80	78.84	75.07	94.37	71.28	35.87	0.098
**Scenario 15**	**0.45**	**0.15**	**0.15**	**0.15**	**0.45**		
Independent	95.18	8.24	9.07	9.29	69.31	49.74	0.243
BHM	96.52	24.35	23.96	19.89	79.27	37.98	0.451
CBHM	87.52	7.87	13.46	5.75	71.08	48.10	0.256
BMA	96.23	17.26	17.89	16.21	75.09	41.05	0.371
EXNEX	96.06	17.08	10.26	11.73	69.01	42.27	0.342
$\text{mEXNEX}_{1/13}$	96.13	12.46	10.38	11.20	73.12	46.82	0.294
mEXNEX_0	95.48	9.86	9.38	9.68	71.24	49.98	0.263
**Scenario 16**	**0.45**	**0.15**	**0.45**	**0.15**	**0.45**		
Independent	95.31	8.03	74.65	8.94	69.44	41.92	0.163
BHM	97.69	30.04	86.54	24.47	85.75	39.04	0.417
CBHM	87.95	8.95	79.80	6.54	71.74	43.14	0.117
BMA	97.06	18.03	83.23	19.08	81.00	40.45	0.329
EXNEX	97.16	17.94	77.80	11.81	69.59	38.52	0.276
$\text{mEXNEX}_{1/13}$	96.83	14.61	78.28	11.65	76.06	42.30	0.242
mEXNEX_0	95.47	9.97	75.02	9.58	71.18	42.06	0.186

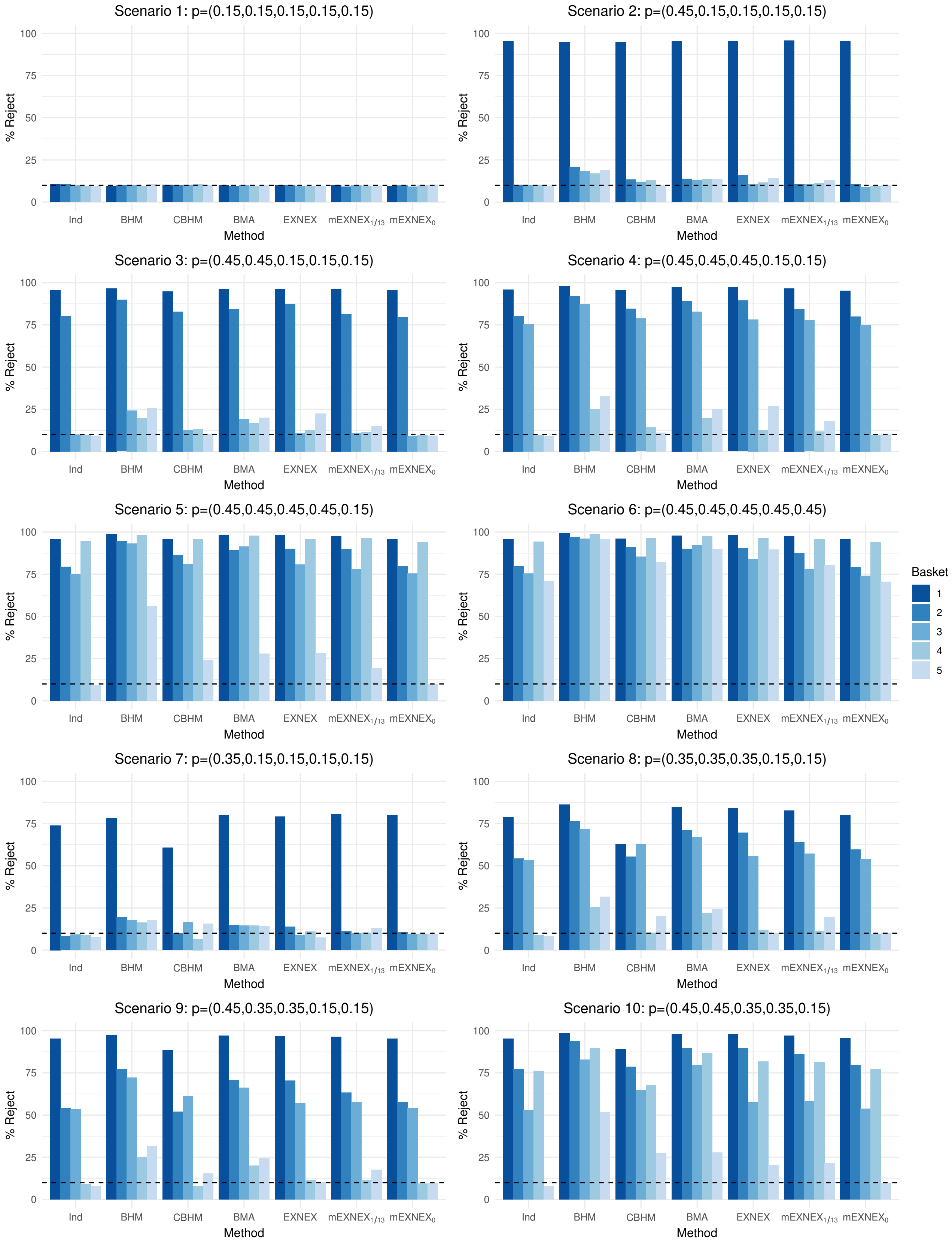

Figure S3: Percentage of rejections of the null hypothesis for each method under data scenarios 1-10, based on the realized sample sizes of 20, 10, 8, 18 and 7, with re-calibration of Δ_α to take into account unequal sample sizes.

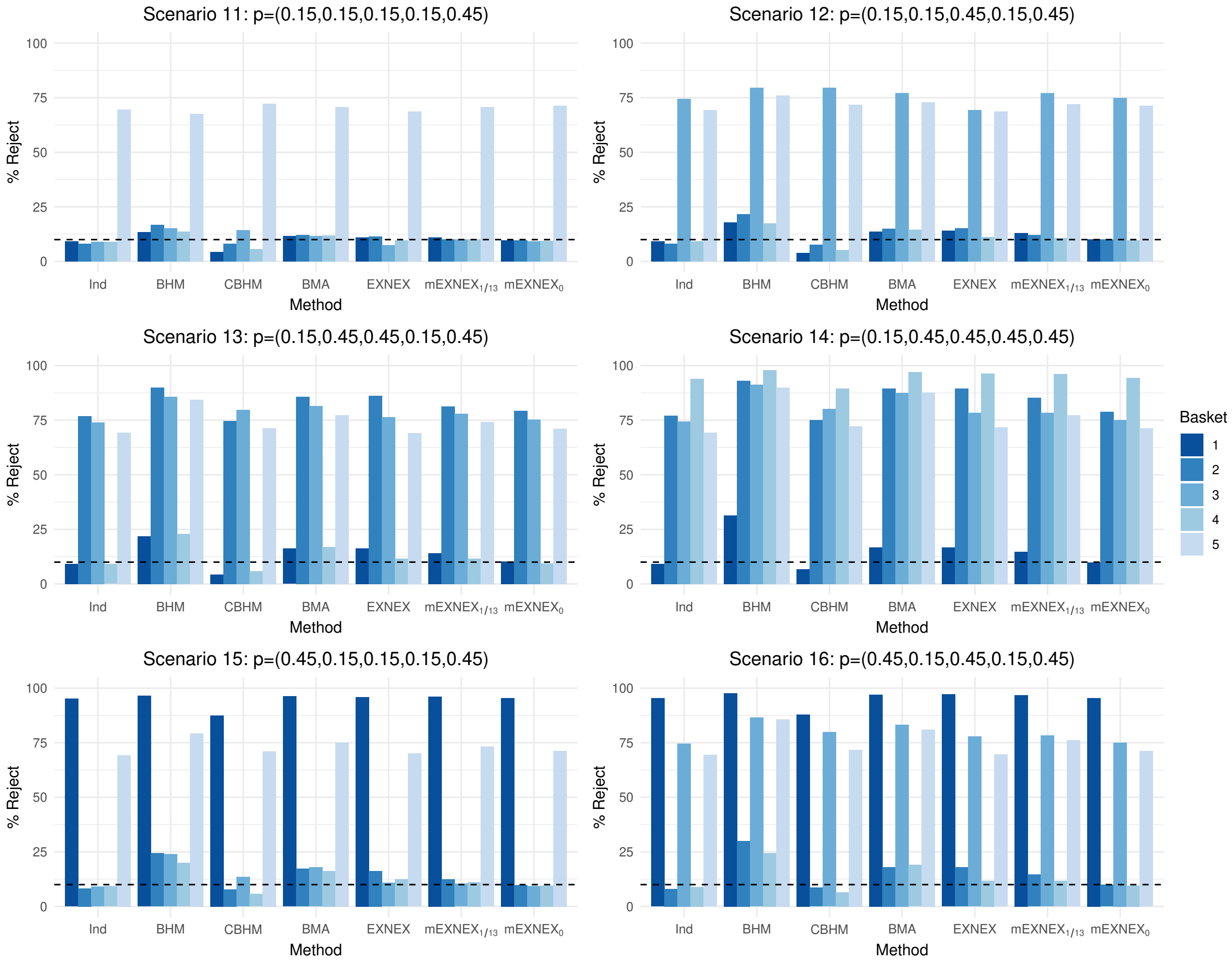

Figure S4: Percentage of rejections of the null hypothesis for each method under data scenarios 11-16, based on the realized sample sizes of 20, 10, 8, 18 and 7, with re-calibration of Δ_α to take into account unequal sample sizes.

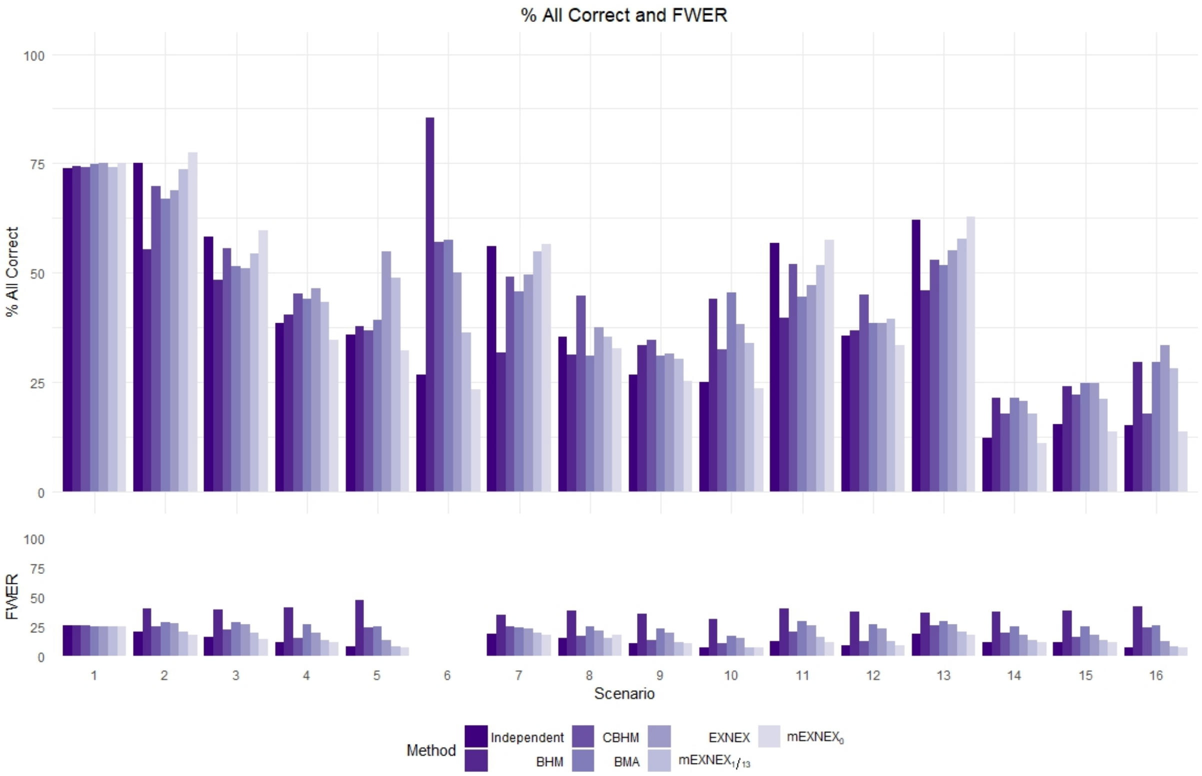

Figure S5: The family-wise error rate (FWER) and percentage of times correct inference is made across all baskets (% All Correct) for each method under each data scenario based on realized sample sizes of 20, 10, 8, 18 and 7, with re-calibration of Δ_α to take into account unequal sample sizes.

3 Estimation Ability of Information Borrowing Models

Provided in Tables S7 (planned sample size scenario), S13 (realized sample size scenario) and S11 (realized sample size scenario with re-calibrated Δ_α) are the mean posterior point estimates of p_k across the 10,000 simulations in the simulation study. In brackets are the standard deviation of these mean estimates.

Table S6: Mean point estimates of p_k across the simulations (standard deviations) based on a planned sample size of 13 per basket under scenarios 1-6.

	Mean Point Estimate (standard deviation)				
Sample Size	13	13	13	13	13
**Scenario 1**	**0.15**	**0.15**	**0.15**	**0.15**	**0.15**
Independent	0.151 (0.097)	0.150 (0.096)	0.151 (0.098)	0.151 (0.098)	0.153 (0.098)
BHM	0.150 (0.067)	0.149 (0.066)	0.149 (0.068)	0.150 (0.067)	0.151 (0.068)
CBHM	0.149 (0.084)	0.149 (0.085)	0.149 (0.084)	0.151 (0.086)	0.149 (0.085)
BMA	0.165 (0.074)	0.164 (0.073)	0.164 (0.074)	0.163 (0.074)	0.164 (0.075)
EXNEX	0.160 (0.079)	0.159 (0.079)	0.159 (0.080)	0.157 (0.078)	0.159 (0.089)
$\text{mEXNEX}_{1/13}$	0.157 (0.083)	0.157 (0.082)	0.156 (0.083)	0.156 (0.082)	0.154 (0.081)
mEXNEX_0	0.160 (0.090)	0.160 (0.089)	0.160 (0.089)	0.161 (0.090)	0.159 (0.079)
**Scenario 2**	**0.45**	**0.15**	**0.15**	**0.15**	**0.15**
Independent	0.459 (0.137)	0.150 (0.097)	0.151 (0.098)	0.151 (0.098)	0.151 (0.097)
BHM	0.381 (0.132)	0.167 (0.077)	0.167 (0.078)	0.169 (0.078)	0.167 (0.078)
CBHM	0.441 (0.147)	0.152 (0.095)	0.154 (0.096)	0.154 (0.096)	0.150 (0.095)
BMA	0.418 (0.137)	0.172 (0.079)	0.174 (0.079)	0.174 (0.079)	0.172 (0.078)
EXNEX	0.422 (0.132)	0.164 (0.081)	0.166 (0.082)	0.166 (0.082)	0.164 (0.082)
$\text{mEXNEX}_{1/13}$	0.432 (0.135)	0.158 (0.084)	0.156 (0.083)	0.158 (0.084)	0.157 (0.084)
mEXNEX_0	0.442 (0.132)	0.161 (0.091)	0.160 (0.090)	0.161 (0.090)	0.161 (0.089)
**Scenario 3**	**0.45**	**0.45**	**0.15**	**0.15**	**0.15**
Independent	0.446 (0.137)	0.452 (0.138)	0.150 (0.096)	0.151 (0.098)	0.151 (0.098)
BHM	0.404 (0.124)	0.402 (0.124)	0.180 (0.084)	0.182 (0.084)	0.182 (0.084)
CBHM	0.445 (0.142)	0.445 (0.142)	0.154 (0.099)	0.152 (0.098)	0.154 (0.099)
BMA	0.418 (0.127)	0.420 (0.129)	0.185 (0.087)	0.186 (0.088)	0.185 (0.087)
EXNEX	0.425 (0.127)	0.424 (0.129)	0.175 (0.086)	0.175 (0.085)	0.173 (0.086)
$\text{mEXNEX}_{1/13}$	0.432 (0.134)	0.430 (0.134)	0.168 (0.088)	0.166 (0.088)	0.167 (0.088)
mEXNEX_0	0.443 (0.130)	0.431 (0.129)	0.163 (0.089)	0.165 (0.089)	0.164 (0.090)
**Scenario 4**	**0.45**	**0.45**	**0.45**	**0.15**	**0.15**
Independent	0.449 (0.138)	0.449 (0.138)	0.448 (0.138)	0.152 (0.098)	0.153 (0.098)
BHM	0.415 (0.117)	0.416 (0.116)	0.416 (0.117)	0.200 (0.090)	0.203 (0.088)
CBHM	0.447 (0.139)	0.445 (0.137)	0.444 (0.137)	0.155 (0.101)	0.157 (0.102)
BMA	0.431 (0.117)	0.428 (0.116)	0.429 (0.117)	0.196 (0.096)	0.197 (0.096)
EXNEX	0.433 (0.123)	0.431 (0.120)	0.430 (0.123)	0.183 (0.091)	0.184 (0.090)
$\text{mEXNEX}_{1/13}$	0.437 (0.130)	0.437 (0.129)	0.436 (0.130)	0.177 (0.092)	0.176 (0.090)
mEXNEX_0	0.443 (0.128)	0.442 (0.130)	0.444 (0.130)	0.171 (0.089)	0.170 (0.088)
**Scenario 5**	**0.45**	**0.45**	**0.45**	**0.45**	**0.15**
Independent	0.449 (0.137)	0.448 (0.138)	0.449 (0.138)	0.450 (0.137)	0.152 (0.098)
BHM	0.428 (0.106)	0.427 (0.107)	0.427 (0.107)	0.427 (0.106)	0.238 (0.097)
CBHM	0.446 (0.135)	0.445 (0.135)	0.445 (0.135)	0.447 (0.137)	0.164 (0.112)
BMA	0.440 (0.108)	0.443 (0.108)	0.443 (0.108)	0.441 (0.107)	0.203 (0.102)
EXNEX	0.439 (0.116)	0.440 (0.116)	0.439 (0.117)	0.441 (0.114)	0.193 (0.095)
$\text{mEXNEX}_{1/13}$	0.444 (0.124)	0.443 (0.124)	0.444 (0.124)	0.446 (0.126)	0.180 (0.092)
mEXNEX_0	0.444 (0.129)	0.446 (0.130)	0.446 (0.128)	0.446 (0.129)	0.173 (0.088)
**Scenario 6**	**0.45**	**0.45**	**0.45**	**0.45**	**0.45**
Independent	0.447 (0.138)	0.450 (0.138)	0.448 (0.138)	0.450 (0.137)	0.450 (0.139)
BHM	0.451 (0.088)	0.450 (0.089)	0.451 (0.091)	0.449 (0.089)	0.449 (0.089)
CBHM	0.449 (0.124)	0.450 (0.123)	0.451 (0.122)	0.450 (0.123)	0.450 (0.123)
BMA	0.450 (0.104)	0.449 (0.103)	0.449 (0.100)	0.450 (0.104)	0.448 (0.103)
EXNEX	0.449 (0.107)	0.450 (0.109)	0.448 (0.110)	0.450 (0.110)	0.449 (0.109)
$\text{mEXNEX}_{1/13}$	0.448 (0.118)	0.448 (0.120)	0.450 (0.120)	0.447 (0.119)	0.446 (0.119)
mEXNEX_0	0.444 (0.127)	0.447 (0.128)	0.444 (0.127)	0.448 (0.126)	0.448 (0.127)

Table S7: Mean point estimates of p_k across the simulations (standard deviations) based on a planned sample size of 13 per basket under scenarios 7-10.

	Mean Point Estimate (standard deviation)				
Sample Size	13	13	13	13	13
**Scenario 7**	**0.35**	**0.15**	**0.15**	**0.15**	**0.15**
Independent	0.350 (0.131)	0.159 (0.098)	0.151 (0.096)	0.151 (0.096)	0.151 (0.097)
BHM	0.294 (0.112)	0.164 (0.072)	0.164 (0.072)	0.164 (0.071)	0.164 (0.072)
CBHM	0.335 (0.138)	0.153 (0.092)	0.154 (0.091)	0.154 (0.090)	0.154 (0.092)
BMA	0.324 (0.119)	0.171 (0.077)	0.172 (0.076)	0.172 (0.076)	0.171 (0.076)
EXNEX	0.329 (0.120)	0.164 (0.081)	0.165 (0.080)	0.165 (0.079)	0.165 (0.080)
$\text{mEXNEX}_{1/13}$	0.337 (0.129)	0.150 (0.083)	0.159 (0.082)	0.159 (0.082)	0.159 (0.083)
mEXNEX_0	0.348 (0.126)	0.161 (0.090)	0.161 (0.089)	0.162 (0.088)	0.161 (0.090)
**Scenario 8**	**0.35**	**0.35**	**0.35**	**0.15**	**0.15**
Independent	0.350 (0.131)	0.348 (0.132)	0.350 (0.131)	0.151 (0.096)	0.151 (0.097)
BHM	0.320 (0.102)	0.319 (0.103)	0.320 (0.101)	0.195 (0.078)	0.195 (0.079)
CBHM	0.344 (0.130)	0.342 (0.131)	0.344 (0.129)	0.160 (0.098)	0.160 (0.099)
BMA	0.337 (0.106)	0.335 (0.107)	0.336 (0.106)	0.192 (0.085)	0.192 (0.086)
EXNEX	0.338 (0.113)	0.336 (0.114)	0.337 (0.113)	0.181 (0.084)	0.180 (0.084)
$\text{mEXNEX}_{1/13}$	0.340 (0.121)	0.338 (0.122)	0.340 (0.121)	0.176 (0.085)	0.175 (0.086)
mEXNEX_0	0.348 (0.123)	0.346 (0.124)	0.348 (0.123)	0.169 (0.086)	0.169 (0.087)
**Scenario 9**	**0.45**	**0.35**	**0.35**	**0.15**	**0.15**
Independent	0.449 (0.137)	0.348 (0.132)	0.350 (0.131)	0.151 (0.096)	0.151 (0.097)
BHM	0.399 (0.117)	0.328 (0.104)	0.329 (0.103)	0.197 (0.082)	0.197 (0.083)
CBHM	0.443 (0.141)	0.345 (0.131)	0.347 (0.129)	0.158 (0.099)	0.157 (0.100)
BMA	0.420 (0.121)	0.340 (0.108)	0.342 (0.107)	0.194 (0.088)	0.193 (0.089)
EXNEX	0.425 (0.124)	0.340 (0.114)	0.341 (0.113)	0.182 (0.086)	0.181 (0.086)
$\text{mEXNEX}_{1/13}$	0.434 (0.132)	0.340 (0.122)	0.342 (0.121)	0.176 (0.087)	0.175 (0.088)
mEXNEX_0	0.442 (0.130)	0.347 (0.124)	0.349 (0.123)	0.169 (0.087)	0.169 (0.088)
**Scenario 10**	**0.45**	**0.45**	**0.35**	**0.35**	**0.15**
Independent	0.449 (0.137)	0.448 (0.138)	0.350 (0.131)	0.350 (0.130)	0.151 (0.097)
BHM	0.413 (0.107)	0.412 (0.108)	0.348 (0.098)	0.349 (0.096)	0.227 (0.090)
CBHM	0.444 (0.137)	0.442 (0.138)	0.349 (0.096)	0.350 (0.126)	0.164 (0.109)
BMA	0.429 (0.111)	0.438 (0.111)	0.359 (0.103)	0.359 (0.101)	0.200 (0.096)
EXNEX	0.432 (0.117)	0.430 (0.118)	0.352 (0.110)	0.353 (0.108)	0.191 (0.091)
$\text{mEXNEX}_{1/13}$	0.438 (0.126)	0.437 (0.127)	0.353 (0.117)	0.353 (0.116)	0.180 (0.091)
mEXNEX_0	0.444 (0.129)	0.442 (0.130)	0.352 (0.122)	0.352 (0.121)	0.172 (0.087)

Table S8: Mean point estimates of p_k across the simulations (standard deviations) based on realized sample sizes of 20, 10, 8, 18 and 7 patients across the 5 baskets for scenarios 1-6.

	Mean Point Estimate (standard deviation)				
Sample Size	20	10	8	18	7
**Scenario 1**	**0.15**	**0.15**	**0.15**	**0.15**	**0.15**
Independent	0.151 (0.078)	0.154 (0.110)	0.152 (0.123)	0.151 (0.083)	0.153 (0.131)
BHM	0.149 (0.061)	0.150 (0.074)	0.151 (0.077)	0.149 (0.064)	0.151 (0.079)
CBHM	0.148 (0.071)	0.152 (0.094)	0.153 (0.101)	0.149 (0.075)	0.151 (0.079)
BMA	0.160 (0.065)	0.167 (0.082)	0.171 (0.088)	0.161 (0.067)	0.174 (0.094)
EXNEX	0.156 (0.068)	0.161 (0.087)	0.166 (0.096)	0.157 (0.072)	0.167 (0.098)
$\text{mEXNEX}_{1/13}$	0.153 (0.072)	0.160 (0.096)	0.166 (0.106)	0.153 (0.076)	0.168 (0.112)
mEXNEX_0	0.162 (0.074)	0.169 (0.103)	0.178 (0.112)	0.165 (0.078)	0.183 (0.120)
**Scenario 2**	**0.45**	**0.15**	**0.15**	**0.15**	**0.15**
Independent	0.449 (0.111)	0.151 (0.111)	0.154 (0.124)	0.151 (0.083)	0.152 (0.131)
BHM	0.408 (0.110)	0.172 (0.090)	0.173 (0.096)	0.165 (0.075)	0.175 (0.099)
CBHM	0.445 (0.119)	0.153 (0.110)	0.153 (0.120)	0.153 (0.084)	0.155 (0.129)
BMA	0.427 (0.112)	0.178 (0.090)	0.183 (0.096)	0.170 (0.075)	0.185 (0.098)
EXNEX	0.432 (0.109)	0.170 (0.096)	0.173 (0.101)	0.164 (0.077)	0.177 (0.105)
$\text{mEXNEX}_{1/13}$	0.440 (0.109)	0.163 (0.100)	0.167 (0.109)	0.158 (0.079)	0.171 (0.116)
mEXNEX_0	0.446 (0.106)	0.172 (0.102)	0.179 (0.115)	0.164 (0.078)	0.177 (0.105)
**Scenario 3**	**0.45**	**0.45**	**0.15**	**0.15**	**0.15**
Independent	0.448 (0.111)	0.450 (0.157)	0.154 (0.124)	0.151 (0.082)	0.155 (0.132)
BHM	0.415 (0.105)	0.403 (0.134)	0.190 (0.099)	0.178 (0.079)	0.194 (0.103)
CBHM	0.446 (0.114)	0.443 (0.159)	0.155 (0.124)	0.151 (0.084)	0.157 (0.131)
BMA	0.429 (0.107)	0.418 (0.137)	0.198 (0.105)	0.179 (0.081)	0.204 (0.107)
EXNEX	0.434 (0.107)	0.422 (0.140)	0.184 (0.105)	0.169 (0.078)	0.188 (0.109)
$\text{mEXNEX}_{1/13}$	0.443 (0.108)	0.437 (0.145)	0.175 (0.111)	0.163 (0.079)	0.179 (0.116)
mEXNEX_0	0.447 (0.106)	0.445 (0.142)	0.180 (0.110)	0.167 (0.078)	0.186 (0.116)
**Scenario 4**	**0.45**	**0.45**	**0.45**	**0.15**	**0.15**
Independent	0.450 (0.110)	0.451 (0.156)	0.447 (0.176)	0.149 (0.083)	0.155 (0.129)
BHM	0.424 (0.101)	0.414 (0.128)	0.410 (0.137)	0.192 (0.084)	0.218 (0.104)
CBHM	0.447 (0.113)	0.445 (0.157)	0.443 (0.176)	0.155 (0.088)	0.157 (0.132)
BMA	0.433 (0.101)	0.428 (0.127)	0.425 (0.137)	0.185 (0.085)	0.217 (0.113)
EXNEX	0.435 (0.105)	0.430 (0.135)	0.426 (0.145)	0.176 (0.081)	0.201 (0.110)
$\text{mEXNEX}_{1/13}$	0.444 (0.109)	0.442 (0.145)	0.440 (0.159)	0.165 (0.078)	0.190 (0.114)
mEXNEX_0	0.446 (0.107)	0.443 (0.142)	0.443 (0.154)	0.166 (0.077)	0.191 (0.109)
**Scenario 5**	**0.45**	**0.45**	**0.45**	**0.45**	**0.15**
Independent	0.448 (0.112)	0.448 (0.157)	0.448 (0.175)	0.449 (0.117)	0.153 (0.131)
BHM	0.436 (0.091)	0.432 (0.111)	0.429 (0.117)	0.435 (0.094)	0.277 (0.106)
CBHM	0.445 (0.108)	0.446 (0.151)	0.444 (0.167)	0.444 (0.114)	0.187 (0.152)
BMA	0.443 (0.091)	0.443 (0.114)	0.443 (0.124)	0.446 (0.095)	0.226 (0.123)
EXNEX	0.445 (0.098)	0.440 (0.127)	0.437 (0.135)	0.445 (0.101)	0.219 (0.116)
$\text{mEXNEX}_{1/13}$	0.448 (0.106)	0.446 (0.139)	0.445 (0.153)	0.448 (0.109)	0.198 (0.113)
mEXNEX_0	0.445 (0.106)	0.444 (0.143)	0.441 (0.154)	0.445 (0.111)	0.190 (0.106)
**Scenario 6**	**0.45**	**0.45**	**0.45**	**0.45**	**0.45**
Independent	0.450 (0.110)	0.449 (0.158)	0.449 (0.175)	0.450 (0.116)	0.447 (0.184)
BHM	0.451 (0.083)	0.449 (0.099)	0.449 (0.103)	0.450 (0.086)	0.451 (0.105)
CBHM	0.450 (0.104)	0.448 (0.138)	0.448 (0.154)	0.451 (0.107)	0.451 (0.161)
BMA	0.450 (0.090)	0.451 (0.113)	0.449 (0.124)	0.449 (0.091)	0.448 (0.127)
EXNEX	0.451 (0.094)	0.449 (0.121)	0.445 (0.129)	0.449 (0.098)	0.448 (0.134)
$\text{mEXNEX}_{1/13}$	0.446 (0.104)	0.448 (0.138)	0.446 (0.149)	0.449 (0.091)	0.448 (0.127)
mEXNEX_0	0.447 (0.106)	0.443 (0.141)	0.444 (0.154)	0.445 (0.112)	0.442 (0.161)

Table S9: Mean point estimates of p_k across the simulations (standard deviations) based on realized sample sizes of 20, 10, 8, 18 and 7 patients across the 5 baskets for scenarios 7-12.

	Mean Point Estimate (standard deviation)				
Sample Size	20	10	8	18	7
**Scenario 7**	**0.35**	**0.15**	**0.15**	**0.15**	**0.15**
Independent	0.350 (0.106)	0.151 (0.111)	0.153 (0.121)	0.151 (0.082)	0.154 (0.130)
BHM	0.312 (0.098)	0.168 (0.083)	0.171 (0.086)	0.165 (0.069)	0.172 (0.090)
CBHM	0.339 (0.113)	0.155 (0.105)	0.158 (0.113)	0.154 (0.080)	0.160 (0.121)
BMA	0.330 (0.100)	0.178 (0.087)	0.183 (0.090)	0.172 (0.072)	0.185 (0.095)
EXNEX	0.336 (0.102)	0.169 (0.092)	0.173 (0.097)	0.163 (0.073)	0.176 (0.102)
$\text{mEXNEX}_{1/13}$	0.344 (0.106)	0.163 (0.098)	0.167 (0.106)	0.158 (0.076)	0.171 (0.113)
mEXNEX_0	0.351 (0.102)	0.173 (0.103)	0.178 (0.112)	0.165 (0.076)	0.181 (0.120)
**Scenario 8**	**0.35**	**0.35**	**0.35**	**0.15**	**0.15**
Independent	0.350 (0.106)	0.347 (0.150)	0.349 (0.165)	0.151 (0.082)	0.154 (0.130)
BHM	0.326 (0.091)	0.138 (0.112)	0.316 (0.118)	0.188 (0.074)	0.206 (0.089)
CBHM	0.344 (0.107)	0.341 (0.147)	0.342 (0.160)	0.158 (0.086)	0.167 (0.126)
BMA	0.339 (0.094)	0.336 (0.117)	0.337 (0.125)	0.185 (0.078)	0.206 (0.101)
EXNEX	0.340 (0.097)	0.336 (0.125)	0.336 (0.134)	0.174 (0.075)	0.194 (0.102)
$\text{mEXNEX}_{1/13}$	0.346 (0.103)	0.344 (0.137)	0.345 (0.148)	0.166 (0.076)	0.186 (0.109)
mEXNEX_0	0.351 (0.102)	0.351 (0.136)	0.354 (0.146)	0.166 (0.075)	0.190 (0.109)
**Scenario 9**	**0.45**	**0.35**	**0.35**	**0.15**	**0.15**
Independent	0.450 (0.111)	0.347 (0.150)	0.349 (0.165)	0.151 (0.082)	0.154 (0.130)
BHM	0.413 (0.103)	0.329 (0.115)	0.328 (0.122)	0.189 (0.078)	0.209 (0.097)
CBHM	0.445 (0.115)	0.349 (0.148)	0.347 (0.162)	0.155 (0.086)	0.161 (0.131)
BMA	0.428 (0.104)	0.344 (0.119)	0.345 (0.126)	0.185 (0.081)	0.210 (0.106)
EXNEX	0.433 (0.105)	0.341 (0.127)	0.342 (0.135)	0.174 (0.077)	0.196 (0.106)
$\text{mEXNEX}_{1/13}$	0.442 (0.109)	0.345 (0.138)	0.347 (0.149)	0.165 (0.077)	0.186 (0.110)
mEXNEX_0	0.446 (0.106)	0.352 (0.136)	0.354 (0.146)	0.166 (0.075)	0.190 (0.109)
**Scenario 10**	**0.45**	**0.45**	**0.35**	**0.35**	**0.15**
Independent	0.450 (0.111)	0.446 (0.157)	0.349 (0.165)	0.350 (0.110)	0.154 (0.130)
BHM	0.423 (0.092)	0.414 (0.113)	0.355 (0.111)	0.355 (0.087)	0.258 (0.100)
CBHM	0.443 (0.111)	0.438 (0.152)	0.351 (0.155)	0.350 (0.106)	0.185 (0.142)
BMA	0.434 (0.093)	0.429 (0.118)	0.365 (0.119)	0.363 (0.090)	0.223 (0.115)
EXNEX	0.437 (0.099)	0.429 (0.128)	0.356 (0.129)	0.355 (0.097)	0.212 (0.110)
$\text{mEXNEX}_{1/13}$	0.444 (0.106)	0.439 (0.142)	0.356 (0.144)	0.353 (0.104)	0.194 (0.110)
mEXNEX_0	0.446 (0.106)	0.442 (0.142)	0.355 (0.146)	0.352 (0.104)	0.191 (0.108)
**Scenario 11**	**0.15**	**0.15**	**0.15**	**0.15**	**0.45**
Independent	0.150 (0.079)	0.151 (0.111)	0.153 (0.121)	0.151 (0.082)	0.449 (0.186)
BHM	0.160 (0.066)	0.165 (0.081)	0.168 (0.084)	0.161 (0.067)	0.352 (0.161)
CBHM	0.152 (0.077)	0.154 (0.105)	0.157 (0.114)	0.152 (0.079)	0.429 (0.201)
BMA	0.165 (0.066)	0.172 (0.083)	0.177 (0.087)	0.166 (0.068)	0.406 (0.170)
EXNEX	0.160 (0.069)	0.167 (0.089)	0.171 (0.094)	0.161 (0.071)	0.408 (0.166)
$\text{mEXNEX}_{1/13}$	0.155 (0.073)	0.162 (0.096)	0.167 (0.103)	0.156 (0.075)	0.432 (0.170)
mEXNEX_0	0.162 (0.074)	0.172 (0.101)	0.181 (0.107)	0.166 (0.076)	0.440 (0.161)
**Scenario 12**	**0.15**	**0.15**	**0.45**	**0.15**	**0.45**
Independent	0.150 (0.079)	0.151 (0.110)	0.449 (0.173)	0.151 (0.082)	0.449 (0.186)
BHM	0.170 (0.069)	0.179 (0.086)	0.381 (0.149)	0.171 (0.071)	0.378 (0.156)
CBHM	0.152 (0.079)	0.154 (0.110)	0.442 (0.179)	0.153 (0.082)	0.442 (0.192)
BMA	0.172 (0.070)	0.182 (0.088)	0.410 (0.156)	0.174 (0.072)	0.408 (0.164)
EXNEX	0.165 (0.071)	0.175 (0.091)	0.415 (0.154)	0.166 (0.075)	0.441 (0.161)
$\text{mEXNEX}_{1/13}$	0.159 (0.074)	0.169 (0.097)	0.431 (0.160)	0.160 (0.076)	0.430 (0.168)
mEXNEX_0	0.162 (0.074)	0.175 (0.097)	0.441 (0.152)	0.166 (0.075)	0.441 (0.161)

Table S10: Mean point estimates of p_k across the simulations (standard deviations) based on realized sample sizes of 20, 10, 8, 18 and 7 patients across the 5 baskets for scenarios 13-16.

	Mean Point Estimate (standard deviation)				
Sample Size	20	10	8	18	7
**Scenario 13**	**0.15**	**0.45**	**0.45**	**0.15**	**0.15**
Independent	0.150 (0.079)	0.446 (0.157)	0.449 (0.173)	0.151 (0.082)	0.449 (0.186)
BHM	0.181 (0.073)	0.402 (0.134)	0.400 (0.142)	0.184 (0.075)	0.397 (0.149)
CBHM	0.152 (0.080)	0.444 (0.159)	0.447 (0.175)	0.153 (0.084)	0.446 (0.188)
BMA	0.179 (0.076)	0.421 (0.139)	0.420 (0.147)	0.182 (0.078)	0.419 (0.154)
EXNEX	0.171 (0.074)	0.424 (0.140)	0.422 (0.149)	0.173 (0.076)	0.421 (0.157)
$\text{mEXNEX}_{1/13}$	0.163 (0.074)	0.435 (0.147)	0.436 (0.158)	0.165 (0.076)	0.434 (0.167)
mEXNEX_0	0.164 (0.072)	0.441 (0.143)	0.442 (0.152)	0.166 (0.075)	0.441 (0.160)
**Scenario 14**	**0.15**	**0.45**	**0.45**	**0.45**	**0.45**
Independent	0.150 (0.079)	0.446 (0.157)	0.449 (0.173)	0.450 (0.115)	0.449 (0.186)
BHM	0.206 (0.083)	0.421 (0.122)	0.420 (0.129)	0.429 (0.100)	0.419 (0.135)
CBHM	0.155 (0.086)	0.445 (0.156)	0.448 (0.172)	0.448 (0.116)	0.448 (0.185)
BMA	0.185 (0.084)	0.437 (0.123)	0.438 (0.130)	0.442 (0.101)	0.438 (0.137)
EXNEX	0.178 (0.078)	0.435 (0.131)	0.435 (0.140)	0.441 (0.105)	0.435 (0.147)
$\text{mEXNEX}_{1/13}$	0.166 (0.075)	0.442 (0.143)	0.444 (0.153)	0.446 (0.110)	0.443 (0.162)
mEXNEX_0	0.164 (0.072)	0.441 (0.143)	0.443 (0.152)	0.446 (0.109)	0.441 (0.160)
**Scenario 15**	**0.45**	**0.15**	**0.15**	**0.15**	**0.45**
Independent	0.450 (0.111)	0.151 (0.111)	0.153 (0.121)	0.151 (0.082)	0.149 (0.186)
BHM	0.415 (0.107)	0.184 (0.093)	0.189 (0.097)	0.176 (0.076)	0.392 (0.153)
CBHM	0.447 (0.115)	0.153 (0.112)	0.155 (0.122)	0.153 (0.083)	0.449 (0.189)
BMA	0.428 (0.109)	0.189 (0.095)	0.196 (0.099)	0.178 (0.078)	0.412 (0.156)
EXNEX	0.433 (0.107)	0.177 (0.096)	0.183 (0.102)	0.169 (0.076)	0.417 (0.159)
$\text{mEXNEX}_{1/13}$	0.442 (0.109)	0.168 (0.100)	0.175 (0.107)	0.161 (0.077)	0.431 (0.168)
mEXNEX_0	0.446 (0.106)	0.175 (0.100)	0.182 (0.107)	0.161 (0.077)	0.431 (0.158)
**Scenario 16**	**0.45**	**0.15**	**0.45**	**0.15**	**0.45**
Independent	0.450 (0.111)	0.151 (0.110)	0.449 (0.173)	0.151 (0.082)	0.449 (0.186)
BHM	0.422 (0.102)	0.202 (0.095)	0.407 (0.139)	0.189 (0.080)	0.405 (0.146)
CBHM	0.448 (0.113)	0.155 (0.114)	0.447 (0.174)	0.154 (0.085)	0.447 (0.187)
BMA	0.434 (0.104)	0.201 (0.102)	0.424 (0.138)	0.185 (0.083)	0.423 (0.146)
EXNEX	0.436 (0.105)	0.188 (0.098)	0.426 (0.146)	0.175 (0.078)	0.425 (0.154)
$\text{mEXNEX}_{1/13}$	0.443 (0.108)	0.177 (0.099)	0.437 (0.156)	0.166 (0.077)	0.436 (0.165)
mEXNEX_0	0.446 (0.106)	0.178 (0.099)	0.437 (0.156)	0.166 (0.077)	0.436 (0.165)

Table S11: Mean point estimates of p_k across the simulations (standard deviations) based on realized sample sizes of 20, 10, 8, 18 and 7 patients across the 5 baskets with re-calibration of Δ_α under scenarios 1-6.

	Mean Point Estimate (standard deviation)				
Sample Size	20	10	8	18	7
**Scenario 1**	**0.15**	**0.15**	**0.15**	**0.15**	**0.15**
Independent	0.150 (0.079)	0.153 (0.111)	0.153 (0.122)	0.150 (0.084)	0.153 (0.132)
BHM	0.149 (0.062)	0.149 (0.074)	0.151 (0.078)	0.149 (0.064)	0.151 (0.079)
CBHM	0.148 (0.071)	0.150 (0.092)	0.152 (0.099)	0.147 (0.074)	0.156 (0.107)
BMA	0.158 (0.065)	0.165 (0.081)	0.171 (0.087)	0.161 (0.068)	0.173 (0.091)
EXNEX	0.156 (0.069)	0.162 (0.089)	0.165 (0.096)	0.156 (0.071)	0.169 (0.098)
$\text{mEXNEX}_{1/13}$	0.153 (0.073)	0.160 (0.096)	0.165 (0.105)	0.155 (0.076)	0.168 (0.112)
mEXNEX_0	0.162 (0.075)	0.170 (0.104)	0.176 (0.112)	0.165 (0.078)	0.182 (0.121)
**Scenario 2**	**0.45**	**0.15**	**0.15**	**0.15**	**0.15**
Independent	0.448 (0.110)	0.152 (0.109)	0.153 (0.122)	0.150 (0.083)	0.151 (0.129)
BHM	0.409 (0.112)	0.171 (0.091)	0.172 (0.095)	0.165 (0.074)	0.175 (0.099)
CBHM	0.445 (0.118)	0.154 (0.110)	0.154 (0.119)	0.152 (0.084)	0.156 (0.129)
BMA	0.426 (0.112)	0.177 (0.089)	0.183 (0.095)	0.171 (0.075)	0.187 (0.098)
EXNEX	0.432 (0.110)	)0.172 (0.095)	0.175 (0.101)	0.163 (0.076)	0.176 (0.104)
$\text{mEXNEX}_{1/13}$	0.444 (0.111)	0.161 (0.097)	0.168 (0.111)	0.160 (0.079)	0.169 (0.113)
mEXNEX_0	0.446 (0.106)	0.175 (0.103)	0.179 (0.114)	0.164 (0.078)	0.181 (0.119)
**Scenario 3**	**0.45**	**0.45**	**0.15**	**0.15**	**0.15**
Independent	0.452 (0.113)	0.448 (0.157)	0.152 (0.123)	0.150 (0.083)	0.152 (0.128)
BHM	0.417 (0.106)	0.401 (0.136)	0.192 (0.100)	0.177 (0.078)	0.196 (0.103)
CBHM	0.448 (0.116)	0.446 (0.160)	0.156 (0.125)	0.152 (0.085)	0.156 (0.133)
BMA	0.427 (0.106)	0.416 (0.136)	0.201 (0.104)	0.181 (0.081)	0.205 (0.106)
EXNEX	0.434 (0.107)	0.425 (0.140)	0.186 (0.104)	0.169 (0.078)	0.189 (0.108)
$\text{mEXNEX}_{1/13}$	0.443 (0.107)	0.436 (0.145)	0.176 (0.109)	0.162 (0.079)	0.178 (0.115)
mEXNEX_0	0.448 (0.106)	0.440 (0.140)	0.181 (0.110)	0.167 (0.078)	0.186 (0.117)
**Scenario 4**	**0.45**	**0.45**	**0.45**	**0.15**	**0.15**
Independent	0.449 (0.112)	0.446 (0.157)	0.449 (0.176)	0.152 (0.084)	0.156 (0.132)
BHM	0.424 (0.102)	0.412 (0.129)	0.410 (0.139)	0.192 (0.084)	0.216 (0.104)
CBHM	0.448 (0.114)	0.447 (0.157)	0.445 (0.175)	0.154 (0.087)	0.159 (0.134)
BMA	0.435 (0.102)	0.431 (0.128)	0.428 (0.136)	0.187 (0.087)	0.215 (0.113)
EXNEX	0.438 (0.104)	0.429 (0.136)	0.428 (0.146)	0.176 (0.081)	0.200 (0.110)
$\text{mEXNEX}_{1/13}$	0.444 (0.109)	0.436 (0.144)	0.439 (0.157)	0.165 (0.077)	0.190 (0.112)
mEXNEX_0	0.445 (0.106)	0.445 (0.143)	0.444 (0.156)	0.165 (0.077)	0.192 (0.108)
**Scenario 5**	**0.45**	**0.45**	**0.45**	**0.45**	**0.15**
Independent	0.449 (0.110)	0.449 (0.156)	0.450 (0.173)	0.448 (0.117)	0.154 (0.130)
BHM	0.435 (0.090)	0.432 (0.112)	0.431 (0.119)	0.435 (0.092)	0.278 (0.106)
CBHM	0.446 (0.109)	0.445 (0.151)	0.443 (0.168)	0.445 (0.114)	0.187 (0.151)
BMA	0.445 (0.090)	0.444 (0.114)	0.443 (0.124)	0.444 (0.094)	0.227 (0.123)
EXNEX	0.443 (0.099)	0.439 (0.128)	0.437 (0.138)	0.442 (0.102)	0.218 (0.115)
$\text{mEXNEX}_{1/13}$	0.447 (0.106)	0.447 (0.140)	0.444 (0.151)	0.444 (0.111)	0.197 (0.112)
mEXNEX_0	0.446 (0.107)	0.445 (0.142)	0.441 (0.155)	0.446 (0.112)	0.192 (0.107)
**Scenario 6**	**0.45**	**0.45**	**0.45**	**0.45**	**0.45**
Independent	0.451 (0.110)	0.445 (0.158)	0.450 (0.175)	0.449 (0.118)	0.450 (0.187)
BHM	0.449 (0.082)	0.449 (0.097)	0.449 (0.101)	0.451 (0.085)	0.450 (0.104)
CBHM	0.449 (0.104)	0.449 (0.138)	0.450 (0.151)	0.449 (0.107)	0.452 (0.159)
BMA	0.451 (0.090)	0.451 (0.113)	0.448 (0.122)	0.449 (0.092)	0.447 (0.130)
EXNEX	0.449 (0.094)	0.445 (0.120)	0.449 (0.130)	0.447 (0.099)	0.443 (0.135)
$\text{mEXNEX}_{1/13}$	0.448 (0.104)	0.446 (0.137)	0.446 (0.148)	0.449 (0.108)	0.445 (0.158)
mEXNEX_0	0.447 (0.105)	0.440 (0.142)	0.443 (0.153)	0.446 (0.110)	0.435 (0.159)

Table S12: Mean point estimates of p_k across the simulations (standard deviations) based on realized sample sizes of 20, 10, 8, 18 and 7 patients across the 5 baskets with re-calibration of Δ_α under scenarios 7-12.

	Mean Point Estimate (standard deviation)				
Sample Size	20	10	8	18	7
**Scenario 7**	**0.35**	**0.15**	**0.15**	**0.15**	**0.15**
Independent	0.350 (0.106)	0.151 (0.111)	0.153 (0.121)	0.151 (0.082)	0.154 (0.130)
BHM	0.312 (0.098)	0.168 (0.083)	0.171 (0.086)	0.165 (0.069)	0.172 (0.090)
CBHM	0.337 (0.116)	0.153 (0.106)	0.155 (0.115)	0.152 (0.080)	0.157 (0.123)
BMA	0.330 (0.100)	0.178 (0.087)	0.183 (0.090)	0.172 (0.072)	0.185 (0.095)
EXNEX	0.336 (0.102)	0.169 (0.092)	0.173 (0.097)	0.163 (0.073)	0.176 (0.102)
$\text{mEXNEX}_{1/13}$	0.344 (0.106)	0.163 (0.098)	0.167 (0.106)	0.158 (0.076)	0.171 (0.113)
mEXNEX_0	0.351 (0.102)	0.173 (0.103)	0.178 (0.112)	0.165 (0.076)	0.181 (0.120)
**Scenario 8**	**0.35**	**0.35**	**0.35**	**0.15**	**0.15**
Independent	0.350 (0.106)	0.347 (0.150)	0.349 (0.165)	0.151 (0.082)	0.154 (0.130)
BHM	0.326 (0.091)	0.138 (0.112)	0.316 (0.118)	0.188 (0.074)	0.206 (0.089)
CBHM	0.341 (0.111)	0.338 (0.150)	0.340 (0.164)	0.156 (0.085)	0.162 (0.128)
BMA	0.339 (0.094)	0.336 (0.117)	0.337 (0.125)	0.185 (0.078)	0.206 (0.101)
EXNEX	0.340 (0.097)	0.336 (0.125)	0.336 (0.134)	0.174 (0.075)	0.194 (0.102)
$\text{mEXNEX}_{1/13}$	0.346 (0.103)	0.344 (0.137)	0.345 (0.148)	0.166 (0.076)	0.186 (0.109)
mEXNEX_0	0.351 (0.102)	0.351 (0.136)	0.354 (0.146)	0.166 (0.075)	0.190 (0.109)
**Scenario 9**	**0.45**	**0.35**	**0.35**	**0.15**	**0.15**
Independent	0.450 (0.111)	0.347 (0.150)	0.349 (0.165)	0.151 (0.082)	0.154 (0.130)
BHM	0.413 (0.103)	0.329 (0.115)	0.328 (0.122)	0.189 (0.078)	0.209 (0.097)
CBHM	0.443 (0.118)	0.344 (0.149)	0.346 (0.164)	0.154 (0.086)	0.157 (0.133)
BMA	0.428 (0.104)	0.344 (0.119)	0.345 (0.126)	0.185 (0.081)	0.210 (0.106)
EXNEX	0.433 (0.105)	0.341 (0.127)	0.342 (0.135)	0.174 (0.077)	0.196 (0.106)
$\text{mEXNEX}_{1/13}$	0.442 (0.109)	0.345 (0.138)	0.347 (0.149)	0.165 (0.077)	0.186 (0.110)
mEXNEX_0	0.446 (0.106)	0.352 (0.136)	0.354 (0.146)	0.166 (0.075)	0.190 (0.109)
**Scenario 10**	**0.45**	**0.45**	**0.35**	**0.35**	**0.15**
Independent	0.450 (0.111)	0.446 (0.157)	0.349 (0.165)	0.350 (0.110)	0.154 (0.130)
BHM	0.423 (0.092)	0.414 (0.113)	0.355 (0.111)	0.355 (0.087)	0.258 (0.100)
CBHM	0.434 (0.121)	0.431 (0.160)	0.343 (0.160)	0.343 (0.111)	0.174 (0.142)
BMA	0.434 (0.093)	0.429 (0.118)	0.365 (0.119)	0.363 (0.090)	0.223 (0.115)
EXNEX	0.437 (0.099)	0.429 (0.128)	0.356 (0.129)	0.355 (0.097)	0.212 (0.110)
$\text{mEXNEX}_{1/13}$	0.444 (0.106)	0.439 (0.142)	0.356 (0.144)	0.353 (0.104)	0.194 (0.110)
mEXNEX_0	0.446 (0.106)	0.442 (0.142)	0.355 (0.146)	0.352 (0.104)	0.191 (0.108)
**Scenario 11**	**0.15**	**0.15**	**0.15**	**0.15**	**0.45**
Independent	0.150 (0.079)	0.151 (0.111)	0.153 (0.121)	0.151 (0.082)	0.449 (0.186)
BHM	0.160 (0.066)	0.165 (0.081)	0.168 (0.084)	0.161 (0.067)	0.352 (0.161)
CBHM	0.151 (0.077)	0.152 (0.106)	0.155 (0.116)	0.151 (0.080)	0.430 (0.203)
BMA	0.165 (0.066)	0.172 (0.083)	0.177 (0.087)	0.166 (0.068)	0.406 (0.170)
EXNEX	0.160 (0.069)	0.167 (0.089)	0.171 (0.094)	0.161 (0.071)	0.408 (0.166)
$\text{mEXNEX}_{1/13}$	0.155 (0.073)	0.162 (0.096)	0.167 (0.103)	0.156 (0.075)	0.432 (0.170)
mEXNEX_0	0.162 (0.074)	0.172 (0.101)	0.181 (0.107)	0.166 (0.076)	0.440 (0.161)
**Scenario 12**	**0.15**	**0.15**	**0.45**	**0.15**	**0.45**
Independent	0.150 (0.079)	0.151 (0.110)	0.449 (0.173)	0.151 (0.082)	0.449 (0.186)
BHM	0.170 (0.069)	0.179 (0.086)	0.381 (0.149)	0.171 (0.071)	0.378 (0.156)
CBHM	0.151 (0.079)	0.152 (0.111)	0.442 (0.180)	0.152 (0.082)	0.442 (0.193)
BMA	0.172 (0.070)	0.182 (0.088)	0.410 (0.156)	0.174 (0.072)	0.408 (0.164)
EXNEX	0.165 (0.071)	0.175 (0.091)	0.415 (0.154)	0.166 (0.075)	0.441 (0.161)
$\text{mEXNEX}_{1/13}$	0.159 (0.074)	0.169 (0.097)	0.431 (0.160)	0.160 (0.076)	0.430 (0.168)
mEXNEX_0	0.162 (0.074)	0.175 (0.097)	0.441 (0.152)	0.166 (0.075)	0.441 (0.161)

Table S13: Mean point estimates of p_k across the simulations (standard deviations) based on realized sample sizes of 20, 10, 8, 18 and 7 patients across the 5 baskets with re-calibration of Δ_α under scenarios 13-16.

	Mean Point Estimate (standard deviation)				
Sample Size	20	10	8	18	7
**Scenario 13**	**0.15**	**0.45**	**0.45**	**0.15**	**0.15**
Independent	0.150 (0.079)	0.446 (0.157)	0.449 (0.173)	0.151 (0.082)	0.449 (0.186)
BHM	0.181 (0.073)	0.402 (0.134)	0.400 (0.142)	0.184 (0.075)	0.397 (0.149)
CBHM	0.152 (0.081)	0.444 (0.159)	0.447 (0.175)	0.153 (0.084)	0.446 (0.188)
BMA	0.179 (0.076)	0.421 (0.139)	0.420 (0.147)	0.182 (0.078)	0.419 (0.154)
EXNEX	0.171 (0.074)	0.424 (0.140)	0.422 (0.149)	0.173 (0.076)	0.421 (0.157)
$\text{mEXNEX}_{1/13}$	0.163 (0.074)	0.435 (0.147)	0.436 (0.158)	0.165 (0.076)	0.434 (0.167)
mEXNEX_0	0.164 (0.072)	0.441 (0.143)	0.442 (0.152)	0.166 (0.075)	0.441 (0.160)
**Scenario 14**	**0.15**	**0.45**	**0.45**	**0.45**	**0.45**
Independent	0.150 (0.079)	0.446 (0.157)	0.449 (0.173)	0.450 (0.115)	0.449 (0.186)
BHM	0.206 (0.083)	0.421 (0.122)	0.420 (0.129)	0.429 (0.100)	0.419 (0.135)
CBHM	0.154 (0.086)	0.444 (0.158)	0.446 (0.174)	0.446 (0.119)	0.447 (0.187)
BMA	0.185 (0.084)	0.437 (0.123)	0.438 (0.130)	0.442 (0.101)	0.438 (0.137)
EXNEX	0.178 (0.078)	0.435 (0.131)	0.435 (0.140)	0.441 (0.105)	0.435 (0.147)
$\text{mEXNEX}_{1/13}$	0.166 (0.075)	0.442 (0.143)	0.444 (0.153)	0.446 (0.110)	0.443 (0.162)
mEXNEX_0	0.164 (0.072)	0.441 (0.143)	0.443 (0.152)	0.446 (0.109)	0.441 (0.160)
**Scenario 15**	**0.45**	**0.15**	**0.15**	**0.15**	**0.45**
Independent	0.450 (0.111)	0.151 (0.111)	0.153 (0.121)	0.151 (0.082)	0.149 (0.186)
BHM	0.415 (0.107)	0.184 (0.093)	0.189 (0.097)	0.176 (0.076)	0.392 (0.153)
CBHM	0.446 (0.116)	0.151 (0.113)	0.153 (0.123)	0.152 (0.083)	0.445 (0.190)
BMA	0.428 (0.109)	0.189 (0.095)	0.196 (0.099)	0.178 (0.078)	0.412 (0.156)
EXNEX	0.433 (0.107)	0.177 (0.096)	0.183 (0.102)	0.169 (0.076)	0.417 (0.159)
$\text{mEXNEX}_{1/13}$	0.442 (0.109)	0.168 (0.100)	0.175 (0.107)	0.161 (0.077)	0.431 (0.168)
mEXNEX_0	0.446 (0.106)	0.175 (0.100)	0.182 (0.107)	0.161 (0.077)	0.431 (0.158)
**Scenario 16**	**0.45**	**0.15**	**0.45**	**0.15**	**0.45**
Independent	0.450 (0.111)	0.151 (0.110)	0.449 (0.173)	0.151 (0.082)	0.449 (0.186)
BHM	0.422 (0.102)	0.202 (0.095)	0.407 (0.139)	0.189 (0.080)	0.405 (0.146)
CBHM	0.447 (0.114)	0.153 (0.114)	0.447 (0.175)	0.153 (0.085)	0.447 (0.188)
BMA	0.434 (0.104)	0.201 (0.102)	0.424 (0.138)	0.185 (0.083)	0.423 (0.146)
EXNEX	0.436 (0.105)	0.188 (0.098)	0.426 (0.146)	0.175 (0.078)	0.425 (0.154)
$\text{mEXNEX}_{1/13}$	0.443 (0.108)	0.177 (0.099)	0.437 (0.156)	0.166 (0.077)	0.436 (0.165)
mEXNEX_0	0.446 (0.106)	0.178 (0.099)	0.437 (0.156)	0.166 (0.077)	0.436 (0.165)

4 Simulation Study in Which the Truth Vector, p, is Varied

There are an infinite number of data scenarios one could fall in when conducting clinical trial analysis, the results presented in the previously mentioned simulation studies are only a subset of these feasible possible data scenarios. The data scenarios used in the simulation studies were selected to cover a wide range of cases, however, some important cases may have not been investigated.

To overcome this, a further simulation study was conducted within which, rather than fixing the true probability of success parameter prior to the study, for every simulation run a new random truth vector, p, was generated and data simulated from a Binomial distribution using these p values. In order to ensure equal chances of lying in the null and non-null case, p was selected with uniform probability across the ranges [0,0.15] and [0.35,0.5].

A total of 20,000 simulations for each borrowing method was run under the three simulation cases: planned sample size of $n_k = 13$ in each basket, realized sample sizes of 20, 10, 8, 18 and 7 without re-calibration of Δ_α and the realized sample size case with re-calibration. For each method and setting, we find the following operating characteristics:

- Type I error rate - the percentage of times the null was rejected out of the cases where the null was in fact

true. This is computed for each basket.

- Power - the percentage of times the null was rejected out of the cases where the true response rate was non-null. This is also computed for each basket.
- Percentage of all correct inference (% Correct) - the percentage of times the correct decision regarding whether to accept/reject the null was made across all 5 baskets.
- Family-wise error rate (FWER) - the percentage of times at least one type I error was made across the 5 baskets (excluding the global alternative cases where p_k is non-null in all k baskets.

4.1 Planned Sample Size

Figure S6 presents results for the planned sample size case in which 13 patients were observed in each of the 5 baskets. The top section of the figure demonstrates the type I error rate under each of the 7 methods. Similar to results presented in the planned sample size simulation in the main manuscript, the BHM and BMA have the highest error rates at approximately 5.0% and 4.51% respectively. All methods have errors less than or equal to the nominal 10% level. The reduced error rates comes from, in some cases, the true response rate lying well below the null level of 15%. The cut-off value Δ_α was calibrated for each method under a null scenario where the true response rate is 0.15. When a basket has a true response rate less than 0.15, the Δ_α value becomes conservative as it is easier to correctly identify that the treatment is ineffective. Under each of the borrowing models there is some degree of pull towards the common mean, which is most evident in the BHM and BMA case, and thus all have a higher error rate than under an independent analysis. Both the standard EXNEX and $\text{mEXNEX}_{1/13}$ model have almost identical rates both at around 3.2% each, whilst the mEXNEX_0 has a lower error rate of 2.7%.

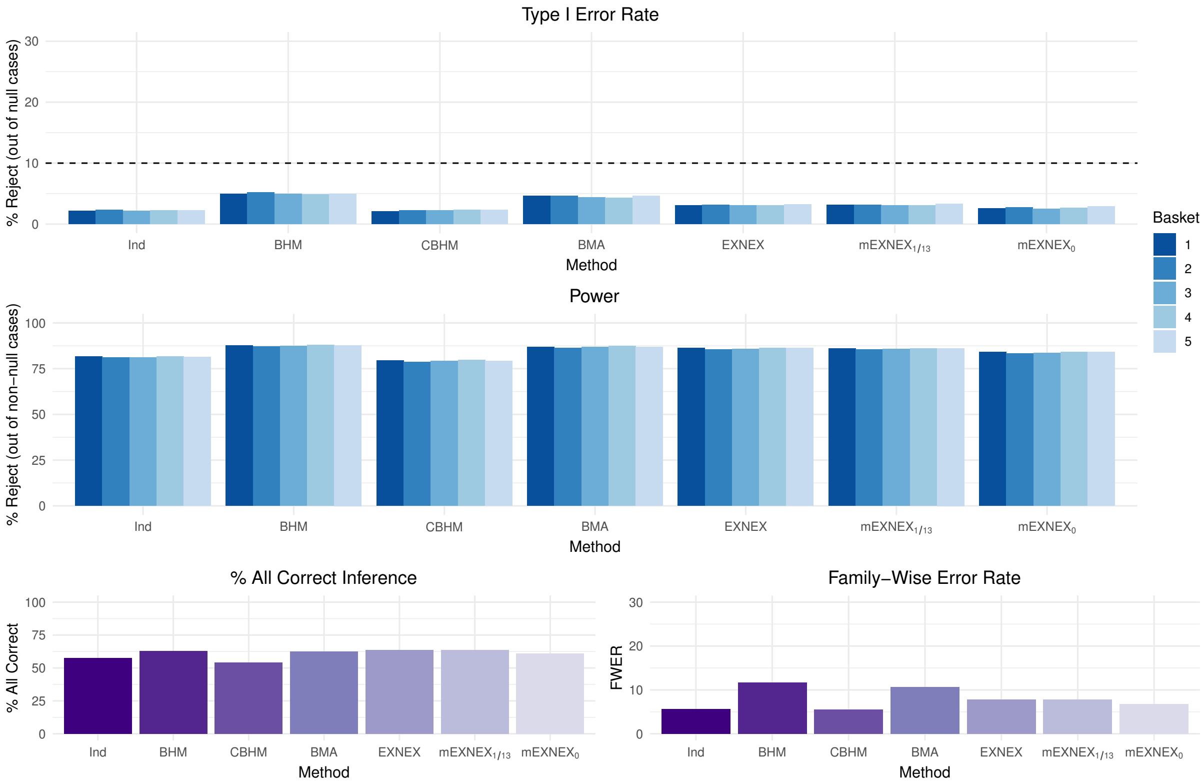

Figure S6: Operating characteristics under varied truths for the planned sample size case where 13 patients are observed in each basket.

When considering power, those methods with higher error rates also demonstrate the greatest power. The BHM has a power value increased 6% to that of the independent model but that came with the inflation of error as mentioned above. Again both the EXNEX model and $\text{mEXNEX}_{1/13}$ model perform almost identically with a power of 86%. The mEXNEX_0 model has lower power at 83.9% but this is still a 2% improvement over the independent model. The CBHM has very similar power and error rate to the independent analysis.

The percentage of times correct conclusion was made across all 5 baskets is presented in the bottom left of Figure S6. The BMA approach alongside the BHM, EXNEX and mEXNEX$_{1/13}$ model all make the correct conclusion across baskets in 63% of the simulations. However, the modified EXNEX approach with $c = 0$ makes slightly fewer all correction conclusions at 61.2% but this is still greater than an independent analysis which has a value of 57.6%.

A more substantial difference in methods is observed when looking at the family-wise error rate. Methods that demonstrated lower type I error rate also present lower FWERs, with the independent analysis giving the lowest error alongside the CBHM. This must be weighed up with the lower percentage of all correct inference and power that these two methods possess. The BHM and BMA have much larger FWER values, as expected based on the inflated type I error rate.

To summarise, in the planned sample size case when the true response rate is varied, the BHM and BMA continue to display undesirable error rates whilst the independent analysis and CBHM lack power. The modified EXNEX model with $c = 1/13$ performs almost identically to the standard EXNEX model, whereas, when a more conservative cut-off value $c = 0$ is implemented, error rates are reduced by 0.5% from the standard EXNEX model but with a 2.1% reduction in power (but still a 2.4% improvement over an independent analysis).

4.2 Realized Sample Size

In the realized sample size case, basket sample sizes are equal to 20, 10, 8, 18 and 7 and Δ_α is calibrated based on the planned sample size of $n_k = 13$ in each of the k baskets. Results are presented in Figure S7. Similar error rates are observed as in the planned sample size case, with the BHM and BMA approach having inflated error rates with higher errors in baskets where the sample size is small. In this case the mEXNEX$_0$ model performs almost identically to an independent analysis due to the discreetness of the data making it impossible for a basket to not be analysed as independent in first step of the mEXNEX$_c$ procedure.

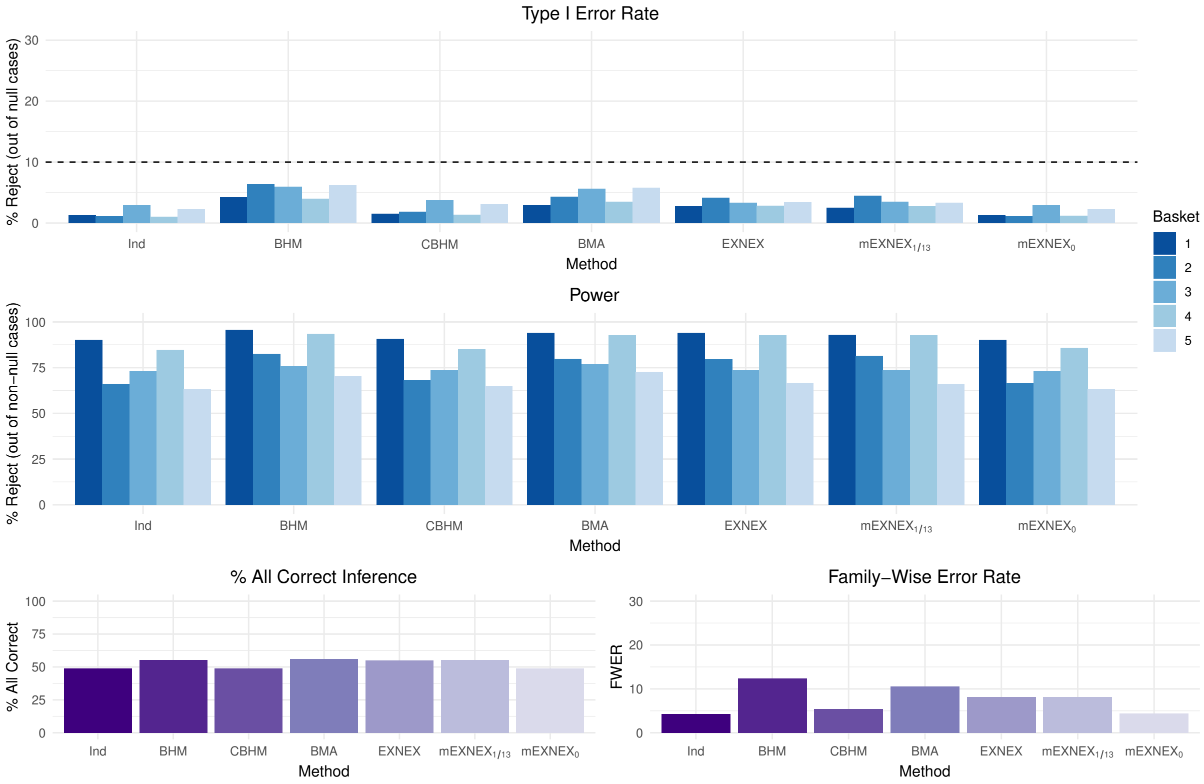

Figure S7: Operating characteristics under varied truths for the realized sample size case where 20, 10, 8, 18 and 7 patients are observed in the 5 baskets and Δ_α is not re-calibrated

The EXNEX and mEXNEX$_{1/13}$ again behave similarly in all metrics, thus little would be gained by using the modified EXNEX approach in this case (particularly for the choice of c made). The mEXNEX$_{1/13}$ does generate a 0.3% higher probability of all correct inference across the baskets compared to the EXNEX model whilst giving the same FWER.

Looking at the percentage of all correct inference across the 5 baskets and the family-wise error rates on a whole, performance of methods are very similar to the planned sample size case but with uniformly lower values for the first metric. The exception is the mEXNEX$_0$ model, which now is identical to the independent approach for the aforementioned reasons.

Overall, we conclude from these results that again a cut-off of $c = 0$ is not appropriate in the realized sample size case, however, performance when $c = 1/13$ is selected produces very similar results to the standard EXNEX model. Other values of c could be beneficial here but this was calibrated based on the planned sample size case. Power for smaller basket sizes tend to be considerably lower, so methods that borrow more strongly show clear benefits in power improvement. For example, the BHM improves power over an independent analysis by 7.2% in basket 5 when the sample size is just 7. The mEXNEX$_{1/13}$ model also demonstrates power improvement over an independent analysis by 2.8%.

4.3 Realized Sample Size with Re-Calibrated Δ_α

The above took the decision cut-off value Δ_α calibrated based on the planned sample size of 13 patients in each basket applied to the realized sample sizes of 20, 10, 8, 18 and 7 for the 5 baskets. In this section these Δ_α values are re-calibrated based on the realized sample sizes. Results are akin to the realized sample size without re-calibration case and hence discussion is omitted.

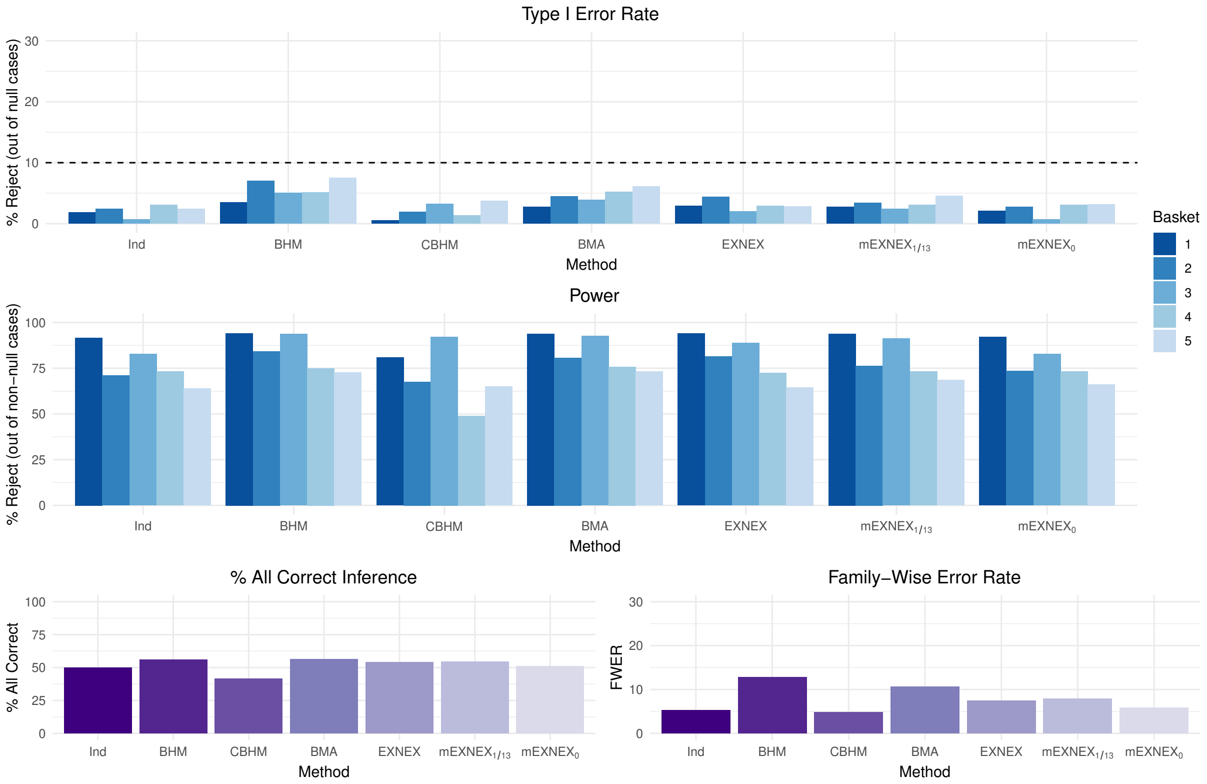

Figure S8: Operating characteristics under varied truths for the realized sample size case where 20, 10, 8, 18 and 7 patients are observed in the 5 baskets and Δ_α is re-calibrated

5 Evaluation of the 1-step mEXNEX$_c$ Models Compared to the Proposed 2-step mEXNEX$_c$ Model

The specification of the mEXNEX$_c$ model outlined in Section 2.6 of the main text, requires a two step procedure:

- Step 1: Remove clearly heterogeneous baskets to be analyzed independently, setting $\pi_k = 0$ in the EXNEX model. This is conducted based on some pre-defined cut-off value c which is compared to the minimum pair-wise difference in responses.

- Step 2: Of remaining baskets, compute pairwise Hellinger distances and set the prior borrowing probability π_k, to be the average of these distances (excluding the distance to itself).

Here we explore why the use of both of these steps is advantageous compared to making just one of these alterations to the standard EXNEX model. We consider four model settings:

1. The standard EXNEX model where $\pi_k = 0.5$ for all K baskets.
2. The EXNEX model with just step 2 i.e. no removal of heterogeneous baskets but Hellinger distances used to define the π_k values. Denote this as $\text{EXNEX}_{\text{Hell}}$.
3. The EXNEX model with just step 1, i.e. removing heterogeneous baskets and assigning remaining baskets a borrowing probability of 0.5. Denote this as EXNEXR_c.
4. The mEXNEX_c model as outlined in Section 2.6 which implements both steps.

5.1 Simulation Study Based on the Motivating VE-BASKET trial

To make such a comparison we initially consider the simulation setting outlined in Section 3 of the main text which is based on the motivating VE-BASKET trial. Results presented in Figure S9 are based on a planned and equal sample size of $n_k = 13$ patients in each of the k baskets and a cut-off of $c = 0$ implemented for the methods that require removal of heterogeneous baskets. Figure S9 displays the percentage of simulated data sets in which the null hypothesis is rejected for each basket and model setting under the data scenarios outlined in Table 1 of the main text. When the null is true, the bars represent the basket's type I error rate, else it is the power.

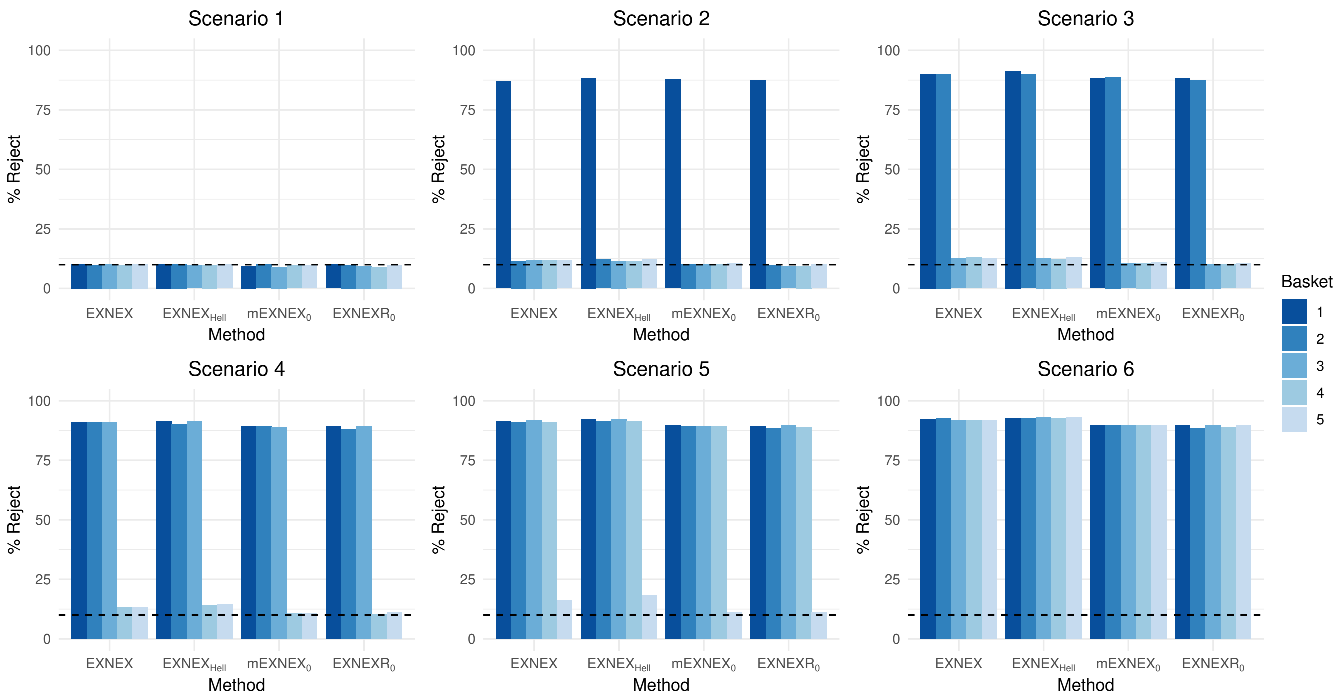

Figure S9: The percentage of simulated data sets in which the null hypothesis was rejected in each basket under the four model settings outlined above across the simulation settings provided in Table 1 of the main text.

From Figure S9 one can clearly see that when heterogeneous baskets are not removed from the borrowing component (i.e. in the EXNEX and $\text{EXNEX}_{\text{Hell}}$ models) an inflation in error rate is evident. Whereas, models that take this removal step have far better error control at the cost of slightly lower statistical power. We would therefore not recommend the $\text{EXNEX}_{\text{Hell}}$ for use as it's performance is inferior to the other proposed modifications.

Only minimal differences in the mEXNEX_c and EXNEXR_c are observed here with the mEXNEX_c method giving consistently greater power compared to the EXNEXR_c method but only up to an increase of 0.5%. This increase occurs alongside insubstantial inflation in error rates. Although the difference in these two methods is only slight in this single study, we explore further trial settings to investigate the differences between the two proposals.

5.2 Varying the Study Design

When conducting simulation studies there are a few design parameters to consider, these include:

- The number of baskets, K, included in the study.
- The sample size, n_k, within the k baskets.
- The null and target response rate.

In the simulation study in Section 5.1, the design parameters are specified to have $K = 5$ baskets with $n_k = 13$ patients in each of the $k = 1, \ldots, K$ baskets, while the null and target response rates are fixed at $q_0 = 0.15$ and $q_1 = 0.45$ respectively. These design parameters are in line with those of the VE-BASKET trial, which the simulation is motivated by. We now use this simulation as a reference setting, while we vary one of the three design parameters at a time to determine where differences in the mEXNEX$_c$ and EXNEXR$_c$, becomes more prominent. The different settings of design parameter alterations are provided in Table S14.

Table S14: Simulation settings for comparing the modified EXNEX models where we vary a single design parameter at a time.

Setting	No. Baskets (K)	Sample Size (n_k)	q_0	q_1
Reference	5	13	0.15	0.45
1	3	13	0.15	0.45
2	10	13	0.15	0.45
3	5	5	0.15	0.45
4	5	30	0.15	0.45
5	5	100	0.15	0.45
6	5	13	0.15	0.3
7	3	13	0.15	0.7

For the comparison of the design settings, the EXNEX$_{\text{Hell}}$ model was excluded due to it's inferior performance in the previous simulations. For both models that remove heterogeneous baskets we opt for two values of c, $c = 0.05$ and $c = 0.1$. The data scenario applied is one in which we have a ratio of two baskets with a response rate of q_1 to three baskets with a response rate of q_0.

Plotted in Figure S10 are the rejection percentages for each basket and model under the 8 different simulation settings outlined in Table S14. The first two of these settings vary the number of baskets from a very small number to moderately large, whilst settings 3-5 cover different possible sample sizes per basket, a case where the number of patients is very small, a moderate number and then in setting 5 a larger number of patients. The final two settings vary the target response rate with one value being close to the null response rate and the second being fairly different from q_0.

Looking first at the reference model, an average of 0.7% increase in power is observed with just a 0.3% increase in type I error when using mEXNEX$_c$ over EXNEXR$_c$ when $c = 0.05$. However, as the number of baskets, K, increases, the difference in type I error rate is more evident. The increased inflation in error rates across all methods as K increases comes about as less baskets are being treated independent at the removal step. To treat a basket as independent we compute all pairwise difference in response rates, i.e. for basket i, we treat it as independent if $|X_i - X_j| > c$ for all $i \neq j$ where $X_i = Y_i/n_i$, with Y_i being the number of responses observed in basket i which consists of n_i patients. If we consider the case where the response rates are IID, then the probability a basket is treated as independent is $\mathbb{P}(|X_1 - X_2| > c)^{K-1}$ which is decreasing as K increases. As a result, when $K = 10$, borrowing will occur more frequently between heterogeneous baskets and hence the error rates inflate to anywhere between 12 and 18% dependent on method chosen (compared to 10.5 to 12.4% when $K = 5$). When $K = 3$, more baskets are treated as independent and thus differences in error rate and power are minimal.

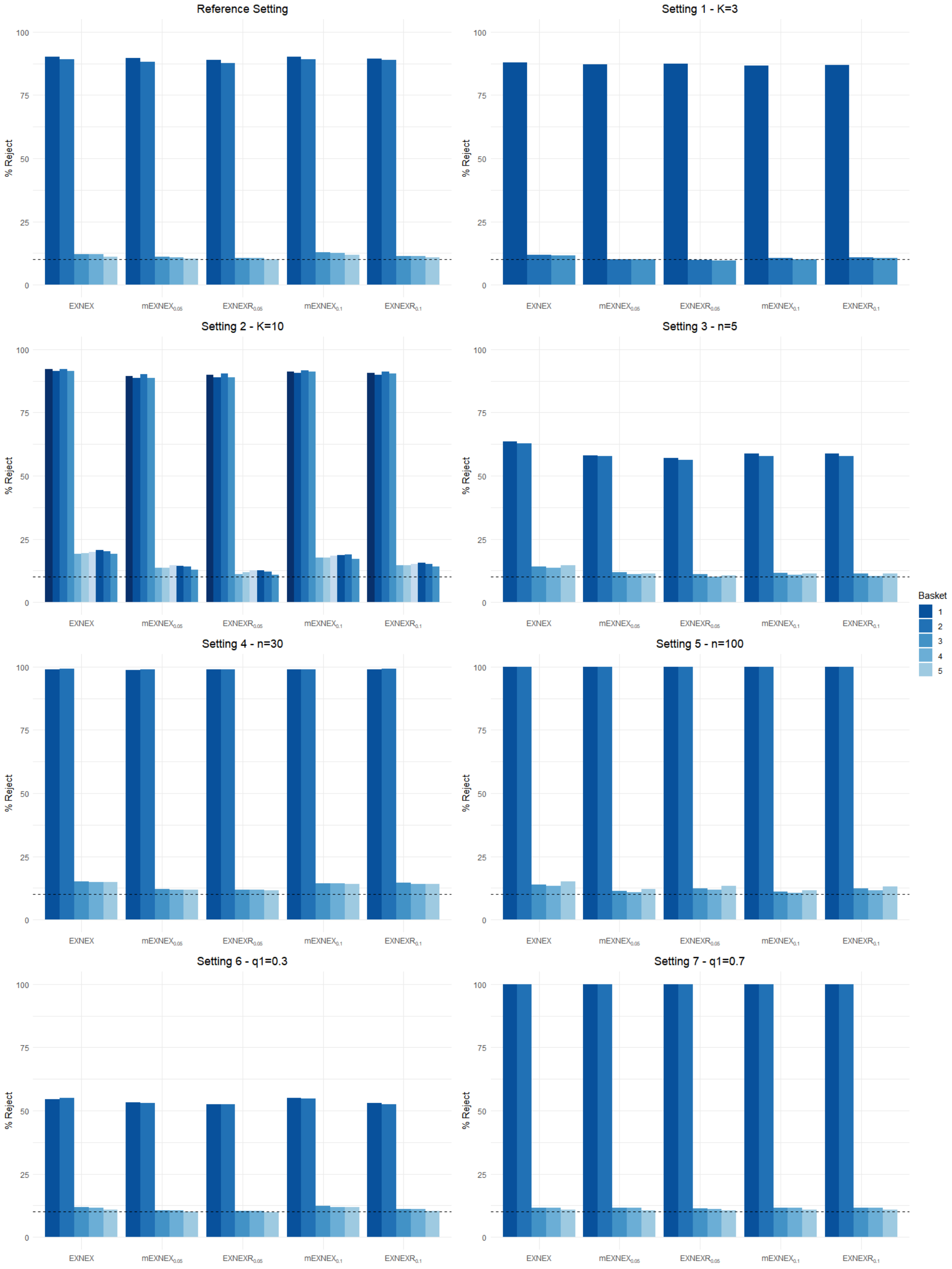

Figure S10: The percentage of simulated data sets in which the null hypothesis was rejected in each basket under the eight comparison settings outlined in Table S14.

More error rate inflation is observed when using mEXNEX_c compared to EXNEXR_c when $K = 10$ (up to a 2% increase when $c = 0.05$ and 3% when $c = 0.1$). This again comes down to fewer baskets being treated independently as despite this, the EXNEXR_c model limits borrowing by fixing the prior probabilities at $\pi_k = 0.5$, whereas under the mEXNEX_c model, these probabilities tend to be higher resulting in more borrowing from heterogeneous baskets and hence higher type I error rates.

Looking at the effect of sample size, we note that as the sample size increases, the Hellinger distance between two baskets decreases. These reduced Hellinger distances for both homogeneous and heterogeneous baskets, when averaged, result in smaller π_k values and hence less borrowing. In fact, as n_k increases to 100, these probabilities can actually fall below 0.5, so we therefore have a lower chance of borrowing between homogeneous baskets under the mEXNEX_c model compared to the EXNEXR_c model, and hence we observe better error control (11.45% compared to 12.48%). However, as the sample size increases, the increased certainty in estimates results in power tending towards 100% regardless of method and hence no improvement in power is observed. Even when n_k is small at just 5 patients in each basket, the difference in error rates is also relatviely small at a 0.8% increase under the mEXNEX_c model, whilst gaining on average 1.2% power when $c = 0.05$.

The final varied design parameter considered is the target response rate, q_1. When q_1 is closer to the null response rate, we observe minimal change in the type I error rate from the nominal 10% level, whereas, when $q_1 = 0.7$ this error rate rises to above 11% for both methods. Inflation in error rates is caused by a pull away from the true mean towards the common mean. When the target response rate is close to the null, this pull is less substantial compared to larger q_1 values, hence explaining the minimal difference in type I error rate when $q_1 = 0.3$. Similar error rates are observed under both methods, however the gain in power is greater under the mEXNEX_c model at 53.2% compared to 52.5% under the $\text{EXNEXR}_{0.05}$ model when q_1 is closer to the null response rate. This is due to the Hellinger distance, and hence π_k values, being closer to 1 under the mEXNEX_c model whereas under the EXNEXR_c model these are fixed at 0.5.

From the above we conclude that, in general, the mEXNEX_c model performs more favourably than EXNEXR_c when the sample size is very small or very large and when the target response rate is closer to the null response rate. Whereas, the EXNEXR_c model is preferred when the number of baskets increases and when the target response rate is very different to the null response rate. Overall, we would recommend the two-stage mEXNEX_c method over the EXNEXR_c model as a trial with fewer baskets and a target response rate closer to the null is more realistic. Although an argument could be made in some cases to use just the removal of heterogeneous baskets step.

6 Simulation Study for a Varied Number Baskets

In Section 5.2 a simulation study was conducted with one parameter varied at a time, this section now focuses in on just one of those parameters: the number of baskets, denoted K. Two values of K are considered - $K = 3$ and $K = 10$ - with full simulation results for several data scenarios and under each of the borrowing methods provided in Sections 6.1 and 6.2.

For both cases a total of 10,000 simulation runs were used for each method and data scenario. The VE-BASKET trial remains as the motivating example, as such, the sample size in each basket is fixed at $n_k = 13$ for all $k = 1, \ldots, K$ with a null and target response rate of $q_0 = 0.15$ and $q_0 = 0.45$ respectively. Model specifications are consistent with those outlined in Appendix A of the main text.

6.1 Simulation Study for $K = 3$ Baskets

Under $K = 3$ baskets, 8 data scenarios are considered and outlined in Table S15. Scenarios 1-4 cover varying number of effective baskets from none to all 3, whilst scenarios 5-8 consist of cases where some baskets are marginally effective with a true response rate of $p_k = 0.35$.

The modified EXNEX model was calibrated as outlined in the procedure in Section 2.6 of the main text and results for two values, $c = 1/13$ and $c = 4/13$, are presented here. The CBHM was also tuned to get parameters $a = -1.390$ and $b = 3.674$. Calibrated Δ_α values are provided in Table S16. Simulation results are presented in table form in Table S17, with rejection percentages also displayed in Figure S11.

Under data scenario 2, all methods give reasonably similar power values ranging from 86.9% to 88.4%. Both the BHM and BMA approach give the smallest power in this case with the $\text{mEXNEX}_{1/14}$ model presenting the highest. Error rates tend to be significantly smaller in the 3 basket case compared to the previous 5 basket simulation, as does the difference in performance between all methods. The BHM and BMA approach have inflated error rates at around 13%. Almost identical inflation is observed under the EXNEX and $\text{mEXNEX}_{4/14}$ model but the modified EXNEX model with $c = 1/13$ has lower error rates at 10.8% (compared to 11.5%). So the $\text{mEXNEX}_{1/13}$ model is appealing here for both it's error control and superior power value.

Table S15: Simulation study scenarios

	p_1	p_2	p_3
Scenario 1	0.15	0.15	0.15
Scenario 2	0.45	0.15	0.15
Scenario 3	0.45	0.45	0.15
Scenario 4	0.45	0.45	0.45
Scenario 5	0.35	0.15	0.15
Scenario 6	0.35	0.35	0.15
Scenario 7	0.45	0.35	0.15
Scenario 8	0.45	0.35	0.35

Table S16: Calibrated Δ_α values for the $K = 3$ basket simulation.

	Δ_α
Independent	0.904
BHM	0.862
CBHM	0.895
BMA	0.873
EXNEX	0.894
mEXNEX$_{1/13}$	0.916
mEXNEX$_{4/13}$	0.891

Across all scenarios 1-8 the standard EXNEX model and mEXNEX$_{4/14}$ model continue to perform almost identically with only marginal differences observed in terms of the type I error rate, power, FWER and percentage of all correct conclusions. However, when the cut-off is reduced to become more conservative at a value of $c = 1/13$, noticeable differences arise, including a slight reduction in error rates with a slight loss in power.

As in the previous simulation studies, the BHM and BMA procedure continue to inflate the type I error rate to an unacceptable level but in the 3 basket case, unlike the 5 basket case, tends to do little in terms of power improvement compared to the EXNEX models which possess superior error control. For example, under scenario 3 in which 2 of the 3 baskets are effective to treatment, power under the BHM is averaging at 90.9% with a type I error rate of 21.8%, whereas, under the EXNEX model power is 90.5% with a type I error rate of 11.6%. Therefore, one could argue that the minute power improvement can not justify the 10% increase in type I error rate. However, more improvement in power for the methods that borrow more strongly is observed in baskets with marginally effective response rates, most notably seen in scenario 8.

In a simulation akin to that in Section 4, a further study was conducted within which the true response rate, p, was randomly generated within each simulation run. Results of this is presented in Figure S12. Again, the standard EXNEX model and mEXNEX$_{4/13}$ model produce very similar results where both have type I error rate of 2.8%, but the mEXNEX$_{1/14}$ model has a slightly higher power at 85.9% compared to 85.7% under the EXNEX model. However, if the cut-off value c is reduced to 1/13 the error rate then becomes 2.7%, so a marginal decrease, with power 85.4%. Weighing up error control and power improvement one would favour the less conservative modified EXNEX approach or the standard EXNEX model as the error rates are all relatively similar but with power improvement over an independent analysis of about 2.5%.

All methods bar an independent analysis give approximately a 76% chance of making the correct conclusions across all baskets however, family-wise error rates vary. The BHM and BMA approach give FWER values of around 6.2% whilst the standard EXNEX and mEXNEX$_{4/13}$ models have a FWER of 4.7% with the mEXNEX$_0$ model slightly lower at 4.6%.

To summarise, the effect of reducing the number of baskets is the slight reduction in power alongside a smaller inflation in error rates particularly for the BHM and BMA approach, however, the conclusions regarding method comparison appear to be very similar to that in the planned sample size case with $K = 5$ baskets.

Table S17: Operating characteristics for a simulation consisting of $K = 3$ baskets.

	% Reject			% All Correct	FWER	% Reject			% All Correct	FWER
	**Scenario 1**					**Scenario 2**				
	**0.15**	**0.15**	**0.15**			**0.45**	**0.15**	**0.15**		
Independent	9.83	9.84	9.76	73.40	26.60	87.55	9.90	9.54	71.43	18.57
BHM	9.52	9.60	9.41	76.18	23.82	86.99	13.00	12.95	67.10	21.75
CBHM	9.79	9.69	9.52	76.05	23.95	87.30	12.82	12.67	66.77	21.95
BMA	9.37	9.71	9.39	76.74	23.26	86.86	14.51	14.12	66.22	22.48
EXNEX	9.97	10.22	9.84	73.93	26.07	88.03	11.56	11.49	68.34	21.66
$\text{mEXNEX}_{1/13}$	10.20	9.92	10.08	73.74	26.26	88.36	10.82	10.82	69.57	20.25
$\text{mEXNEX}_{4/13}$	9.94	10.14	9.84	74.04	25.96	88.31	11.52	11.42	68.62	21.55
	**Scenario 3**					**Scenario 4**				
	**0.45**	**0.45**	**0.15**			**0.45**	**0.45**	**0.45**		
Independent	87.78	87.44	9.77	69.27	9.77	87.94	87.21	87.84	67.97	
BHM	91.13	90.75	21.75	62.66	21.75	94.33	94.34	94.26	85.48	
CBHM	90.66	90.31	13.85	70.71	13.85	91.58	91.31	91.78	77.23	
BMA	91.53	91.27	21.98	62.72	21.98	94.47	94.23	94.59	85.70	
EXNEX	90.77	90.32	11.57	72.67	11.57	90.82	90.39	90.90	74.74	
$\text{mEXNEX}_{1/13}$	89.49	89.14	11.19	71.21	11.19	90.34	89.86	90.30	74.62	
$\text{mEXNEX}_{4/13}$	90.67	90.26	11.56	72.66	11.56	90.82	90.38	90.90	74.74	
	**Scenario 5**					**Scenario 6**				
	**0.35**	**0.15**	**0.15**			**0.35**	**0.35**	**0.15**		
Independent	67.9	9.88	9.76	55.48	18.64	67.83	66.33	9.71	40.52	9.71
BHM	66.34	12.60	12.52	50.19	21.19	73.04	71.63	19.94	38.04	19.94
CBHM	66.82	12.53	12.34	50.00	20.92	71.70	70.51	15.86	42.60	15.86
BMA	66.24	13.86	13.66	49.26	21.88	74.14	72.57	21.67	37.14	21.67
EXNEX	68.11	11.26	11.26	52.46	21.13	72.20	70.78	11.52	46.23	11.52
$\text{mEXNEX}_{1/13}$	68.80	10.69	10.41	53.66	19.73	70.06	68.63	11.06	45.09	11.06
$\text{mEXNEX}_{4/13}$	68.29	11.24	11.30	52.64	21.15	72.19	70.77	11.50	46.23	11.50
	**Scenario 7**					**Scenario 8**				
	**0.45**	**0.35**	**0.15**			**0.45**	**0.45**	**0.35**		
Independent	87.88	66.50	9.64	52.69	9.64	87.96	66.43	67.84	39.71	
BHM	90.64	72.50	20.68	48.48	20.68	93.59	79.47	80.26	65.73	
CBHM	90.12	71.68	14.67	54.89	14.67	91.92	74.29	75.54	54.26	
BMA	91.13	73.55	22.34	47.45	22.34	94.31	81.18	82.15	69.13	
EXNEX	90.40	71.26	11.53	57.47	11.53	90.71	71.46	72.85	47.69	
$\text{mEXNEX}_{1/13}$	89.41	69.69	11.01	56.12	11.01	89.81	69.86	71.30	47.64	
$\text{mEXNEX}_{4/13}$	90.30	71.31	11.55	57.47	11.55	90.67	71.44	72.82	47.59	

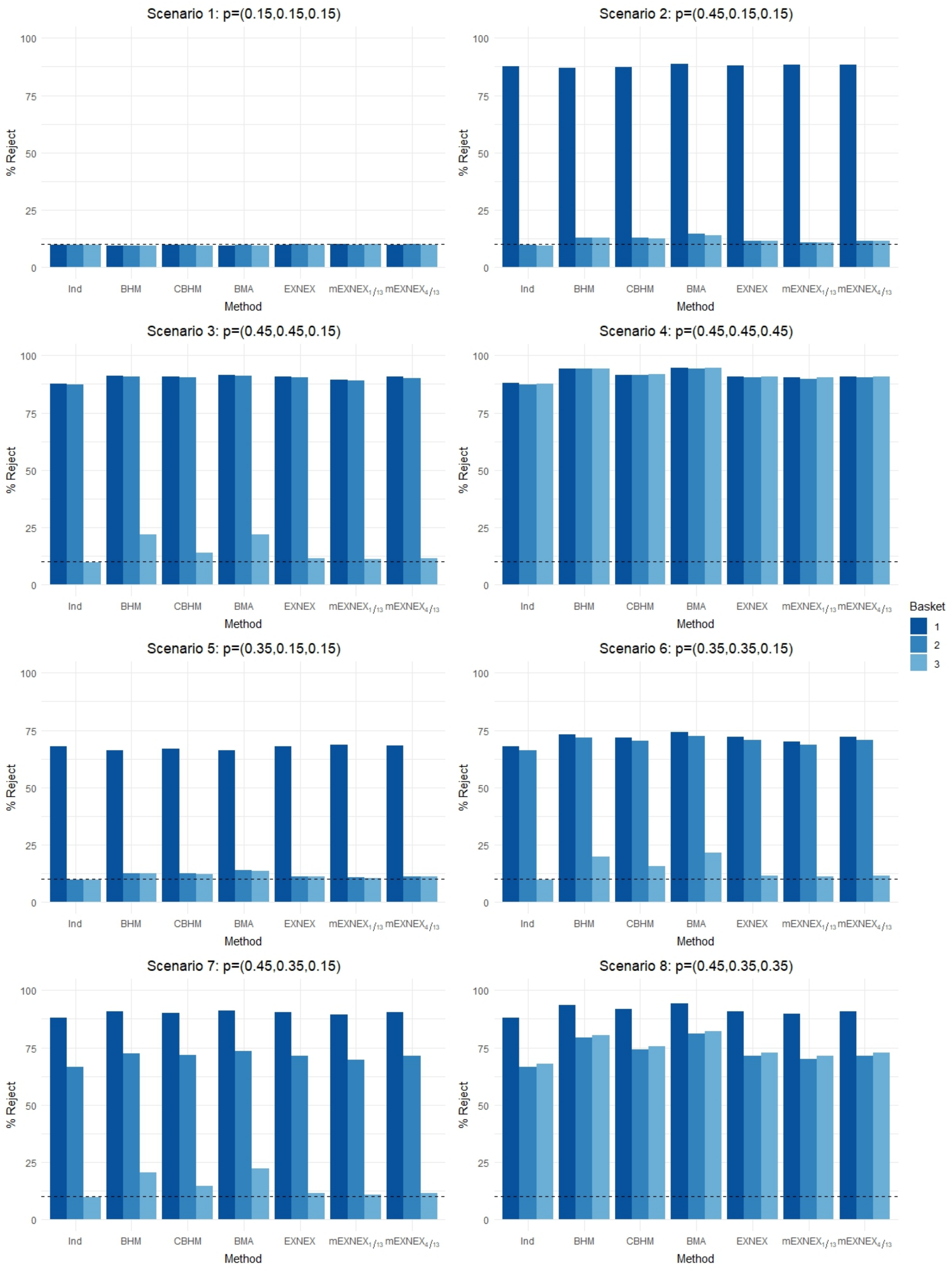

Figure S11: Percentage of rejections of the null hypothesis for each method under the 3 basket case.

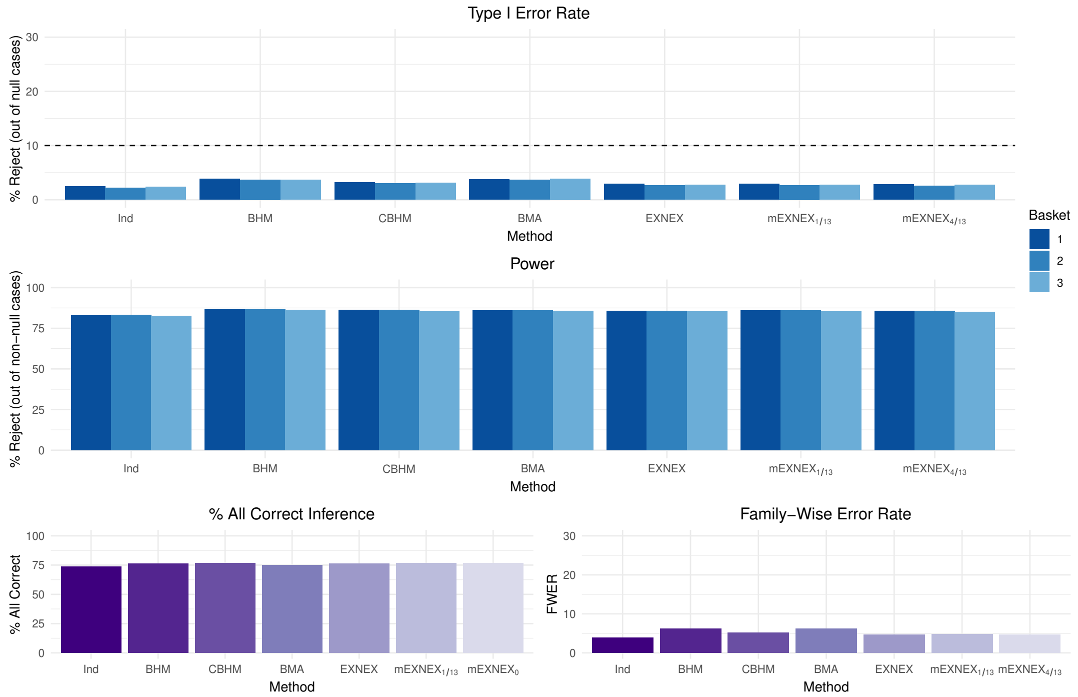

Figure S12: Operating characteristics under varied truths for the $K = 3$ basket simulation study

6.2 Simulation Study for $K = 10$ Baskets

Under $K = 10$ baskets, 15 data scenarios are considered and outlined in Table S18. Scenarios 1-11 cover varying number of effective baskets from 1-10, whilst scenarios 12-15 consist of cases where some baskets are marginally effective with a true response rate of $p_k = 0.35$.

Table S18: Simulation study scenarios

	p_1	p_2	p_3	p_4	p_5	p_6	p_7	p_8	p_9	p_10
Scenario 1	0.15	0.15	0.15	0.15	0.15	0.15	0.15	0.15	0.15	0.15
Scenario 2	0.45	0.15	0.15	0.15	0.15	0.15	0.15	0.15	0.15	0.15
Scenario 3	0.45	0.45	0.15	0.15	0.15	0.15	0.15	0.15	0.15	0.15
Scenario 4	0.45	0.45	0.45	0.15	0.15	0.15	0.15	0.15	0.15	0.15
Scenario 5	0.45	0.45	0.45	0.45	0.15	0.15	0.15	0.15	0.15	0.15
Scenario 6	0.45	0.45	0.45	0.45	0.45	0.15	0.15	0.15	0.15	0.15
Scenario 7	0.45	0.45	0.45	0.45	0.45	0.45	0.15	0.15	0.15	0.15
Scenario 8	0.45	0.45	0.45	0.45	0.45	0.45	0.45	0.15	0.15	0.15
Scenario 9	0.45	0.45	0.45	0.45	0.45	0.45	0.45	0.45	0.15	0.15
Scenario 10	0.45	0.45	0.45	0.45	0.45	0.45	0.45	0.45	0.45	0.15
Scenario 11	0.45	0.45	0.45	0.45	0.45	0.45	0.45	0.45	0.45	0.45
Scenario 12	0.35	0.35	0.15	0.15	0.15	0.15	0.15	0.15	0.15	0.15
Scenario 13	0.35	0.35	0.35	0.35	0.35	0.15	0.15	0.15	0.15	0.15
Scenario 14	0.45	0.45	0.45	0.35	0.35	0.35	0.15	0.15	0.15	0.15
Scenario 15	0.45	0.45	0.35	0.35	0.35	0.35	0.35	0.35	0.15	0.15

The modified EXNEX model was calibrated as outlined in the procedure in Section 2.6 of the main text and results of two calibrated cut-off values are presented here - $c = 0$ and $c = 1/13$. The CBHM was also tuned to get parameters $a = -23.475$ and $b = 10.963$. Calibrated Δ_α values are provided in Table S19.

Table S19: Calibrated Δ_α values for the $K = 10$ basket simulation.

	Δ_α
Independent	0.904
BHM	0.797
CBHM	0.896
BMA	0.844
EXNEX	0.833
mEXNEX$_0$	0.868
mEXNEX$_{1/13}$	0.841

Full results are presented in Tables S20, S21 and S22, with the hypothesis rejection percentages also displayed in Figures S13 and S14.

Consider scenario 2 where a single basket is heterogeneous and effective, the results are similar to that in the $K = 5$ basket case. Again, substantial inflation in the type I error rate is observed under the BHM and BMA approach, with an averaged error rate of 17.2% and 12.7% respectively. Under the $K = 5$ basket case, a similar scenario with a single heterogeneous basket resulted in an error rate of 16.9% and 13.2% respectively - these values are very similar indicating that increasing the number of baskets does little to eliminate error rate inflation under the two models that demonstrate the worst performance, particularly as in these scenarios where the power is substantially lower than an independent analysis (82.2% under the BHM compared to 88.3%).

Looking at other methods under the same scenario, the CBHM also has inflated error rates at approximately 12%. This contradicts the calibration nature of this model that takes a 'strong' definition of heterogeneity in that: if a single or multiple baskets have a heterogeneous response, then all are deemed heterogeneous so analysed as independent. In this scenario, there is clear heterogeneity thus it would be expected that the CBHM performs similarly to the independent model. This is not the case, leading to the conclusion that perhaps the calibration was slightly off.

The EXNEX model under scenario 2 also has fairly substantial error rates at 11.9% with a power of 87%, whereas,

under the more conservative modified EXNEX approach with $c = 0$, error rates are around 10.5%, so close to the nominal level, with a power of 87.5% - an improvement over the standard EXNEX model. If the cut-off was increased to $c = 1/13$, error rates increase to 11.3% with 87.4% power. Bringing together these results, under scenario 2, of the borrowing models the mEXNEX$_0$ model has both the highest power and best error control.

Moving on to cases in which multiple baskets are effective to the treatment (as in scenarios 3-10), the pattern of results described above hold in that the BHM and BMA inflate error rates, the mEXNEX$_c$ models have better error control than the standard EXNEX model, particularly when c is more conservative, whilst still improving power over an independent analysis. These are also the same conclusions drawn from the 5 basket simulation study presented in the main text.

One can see that the error rate gets far more substantial across all methods as the ratio of effective to ineffective baskets increases with the inflation greater than that in the 5 basket case. For example, under $K = 5$ the maximum error rate for the BHM is 42.1%, whereas in the $K = 10$ case this is 76.8% which occurs under scenarios 5 and 10 respectively. In scenario 10 for $K = 10$, just one basket is ineffective with a true response rate of 0.15 whilst the other 9 are effective with a higher response at 0.45. The presence of 9 effective basket compared to just 4 in the $K = 5$ case causes a larger pull up towards the common mean for the single heterogeneous basket, hence the greater inflation. This holds for all methods, with maximum error rates uniformly increasing from the $K = 5$ to $K = 10$ case. However, although this error inflation is observed, power is improved across all methods, this is unsurprising due to the additional certainty we gain from the extra 5 baskets included in the study.

Under the four scenarios in which a number of baskets have a marginally effective response rate of 35%, all borrowing methods show a substantial gain in power compared to an independent analysis, particularly for the marginally effective baskets. But inflation in error rates continues to be an issue.

As in Section 4 of the supplementary material, a further simulation was conducted within which the truth vector, p, is varied within each simulation. The results are provided in Figure S15. Unlike in the $K = 3$ basket case, more substantial differences are observed in the standard EXNEX model and the modified EXNEX approaches. The standard EXNEX model has error rates and power of 4.6% and 88.3% respectively, the mEXNEX$_0$ model has an average error rate of 3.3% and power 86.2% whilst the mEXNEX$_{1/13}$ model has 4.4% error rate and 88.1% power. All have power improvement over an independent analysis which has an average power of 83%, thus even under the most conservative modified EXNEX approach, power is improved by around 6.2%.

Then looking at the percentage of times correct inference was made across all 10 baskets, highest values were observed under the EXNEX model, mEXNEX$_{1/13}$ model, a BMA approach and the BHM at around 42%. The mEXNEX$_0$ model has value of 41.1% for all correct inference but the mEXNEX$_0$ model had 5% lower family-wise error rate compared to the standard EXNEX model. Weighing up both FWER and all correct inference, the mEXNEX$_0$ model appears optimal with FWER closer to that of the independent analysis and CBHM, whilst giving 3% higher percentage of correct inference compared to an independent analysis.

To summarise, this study confirms the conclusions drawn from the $K = 5$ basket simulation study presented in the main text whilst also highlighting that the larger number of baskets, although improving certainty of estimates and power, causes an even less favourable type I error rates.

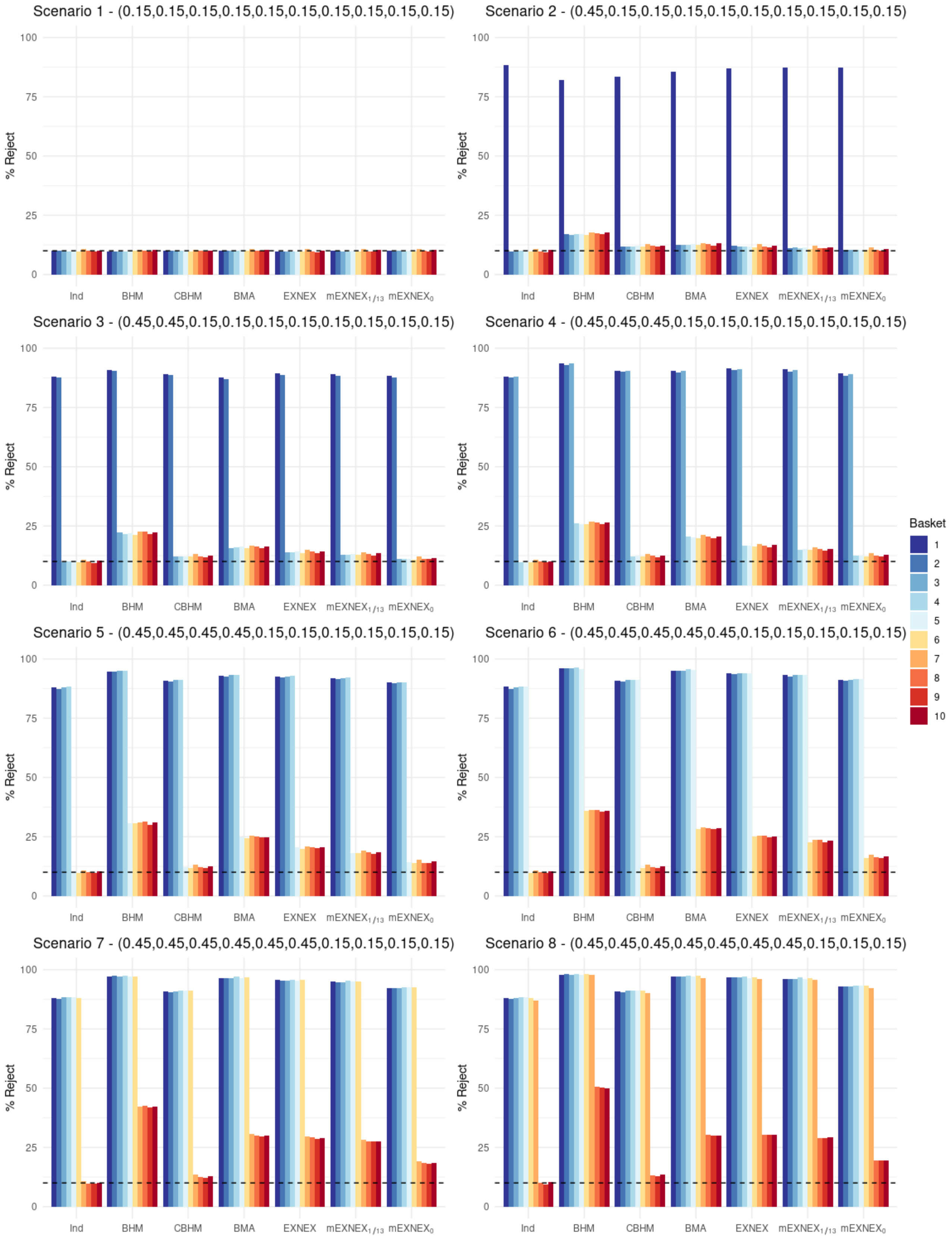

Figure S13: Percentage of rejections of the null hypothesis for each method under the 10 basket case across scenarios 1-8.

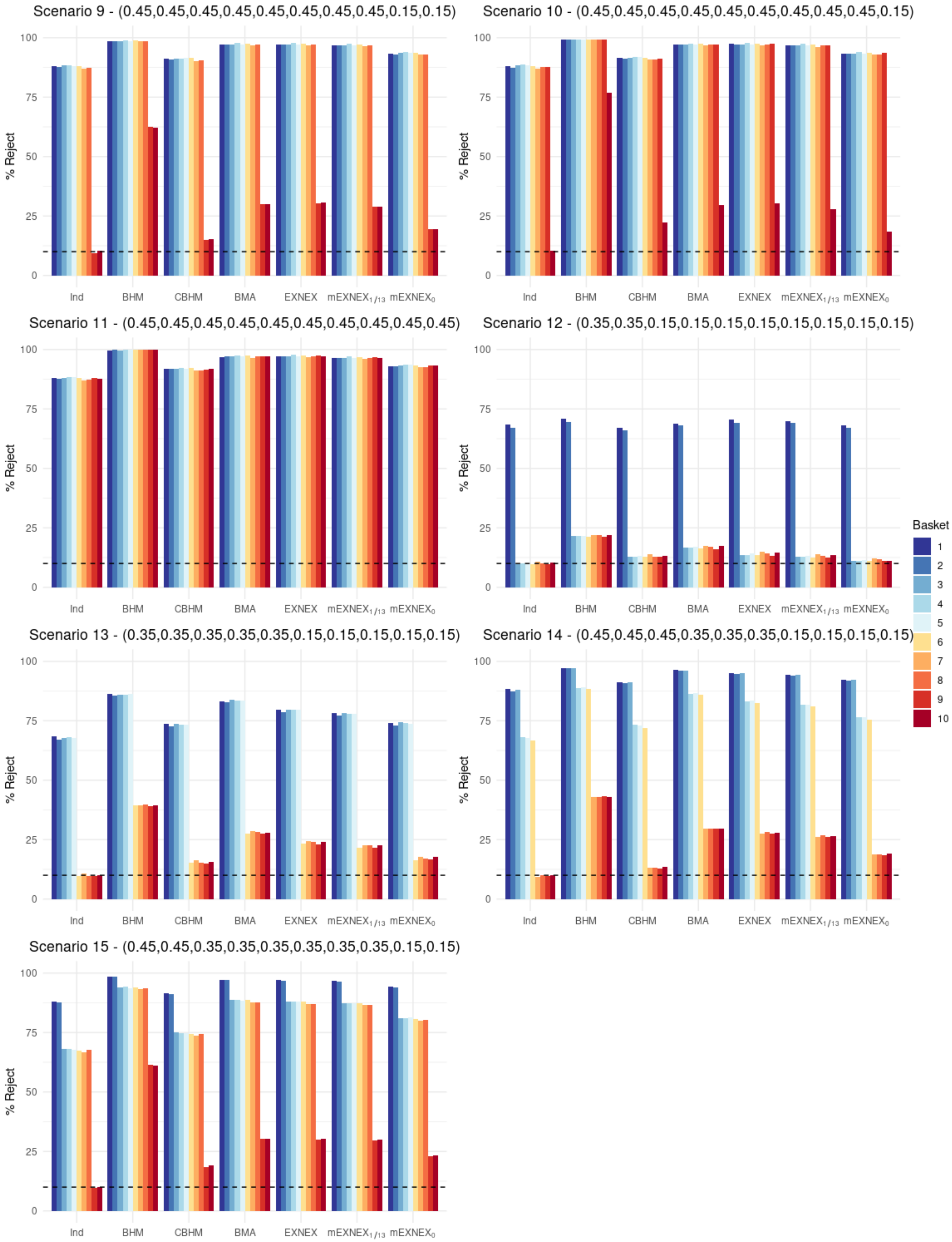

Figure S14: Percentage of rejections of the null hypothesis for each method under the 10 basket case across scenarios 9-15.

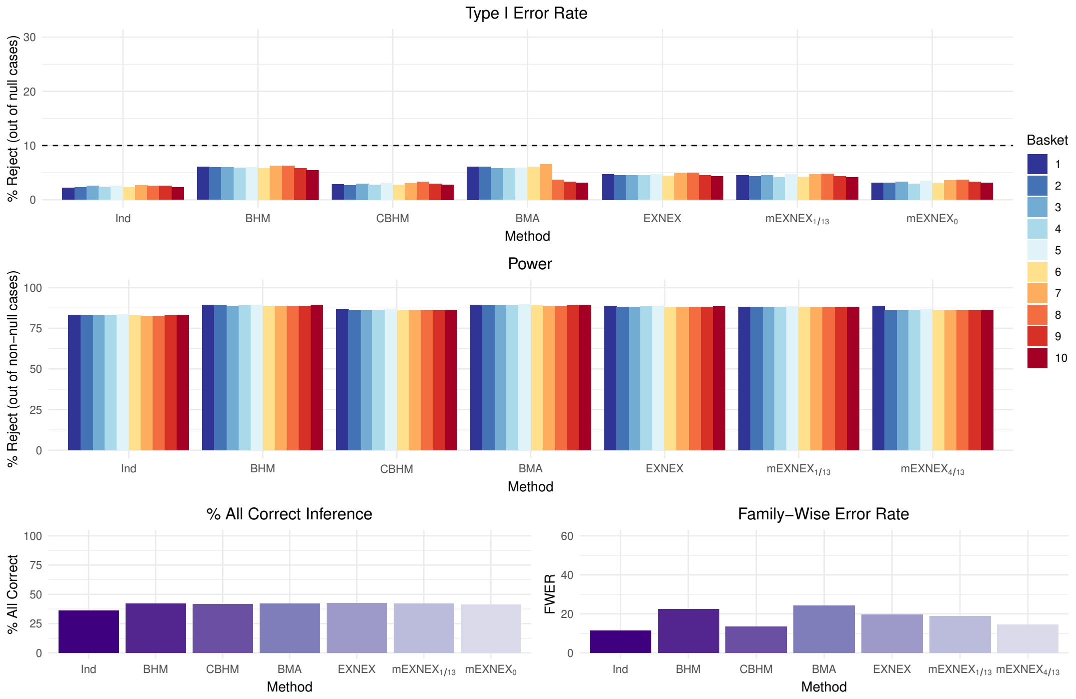

Figure S15: Operating characteristics under varied truths for the $K = 10$ basket simulation study

Table S20: Operating characteristics for a simulation based on $K = 10$ baskets with a sample size of $n_k = 13$ in each (scenarios 1-6)

	% Reject										% All Correct	FWER
Sample Size	13	13	13	13	13	13	13	13	13	13		
**Scenario 1**	**0.15**	**0.15**	**0.15**	**0.15**	**0.15**	**0.15**	**0.15**	**0.15**	**0.15**	**0.15**		
Independent	9.89	9.96	9.89	9.83	10.06	9.81	10.74	10.03	9.84	10.18	35.07	0.649
BHM	9.73	9.87	9.71	9.81	10.19	9.98	10.39	9.89	9.80	10.31	65.32	0.347
CBHM	10.21	10.14	9.88	9.91	10.19	9.80	10.55	10.14	9.75	10.09	59.35	0.407
BMA	10.08	10.17	9.99	10.14	10.39	10.16	10.85	9.91	10.06	10.51	54.78	0.452
EXNEX	9.67	9.59	9.71	9.74	9.78	9.60	10.62	9.53	9.50	10.18	49.15	0.509
$\text{mEXNEX}_{1/13}$	9.94	9.92	9.81	9.82	9.95	9.73	10.79	9.59	9.76	10.40	44.86	0.551
mEXNEX_0	10.05	9.81	9.95	9.81	9.97	9.60	10.82	9.96	9.87	10.25	36.39	0.636
**Scenario 2**	**0.45**	**0.15**	**0.15**	**0.15**	**0.15**	**0.15**	**0.15**	**0.15**	**0.15**	**0.15**		
Independent	88.34	9.80	9.84	9.88	9.97	9.65	10.71	9.85	9.51	10.40	34.97	0.608
BHM	82.15	16.95	16.85	17.12	17.17	16.77	17.67	17.45	16.90	17.78	25.42	0.596
CBHM	83.48	11.79	11.90	11.93	12.01	11.68	12.71	12.01	11.69	12.26	28.57	0.589
BMA	85.53	12.56	12.61	12.49	12.98	12.32	13.36	12.78	12.19	13.08	42.26	0.478
EXNEX	86.99	12.00	11.66	11.77	11.95	11.52	12.78	11.94	11.35	12.31	34.97	0.572
$\text{mEXNEX}_{1/13}$	87.43	11.26	11.29	11.17	11.35	10.81	12.09	11.06	10.96	11.50	35.93	0.570
mEXNEX_0	87.45	10.33	10.50	10.41	10.49	10.32	11.37	10.40	10.09	10.70	34.27	0.598
**Scenario 3**	**0.45**	**0.45**	**0.15**	**0.15**	**0.15**	**0.15**	**0.15**	**0.15**	**0.15**	**0.15**		
Independent	88.05	87.74	9.77	10.01	10.05	9.72	10.64	10.13	9.39	10.34	33.75	0.564
BHM	90.73	90.48	22.14	21.75	21.76	21.30	22.63	22.55	21.67	22.37	24.30	0.670
CBHM	89.05	88.74	12.24	12.00	12.23	12.01	13.15	12.26	11.84	12.58	29.69	0.606
BMA	87.82	86.99	15.79	16.05	16.28	15.75	16.84	16.34	15.51	16.49	31.44	0.528
EXNEX	89.52	88.72	13.87	13.80	14.18	13.59	14.99	14.28	13.51	14.28	28.73	0.605
$\text{mEXNEX}_{1/13}$	89.12	88.22	13.01	12.83	13.21	12.77	13.75	13.14	12.50	13.39	29.01	0.595
mEXNEX_0	88.35	87.56	11.26	11.07	11.24	10.91	11.97	11.22	11.06	11.43	32.04	0.563
**Scenario 4**	**0.45**	**0.45**	**0.45**	**0.15**	**0.15**	**0.15**	**0.15**	**0.15**	**0.15**	**0.15**		
Independent	88.05	87.51	87.87	9.69	9.88	9.67	10.77	9.91	9.63	10.01	32.76	0.518
BHM	93.52	93.02	93.48	26.27	25.73	25.61	26.74	26.51	25.85	26.35	21.91	0.696
CBHM	90.47	90.05	90.61	12.21	12.33	12.09	13.22	12.37	11.96	12.56	31.38	0.574
BMA	90.39	89.72	90.44	20.48	20.12	19.95	21.08	20.61	19.71	20.67	24.62	0.585
EXNEX	91.43	90.79	91.30	16.55	16.56	16.26	17.45	16.86	16.02	16.90	20.29	0.593
$\text{mEXNEX}_{1/13}$	90.99	90.09	90.75	14.83	15.13	14.84	15.92	15.33	14.63	15.33	29.84	0.580
mEXNEX_0	89.25	88.46	89.10	12.33	12.59	12.20	13.46	12.43	12.09	12.88	30.93	0.543
**Scenario 5**	**0.45**	**0.45**	**0.45**	**0.45**	**0.15**	**0.15**	**0.15**	**0.15**	**0.15**	**0.15**		
Independent	88.16	87.39	88.09	88.24	10.06	9.64	10.81	10.04	9.75	10.22	31.38	0.472
BHM	94.76	94.59	94.89	95.13	30.73	30.71	31.07	31.27	30.03	31.01	17.54	0.733
CBHM	90.79	90.37	90.99	91.13	12.18	11.83	13.11	12.11	11.81	12.36	31.92	0.524
BMA	92.98	92.45	93.12	93.10	24.87	24.51	25.38	25.02	24.58	24.81	19.50	0.655
EXNEX	92.50	92.23	92.51	92.80	20.40	19.92	20.99	20.53	20.02	20.43	27.11	0.587
$\text{mEXNEX}_{1/13}$	91.90	91.45	91.83	92.08	18.18	17.99	19.14	18.30	17.86	18.39	29.14	0.557
mEXNEX_0	90.16	89.77	90.19	90.22	14.41	13.90	15.27	14.02	13.72	14.56	29.98	0.520
**Scenario 6**	**0.45**	**0.45**	**0.45**	**0.45**	**0.45**	**0.15**	**0.15**	**0.15**	**0.15**	**0.15**		
Independent	88.44	87.38	88.15	88.38	88.49	9.61	10.79	10.05	9.63	10.37	31.21	0.413
BHM	96.04	96.06	96.16	96.34	95.88	35.96	36.30	36.24	35.57	35.98	15.50	0.766
CBHM	90.88	90.50	91.04	91.17	91.16	11.86	13.09	12.08	11.74	12.45	32.82	0.467
BMA	95.17	94.97	95.15	95.55	95.28	28.31	28.96	28.60	28.07	28.45	14.57	0.742
EXNEX	93.96	93.49	93.90	94.02	93.92	24.91	25.59	25.46	24.72	25.09	20.22	0.641
$\text{mEXNEX}_{1/13}$	93.17	92.73	93.14	93.32	93.34	22.62	23.55	23.67	22.77	23.18	23.05	0.594
mEXNEX_0	91.31	90.80	91.18	91.54	91.39	16.01	17.29	16.34	15.90	16.68	29.14	0.504

Table S21: Operating characteristics for a simulation based on $K = 10$ baskets with a sample size of $n_k = 13$ in each (scenarios 7-12)

	% Reject										% All Correct	FWER
Sample Size	13	13	13	13	13	13	13	13	13	13		
**Scenario 7**	**0.45**	**0.45**	**0.45**	**0.45**	**0.45**	**0.45**	**0.15**	**0.15**	**0.15**	**0.15**		
Independent	88.13	87.51	88.33	88.20	88.24	88.05	10.64	9.74	9.55	10.05	30.97	0.344
BHM	97.07	97.32	97.18	97.47	97.07	97.24	42.30	42.55	41.80	42.14	17.43	0.767
CBHM	90.89	90.51	90.98	91.23	91.09	91.06	13.47	12.55	12.14	12.80	33.58	0.399
BMA	96.49	96.42	96.55	96.99	96.45	96.67	30.74	30.14	29.75	29.86	18.46	0.746
EXNEX	95.59	95.23	95.33	95.82	95.38	95.67	29.66	29.26	28.63	28.88	16.83	0.709
$\text{mEXNEX}_{1/13}$	94.84	94.69	94.61	95.19	94.85	95.02	28.09	27.61	27.38	27.64	17.91	0.667
mEXNEX_0	92.19	92.06	92.15	92.59	92.48	92.43	19.17	18.41	18.00	18.46	29.39	0.478
**Scenario 8**	**0.45**	**0.45**	**0.45**	**0.45**	**0.45**	**0.45**	**0.45**	**0.15**	**0.15**	**0.15**		
Independent	88.06	87.51	87.95	88.20	88.48	87.99	87.06	9.92	9.52	10.28	29.76	0.268
BHM	97.80	98.00	97.94	98.19	97.78	98.00	97.65	50.60	50.32	50.04	20.19	0.737
CBHM	90.98	90.57	91.07	91.24	91.19	91.07	89.99	13.09	12.81	13.44	34.78	0.313
BMA	97.07	96.98	97.01	97.51	96.94	97.37	96.56	30.38	30.07	30.00	27.89	0.653
EXNEX	96.70	96.62	96.70	97.18	96.51	96.92	96.22	30.40	30.16	30.25	26.54	0.654
$\text{mEXNEX}_{1/13}$	96.11	96.15	96.19	96.71	96.11	96.51	95.82	29.08	29.01	29.12	27.14	0.625
mEXNEX_0	93.03	92.81	92.92	93.31	93.37	93.14	92.21	19.41	19.42	19.48	32.31	0.421
**Scenario 9**	**0.45**	**0.45**	**0.45**	**0.45**	**0.45**	**0.45**	**0.45**	**0.45**	**0.15**	**0.15**		
Independent	87.94	87.53	88.31	88.41	88.10	88.05	87.00	87.45	9.46	10.32	29.22	0.187
BHM	98.51	98.53	98.51	98.81	98.45	98.73	98.46	98.49	62.33	62.06	17.69	0.760
CBHM	91.08	90.69	91.28	91.32	91.34	91.34	90.24	90.46	15.04	15.44	34.04	0.242
BMA	97.13	97.11	97.16	97.69	96.99	97.51	96.69	97.19	29.99	29.95	39.12	0.506
EXNEX	97.20	97.11	97.21	97.70	97.08	97.50	96.78	97.21	30.40	30.50	38.78	0.515
$\text{mEXNEX}_{1/13}$	96.70	96.67	96.83	97.38	96.70	97.14	96.30	96.79	28.86	28.90	39.34	0.487
mEXNEX_0	93.20	92.98	93.48	93.86	93.47	93.50	92.88	93.00	19.38	19.45	36.18	0.327
**Scenario 10**	**0.45**	**0.45**	**0.45**	**0.45**	**0.45**	**0.45**	**0.45**	**0.45**	**0.45**	**0.15**		
Independent	88.19	87.37	88.26	88.59	88.25	88.15	87.13	87.59	87.74	10.23	28.38	0.102
BHM	99.18	99.24	99.31	99.28	99.26	99.28	99.20	99.21	99.34	76.82	18.83	0.768
CBHM	91.53	91.21	91.61	91.88	91.70	91.68	90.71	90.91	91.30	22.24	29.94	0.222
BMA	97.06	97.07	97.09	97.62	97.02	97.46	96.72	97.14	97.28	29.62	54.14	0.296
EXNEX	97.29	97.22	97.24	97.77	97.12	97.55	96.85	97.26	97.42	30.49	54.31	0.305
$\text{mEXNEX}_{1/13}$	96.65	96.66	96.75	97.33	96.67	97.10	96.21	96.78	96.92	28.02	54.14	0.280
mEXNEX_0	93.2	93.11	93.35	93.97	93.40	93.50	92.82	92.95	93.64	18.45	41.18	0.185
**Scenario 11**	**0.45**	**0.45**	**0.45**	**0.45**	**0.45**	**0.45**	**0.45**	**0.45**	**0.45**	**0.45**		
Independent	88.03	87.60	88.06	88.52	88.24	88.06	87.08	87.36	88.00	87.82	27.50	
BHM	99.64	99.79	99.72	99.78	99.74	99.79	99.83	99.78	99.76	99.82	98.03	
CBHM	91.72	91.70	91.84	92.38	91.84	92.12	91.19	91.27	91.67	91.95	57.50	
BMA	96.88	97.00	96.97	97.50	96.95	97.41	96.56	97.05	97.22	97.03	74.24	
EXNEX	97.26	97.21	97.27	97.77	97.13	97.56	96.85	97.28	97.43	97.20	76.07	
$\text{mEXNEX}_{1/13}$	96.27	96.35	96.45	96.96	96.35	96.83	95.97	96.43	96.70	96.40	68.66	
mEXNEX_0	92.96	92.85	93.19	93.58	93.49	93.36	92.57	92.69	93.41	93.11	46.12	
**Scenario 12**	**0.35**	**0.35**	**0.15**	**0.15**	**0.15**	**0.15**	**0.15**	**0.15**	**0.15**	**0.15**		
Independent	68.34	66.92	9.82	9.89	9.87	9.79	10.72	9.94	9.69	10.25	20.25	0.567
BHM	70.88	69.50	21.52	21.58	21.51	21.13	21.84	21.79	21.11	21.91	13.30	0.609
CBHM	66.90	66.10	12.89	12.98	13.02	12.88	13.75	12.94	12.88	13.35	17.28	0.549
BMA	68.66	67.95	16.58	16.60	16.90	16.30	17.43	17.10	16.10	17.33	14.68	0.544
EXNEX	70.49	69.26	13.69	13.66	14.21	13.62	14.87	14.11	13.29	14.69	16.91	0.594
$\text{mEXNEX}_{1/13}$	69.93	69.02	12.96	12.77	13.08	12.65	13.91	13.16	12.46	13.44	17.33	0.581
mEXNEX_0	68.10	67.00	21.02	11.23	11.27	11.11	12.04	11.63	11.02	11.24	19.29	0.552

Table S22: Operating characteristics for a simulation based on $K = 10$ baskets with a sample size of $n_k = 13$ in each (scenarios 13-15)

	% Reject										% All Correct	FWER
Sample Size	13	13	13	13	13	13	13	13	13	13		
**Scenario 13**	**0.35**	**0.35**	**0.35**	**0.35**	**0.35**	**0.15**	**0.15**	**0.15**	**0.15**	**0.15**		
Independent	68.46	67.02	67.76	68.16	67.79	9.53	10.74	9.87	9.62	10.05	8.82	0.410
BHM	86.13	85.42	85.97	86.07	86.36	39.53	39.31	39.61	39.25	39.33	6.42	0.750
CBHM	73.76	72.59	73.82	73.47	73.35	15.35	16.28	15.44	15.01	15.59	10.58	0.475
BMA	83.26	82.73	83.70	83.44	83.48	27.63	28.44	28.22	27.52	28.01	5.49	0.730
EXNEX	79.66	78.58	79.76	79.68	79.64	23.43	24.52	24.10	23.13	24.20	5.84	0.618
$\text{mEXNEX}_{1/13}$	78.38	77.28	78.20	78.05	77.95	21.60	22.71	22.46	21.67	22.68	6.38	0.581
mEXNEX_0	74.20	73.13	74.30	74.05	73.64	16.46	17.60	16.97	16.64	17.60	8.89	0.501
**Scenario 14**	**0.45**	**0.45**	**0.45**	**0.35**	**0.35**	**0.35**	**0.15**	**0.15**	**0.15**	**0.15**		
Independent	88.42	87.47	88.13	68.06	67.87	66.54	9.44	10.03	9.82	10.06	13.82	0.339
BHM	96.94	96.99	97.14	88.71	89.20	88.28	43.01	43.03	43.09	42.93	11.64	0.754
CBHM	91.13	90.79	91.26	73.36	73.12	71.76	13.28	13.33	12.91	13.61	16.89	0.396
BMA	96.39	96.21	96.22	86.40	86.58	85.83	29.49	29.79	29.58	29.73	13.27	0.731
EXNEX	95.06	94.74	94.94	83.16	83.48	82.55	27.68	28.11	27.59	27.96	10.54	0.675
$\text{mEXNEX}_{1/13}$	94.46	94.02	94.33	81.74	81.84	81.08	26.24	26.78	26.26	26.58	9.54	0.636
mEXNEX_0	92.07	91.91	92.07	76.64	76.35	75.30	18.62	18.69	18.44	19.02	14.92	0.476
**Scenario 15**	**0.45**	**0.45**	**0.35**	**0.35**	**0.35**	**0.35**	**0.35**	**0.35**	**0.15**	**0.15**		
Independent	88.02	87.77	68.18	67.92	67.62	67.49	66.63	67.74	9.54	10.20	6.58	0.187
BHM	98.58	98.62	93.90	94.20	93.70	93.88	93.34	93.64	61.48	61.00	9.95	0.746
CBHM	91.63	91.24	75.06	74.19	74.75	74.31	73.55	74.24	18.52	19.02	8.54	0.273
BMA	97.22	97.12	88.65	88.71	88.50	88.63	87.68	87.82	30.42	30.47	22.09	0.515
EXNEX	96.94	96.73	87.99	87.99	87.93	87.95	87.09	87.04	30.14	30.21	22.03	0.509
$\text{mEXNEX}_{1/13}$	96.59	96.54	87.37	87.31	87.23	87.25	86.51	86.53	29.27	29.82	21.89	0.502
mEXNEX_0	94.29	93.93	80.98	81.09	81.28	80.66	80.02	80.43	22.97	23.40	14.73	0.388
